# Human-Specific Evolution and Adaptation Led to Major Qualitative Differences in the Variable Receptors of Human and Chimpanzee Natural Killer Cells

**DOI:** 10.1371/journal.pgen.1001192

**Published:** 2010-11-04

**Authors:** Laurent Abi-Rached, Achim K. Moesta, Raja Rajalingam, Lisbeth A. Guethlein, Peter Parham

**Affiliations:** 1 Department of Structural Biology, Stanford University School of Medicine, Stanford, California, United States of America; 2 UCLA Immunogenetics Center, Department of Pathology and Laboratory Medicine, David Geffen School of Medicine at UCLA, University of California Los Angeles, Los Angeles, California, United States of America; Fred Hutchinson Cancer Research Center, United States of America

## Abstract

Natural killer (NK) cells serve essential functions in immunity and reproduction. Diversifying these functions within individuals and populations are rapidly-evolving interactions between highly polymorphic major histocompatibility complex (MHC) class I ligands and variable NK cell receptors. Specific to simian primates is the family of Killer cell Immunoglobulin-like Receptors (KIR), which recognize MHC class I and associate with a range of human diseases. Because KIR have considerable species-specificity and are lacking from common animal models, we performed extensive comparison of the systems of KIR and MHC class I interaction in humans and chimpanzees. Although of similar complexity, they differ in genomic organization, gene content, and diversification mechanisms, mainly because of human-specific specialization in the KIR that recognizes the C1 and C2 epitopes of MHC-B and -C. Humans uniquely focused KIR recognition on MHC-C, while losing C1-bearing MHC-B. Reversing this trend, C1-bearing HLA-B46 was recently driven to unprecedented high frequency in Southeast Asia. Chimpanzees have a variety of ancient, avid, and predominantly inhibitory receptors, whereas human receptors are fewer, recently evolved, and combine avid inhibitory receptors with attenuated activating receptors. These differences accompany human-specific evolution of the *A* and *B* haplotypes that are under balancing selection and differentially function in defense and reproduction. Our study shows how the qualitative differences that distinguish the human and chimpanzee systems of KIR and MHC class I predominantly derive from adaptations on the human line in response to selective pressures placed on human NK cells by the competing needs of defense and reproduction.

## Author Summary

Natural killer (NK) cells are versatile lymphocytes that make essential contributions to immune defense and placental reproduction. Essential to NK cell development, diversification and function are variable families of surface receptors that recognize equally variable determinants of polymorphic major histocompatibility complex (MHC) class I molecules, better known as the tissue types matched in clinical organ transplantation. These ligand-receptor interactions evolve rapidly, exhibiting much species specificity and convergent evolution. Consequently, mice represent a poor model, because their receptors are so disparate from the independently evolved human counterparts that are restricted to simian primates. To identify unique and shared aspects of human NK cell biology, we have defined the genomics, population biology, and immunology of variable chimpanzee NK cell receptors and ligands to a level permitting accurate, informed comparison with the well-characterized human system. In both receptors and ligands there are dramatic, qualitative differences between humans and chimpanzees. We show these differences arose during human evolution from the last common human–chimpanzee ancestor, while the chimpanzee system remained relatively stable. That two so closely related species exhibit major differences in NK cell receptors and ligands testifies to the strong and varying selection imposed by the different demands and competing needs of defense and reproduction.

## Introduction

Natural killer (NK) cells are lymphocytes that contribute to both the immune and reproductive systems. NK cells provide first-line, innate immune defense against infection [1] and cancer [2], and through interaction with dendritic cells [3] help initiate the second-line, adaptive immune response [4]. During embryo implantation and placentation, NK cells control the trophoblast-mediated widening of maternal blood vessels necessary to nourish the fetus throughout pregnancy [5]. Controlling both NK cell development and effector function is a variety of interactions between NK cell receptors and their ligands [6], the class I molecules of the major histocompatibility complex (MHC): called the HLA complex in humans. Some interactions are conserved, such as that between human HLA-E and the CD94:NKG2A receptor [7], whereas others are highly variable, notably those between HLA-A, B, C and killer cell immunoglobulin-like receptors (KIR) [8]. Pointing to the clinical importance of these interactions, various combinations of HLA and KIR factors associate with the outcome of viral infection, susceptibility to autoimmune disease, relapse of leukemia following therapeutic transplantation, and reproductive success [9]–[11].

The human *KIR* locus combines gene content variability with allelic polymorphism [8], [12]. This diverse family of NK cell receptor genes is restricted to simian primates, having expanded from a single copy *KIR3DL* gene during the last ∼40–58 million years [13]. In rodents, where *KIR* genes are expressed in the brain, but not by NK cells [14], the *Ly49* gene family independently evolved as a variable family of NK cell receptors for MHC class I [15]. Prosimians have a single, non-functional *KIR3DL* gene, but a diversified system of *CD94* and *NKG2* genes [13]. Also having a single *KIR3DL* gene, cattle expanded and diversified the distantly related *KIR3DX* gene [16], which in humans is non-functional. This strong element of species-specific evolution likely reflects the variety and inconstancy of selection imposed on NK cells by immune defense and reproduction; the former being essential for individuals to survive, the latter being necessary for the survival of populations and species [17]. In this context of rapidly evolving NK cell receptors, the study of chimpanzees, our closest living relatives, becomes an imperative, not only for clinical studies in which the chimpanzee is the preferred animal model, for example hepatitis C virus infection [18], but also for defining those aspects of NK cell function that are unique to the human species [19].

HLA-A, B, C and G serve as ligands for human KIR [8]. HLA-G expression is restricted to trophoblast and thus dedicated to functions associated with pregnancy [20]. Of the highly polymorphic genes, only HLA-C is present on trophoblast and able to interact with the KIR of uterine NK cells [21]. HLA-A, B and C are expressed by almost all cells of the body and can thus contribute in general to NK cell responses against infection and cancer. Although the chimpanzee has well-defined orthologs of all the human *HLA class I* genes [22], exploratory studies of chimpanzee *KIR* cDNA and one *KIR* haplotype [23], [24], raised intriguing possibilities: first that only a small minority of *KIR* genes is shared by humans and chimpanzees; and second, that the organization of *KIR* genes into haplotypes is qualitatively different in the two species. To test these hypotheses we performed extensive analysis of chimpanzee *KIR* haplotype structure and variation, permitting definitive genetic and functional comparison with the human *KIR* system.

## Results

### Chimpanzee *KIR* haplotypes do not divide into functional groups like human *A* and *B* haplotypes

**Figure 1:**
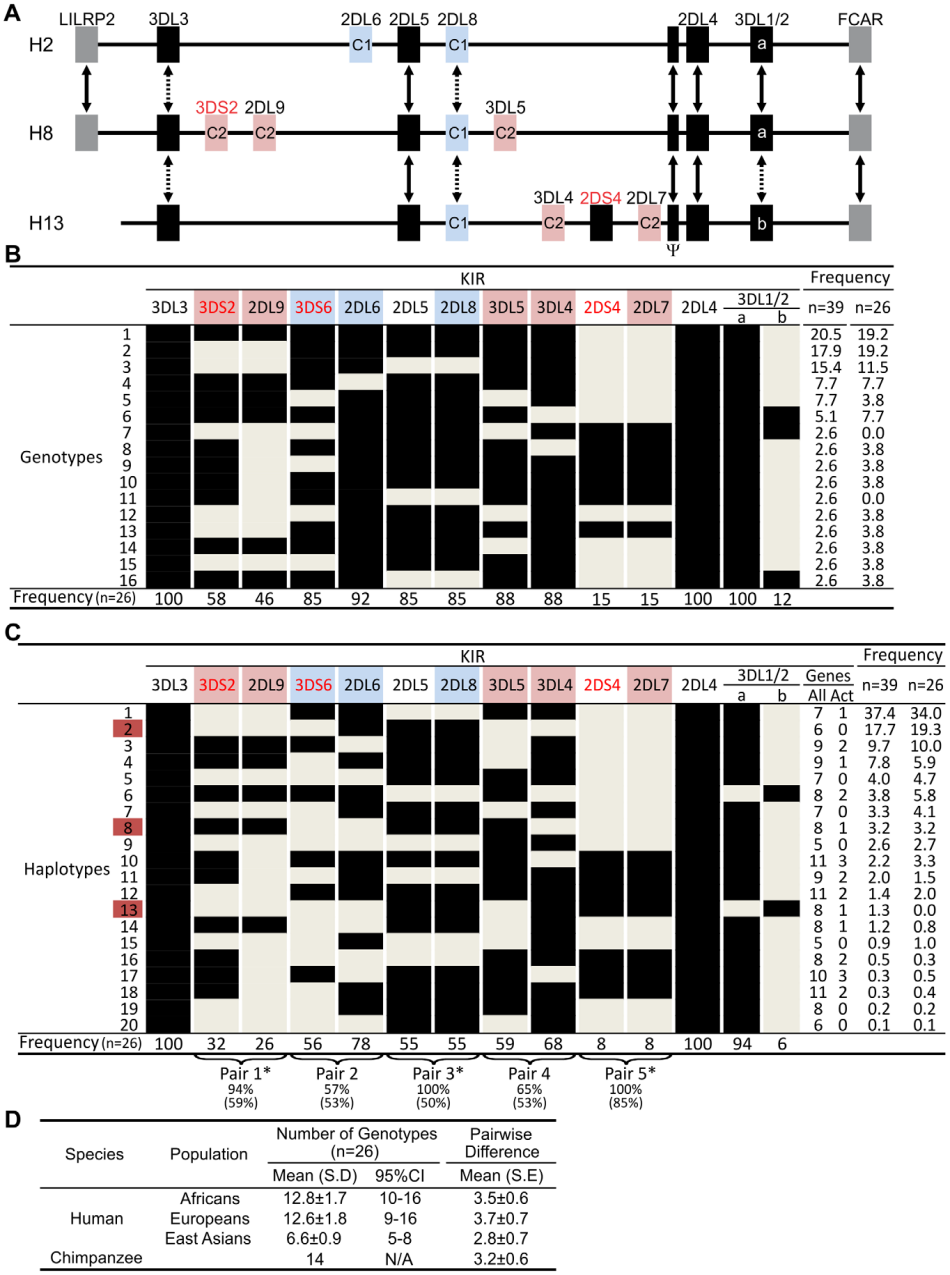
Chimpanzee and human *KIR* genotypes are comparably diverse. (A) Shows *KIR* gene content of three chimpanzee haplotypes. Arrows indicate equivalent genes; dotted arrows, divergent alleles. Lineage III KIR are colored blue (MHC-C1 specific) or pink (MHC-C2 specific); names of activating KIR are red. Ψ, *KIR* pseudogene. Haplotype names are from panel (C). The flanking non-*KIR* genes are colored gray. (B) *KIR* gene content was assessed in 39 chimpanzees. The 16 distinct genotypes characterized and their frequencies are given here. Thirteen *KIR* loci defined by analysis of cDNA [23] and of the three haplotype sequences of panel A were investigated. *KIR* phenotype and genotype frequencies are also given for the subgroup of 26 unrelated individuals. (C) Component *KIR* haplotypes were deduced from the genotype data presented in panel B, and presented here with their estimated frequencies. *KIR* gene and haplotype frequencies are also given for the subgroup of 26 unrelated individuals. ‘Genes’ gives the number of *KIR* per haplotype (‘All’), and the number of activating receptor genes (‘Act’). Chimpanzee *KIR* haplotype diversity stems from recombination involving five pairs of variable *KIR*: for each pair the top percentage indicates the observed ‘pairing frequency’ (genes both present or both absent) and the bottom percentage is the expected ‘pairing frequency’ under random distribution. *, significant linkage disequilibrium (p<0.001). Red boxes denote the sequenced haplotypes. (D) Both in number and gene content difference, chimpanzee *KIR* genotypes are within the human range (see Materials and Methods for details). CI, confidence interval.

**Figure 2:**
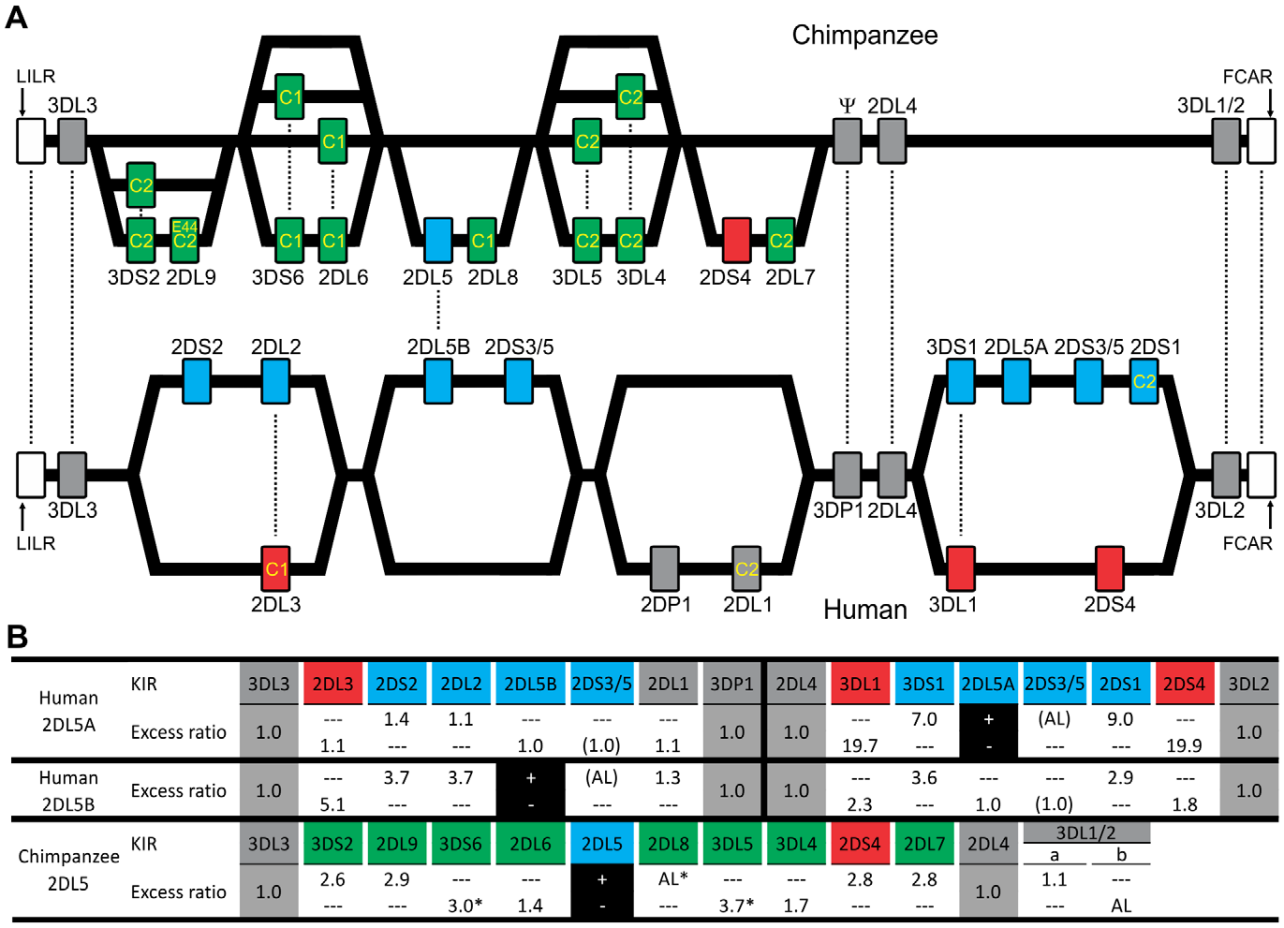
Human and chimpanzee *KIR* haplotypes differ in their organization and generation of diversity. (A) Diversity in chimpanzee arises from the variable recombination of seven units in the centromeric region, whereas a similar number of human gene-content motifs is divided between the centromeric and telomeric regions. C1 or C2 specificity for each lineage III KIR is shown. Dotted lines indicate orthologs (between species) or alleles (within species). Ψ, *KIR* pseudogene. (A–B) *KIR* associated with *A* haplotypes are red; *KIR* associated with *B* haplotypes are blue. Chimpanzee *KIR* having no human strict ortholog are colored green. (B) Linkage to *KIR2DL5* in human and chimpanzee. For each *KIR* an association ratio with 2DL5^+/−^ haplotypes is given (for example *KIR3DS1* is seven times more common on *2DL5A* ^+^ haplotypes than on *2DL5A* ^−^ haplotypes); *2DS3/5* ratios are given in parenthesis to reflect an assumption (see Materials and Methods for details). Black boxes, reference gene for the linkage analysis (+, presence; −, absence). Ratios for framework *KIR* are shaded in gray. AL, absolute linkage. Linkage disequilibrium was assessed in chimpanzee and (*) indicates significance (p<0.001).

From sequence analysis of cDNA [23] and three *KIR* haplotypes (Figure 1A), we defined 13 chimpanzee *KIR* genes. Typing a panel of 39 individuals identified 16 genotypes (Figure 1B), for which the component *KIR* haplotypes were deduced (Figure 1C). Both in number and gene content difference, chimpanzee *KIR* genotypes are within the human range (Figure 1D). Common to the human and chimpanzee *KIR* loci are three conserved, framework regions separated by centromeric and telomeric intervals of variable gene content [25]. Whereas the human variable *KIR* genes are evenly distributed between the two intervals, their chimpanzee counterparts are restricted to the centromeric interval, leaving the telomeric interval both short and empty (Figure 2A).

Although human and chimpanzee each have ten variable *KIR* genes, only *2DL5* and *2DS4* are held in common. These two genes distinguish the human group *A* and *B KIR* haplotypes, a difference correlating with a wide range of clinical effects [10]. *KIR2DL5* is the only inhibitory *KIR* restricted to *B* haplotypes, *2DS4* the only activating *KIR* of *A* haplotypes (Figure 2A and 2B). Whereas human *2DS4* is restricted to the telomeric region and present on ∼50% of *KIR* haplotypes, chimpanzee *2DS4* is restricted to the centromeric region (Figure 2B) and present on only 8% of haplotypes (Figure 1C). Also varying between species are the location of *2DL5* and its linkage disequilibrium (LD). Restricted to the centromeric region, chimpanzee *2DL5* has absolute LD with inhibitory *KIR2DL8*, whereas human *2DL5* has absolute LD with activating *KIR2DS3/S5* and is alternatively found in the centromeric region, the telomeric region, or both (Figure 2A and 2B). Thus human-specific evolution of the *KIR* locus involved ‘colonization’ of the telomeric region of the *KIR* locus, with assembly of *A* and *B* haplotype gene-content motifs around the *2DS4* and *2DL5* genes, respectively. Consequently, human *KIR* haplotypes all have *2DS4* and/or *2DL5*, while almost half the chimpanzee haplotypes (44%; arithmetic sum of the individual frequencies of haplotypes 1, 6, 9 and 15) lack both of them.

### Chimpanzee lineage III KIR are more numerous and functional MHC-C receptors than their human counterparts

The ten variable chimpanzee *KIR* form five pairs within the centromeric region (Figure 1C and Figure 2A). As shown in Figure 1B, Pairs 2, 3 and 4 at high phenotype frequency are flanked on the centromeric side by Pair 1 of intermediate frequency and on the telomeric side by Pair 5 of low frequency. Because Pairs 1, 3, and 5 have absolute or very high LD (Figure 1C), gene-content diversity of chimpanzee *KIR* haplotypes derives from asymmetric recombination between seven units, these three high LD pairs and the individual genes of Pairs 2 and 4. In humans, a similar number of units is divided between the centromeric and telomeric regions and separated by a unique and repetitive sequence that facilitates symmetric recombination [26] (Figure 2A). Thus recombination of centromeric and telomeric gene-content motifs, a major component of human *KIR* haplotype diversification, is not a significant feature of the chimpanzee system.

Eight variable chimpanzee lineage III *KIR* have no human equivalents and represent lineage III *KIR* encoding high-avidity receptors for the C1 and C2 epitopes of MHC-C. Two inhibitory and one activating KIR are C1-specific, four inhibitory and one activating KIR are C2-specific [27], [28]. Contrasting with this battery of potent MHC-C receptors is the set of six variable human lineage III KIR without chimpanzee equivalents. These comprise high-avidity inhibitory receptors for C1 (2DL2/3) and C2 (2DL1), a C2 receptor with lower avidity (2DS1) and three KIR with no detectable binding to HLA class I (2DS2, 2DS3, and 2DS5) [29]. The lineage III KIR expansion associated with hominid evolution and ‘first’ detected in the orangutan [30] was further elaborated in chimpanzee and human, but in distinctive ways. Whereas the chimpanzee retains a diversity of strong inhibitory and activating MHC-C receptors, the human system is characterized by a reduced number of inhibitory receptors and a variety of activating receptors with loss of function [29].

### Recombination of ligand-binding and signaling domains diversifies chimpanzee *KIR* function

**Figure 3:**
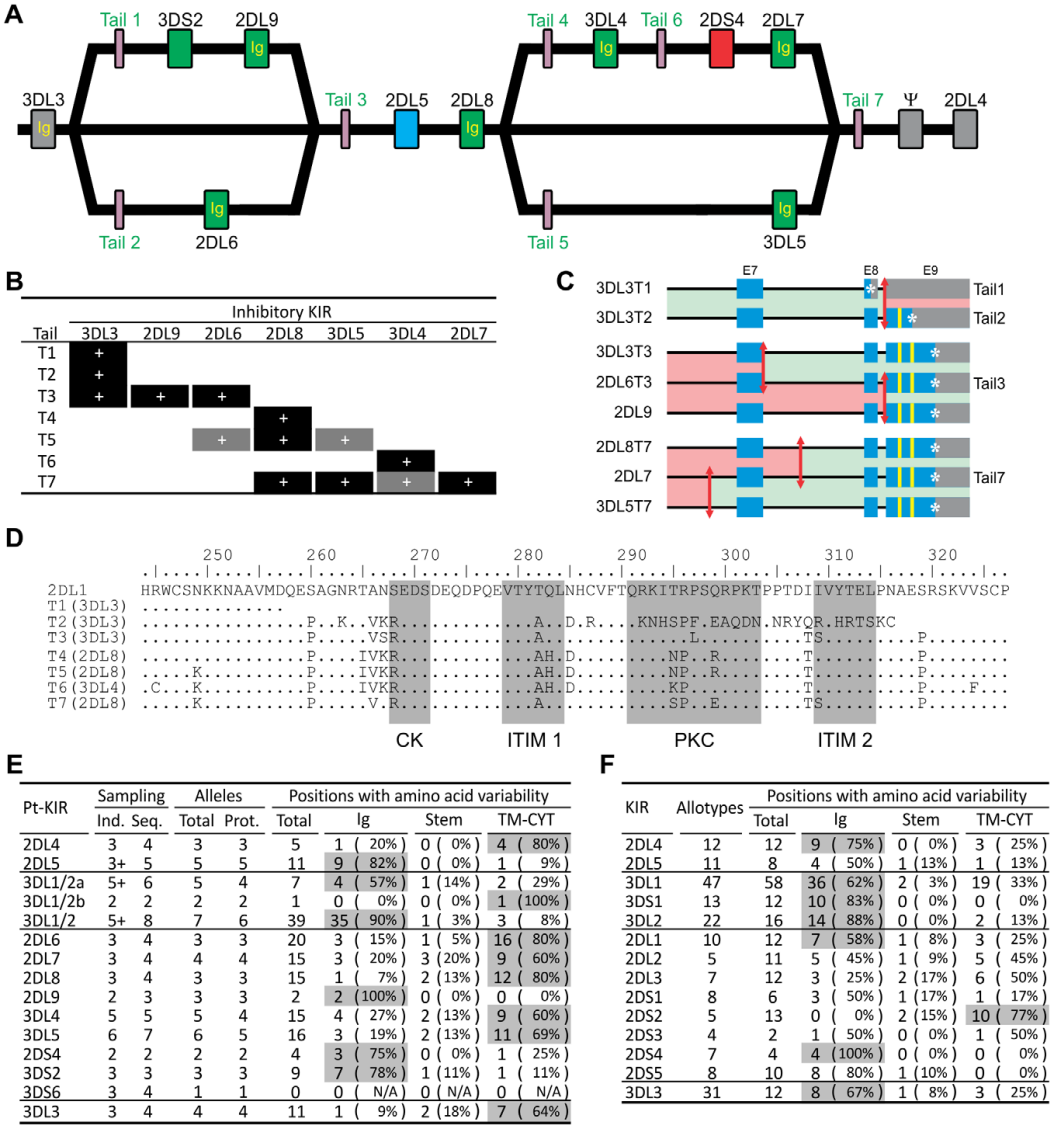
Reassorting inhibitory signaling and ligand-binding functions contributes to chimpanzee but not human *KIR* allelic diversity. (A) Structural relationships between the three chimpanzee haplotypes. ‘Ig’ and ‘Tail’ refer to the exons encoding the immunoglobulin-like domains and the cytoplasmic tail, respectively. Colors for the genes are as in Figure 2. (B) Chimpanzee inhibitory KIR associate with different cytoplasmic tails on different haplotypes. Black boxes, combinations seen in genomic sequences; gray boxes, additional combinations seen in cDNA sequences (see Figure S13). (C) Mapping of the recombination points for the *KIR* genes with tails 1–3 and 7. Red arrows represent recombination breakpoints. Regions colored in green are equivalent (allelic) in the two genes. Blue denotes coding regions, gray the 3′UTR region, and yellow the immunoreceptor tyrosine-based inhibition motifs (ITIM). *, stop codon. (D) Sequence diversity of the seven groups of chimpanzee cytoplasmic tails. One sequence for each group is displayed. Human 2DL1 is used as the reference. Highlighted in gray are the two ITIM, the protein kinase C motif (PKC) and the casein kinase motif (CK) [38]. (E–F) Chimpanzee (E) and human (F) *KIR* polymorphism. Domains contributing >50% of the variability are shaded in gray. Ind., estimate of the number of individuals sampled (‘+’, minimum number). Seq., number of nucleotide sequences. Prot., number of amino acid sequences. Chimpanzee sequences are given in Figure S14. Human *KIR* polymorphism was obtained from the IPD-KIR database [32].

The *3DL3* and *2DL8* genes are represented on each of the sequenced *KIR* haplotypes by alleles that encode the same extracellular domains but different cytoplasmic tails (Figure 3A). In reciprocal manner, the same cytoplasmic tail can be associated with different extracellular domains. Thus, the T3 tail is alternatively associated with the extracellular domains of 3DL3, 2DL9 and 2DL6, as is the T7 tail with the 2DL8, 3DL5 and 2DL7 extracellular domains (Figure 3A and 3B). These chimeric forms are the products of intergenic recombination that brought together the extracellular domains of one KIR with the signaling domain of another. The effect of this mechanism is to generate receptors with altered inhibitory signaling function (Figure 3C and 3D). For example, of the three tails associated with 3DL3, T1 has no immunoreceptor tyrosine-based inhibitory motif (ITIM), T2 has one, and T3 has two. The extent this occurred among the three haplotypes sequenced, points to the prevalence of the phenomenon and its significant contribution to the functional diversity of inhibitory chimpanzee KIR. Consistent with this thesis, sequence variability in the chimpanzee lineage III inhibitory KIR concentrates in the signaling domain (Figure 3E). However, that is not the case for human lineage III KIR, for which tail-swapping has principally served to convert inhibitory to activating receptors [31], and allotypic variability more evenly distributes between ligand-binding and signaling domains (Figure 3F). Consequently, chimpanzee 3DL3 and inhibitory lineage III KIR display more allelic variability than their human counterparts (Figure 3E and 3F). Particularly striking is 3DL3, for which four chimpanzee allotypes have 11 variable positions, compared to 12 in 31 human allotypes [32].

### Divergent sublineages of lineage III *KIR* encode human and chimpanzee MHC-C receptors

**Figure 4:**
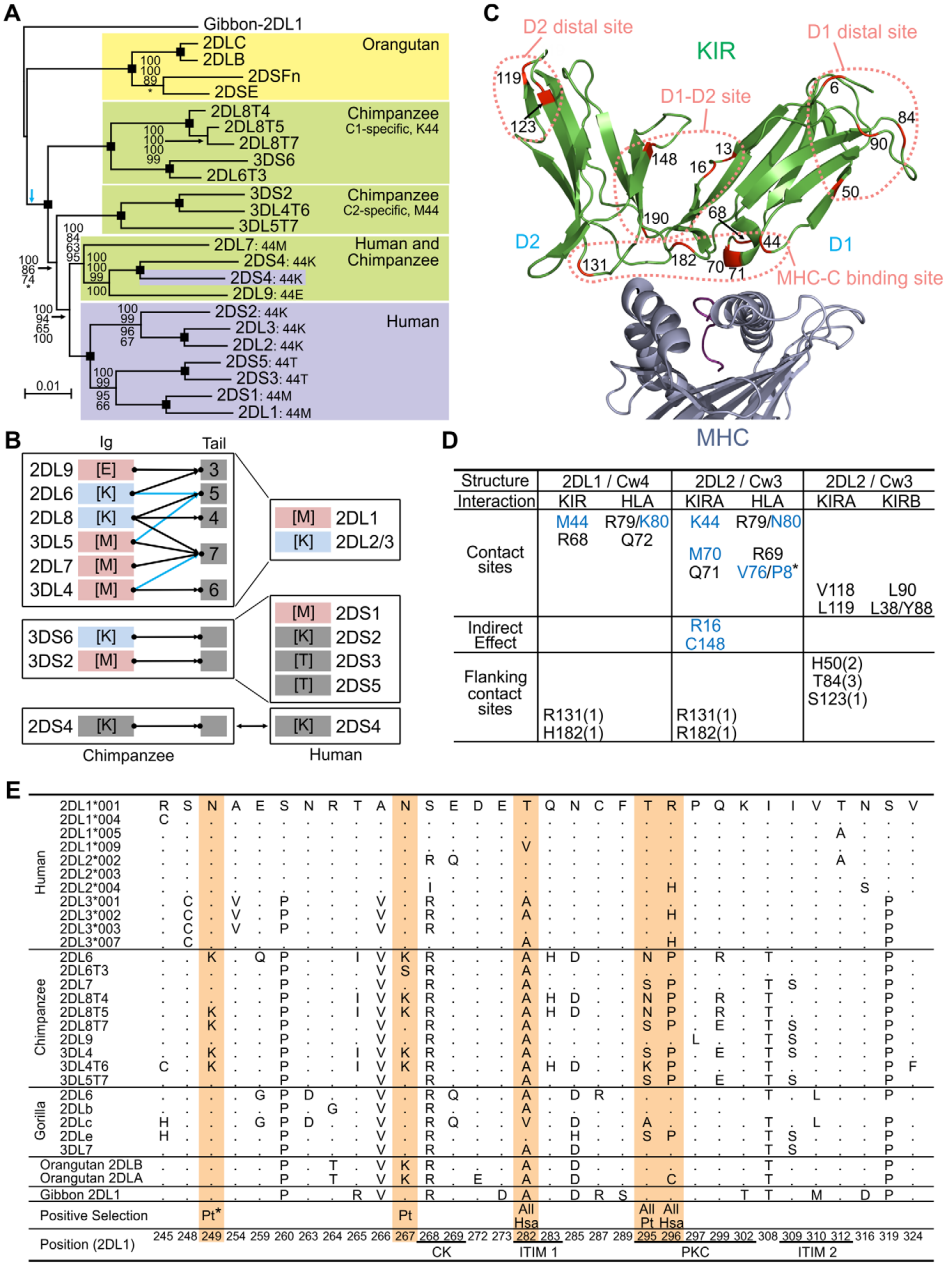
Natural selection differentially diversified human and chimpanzee lineage III *KIR*. (A) Phylogenetic analysis of lineage III *KIR* genomic sequences. The Bayesian tree topology is displayed and rooted with the gibbon sequence (blue arrow: midpoint root). Support is indicated for each node: Bayesian, maximum-likelihood, parsimony and neighbor-joining (top to bottom). Black squares: support is 100 with the four methods. *, support<50. (B) Lineage III KIR content in human and chimpanzee. Chimpanzee KIR are represented as a combination of Ig and tails with MHC specificity-determining residue 44 in the D1 domain in brackets. KIR are colored blue (MHC-C1 specific) or pink (MHC-C2 specific). Arrows indicates genomic (black) or cDNA (blue) Ig-Tail combinations (Figure 3B). (C) D1 and D2 positively selected positions (M8 p>0.95, Figure 5) are marked in red in the KIR2DL2-HLA-Cw3 three-dimensional structure (PDB file 1EFX [34] represented). (D) KIR-MHC and KIR-KIR interactions for the selected residues: KIR2DL1-HLA-Cw4 [33] (left); KIR2DL2-HLA-Cw3 [34] (center), and KIRA-KIRB (right). Mutations at residues colored blue can disrupt KIR-HLA interaction [8], [46]. In parenthesis is the number of residues to the nearest contact site. *, P8 is the eighth residue in the peptide bound by HLA-C. (E) Diversity in the cytoplasmic tails of inhibitory lineage III KIR. Positions with amino acid variation are represented (reference sequence is KIR2DL1). Orange highlight denotes positively selected position (M8 p>0.95) in one or more of three datasets comprising hominoid (‘All’), chimpanzee (‘Pt’) or human (‘Hsa’) sequences. Positions underlined correspond to the functional sites described in Figure 3D. *, only detected in the complete dataset (Figure 5).

The genomic regions containing the lineage I, II, and V *KIR* genes are shared by human and chimpanzee *KIR* haplotypes (Figure 2A, Figure S1, and Figure S2). In contrast, the regions containing the lineage III *KIR* genes have diverged to form four sublineages (Figure 4A). Of these, two are chimpanzee-specific, one is human-specific and one is shared. The two chimpanzee-specific sublineages correspond precisely to C1- and C2-specific KIR. Functionally, these sublineages were lost during human evolution (a non-functional remnant is *KIR3DP1*), being replaced by the human-specific sublineage that includes both C1- and C2-specific receptors. The shared sublineage includes additional chimpanzee inhibitory C2 receptors and 2DS4. The differences in the MHC-C system of receptors in human and chimpanzee are seen to be mainly the result of human-specific evolution. These differences alter basic functional characteristics such as the number and avidity of receptors (Figure 4B), suggesting that natural selection played distinctive roles in the evolution of human and chimpanzee lineage III KIR.

### Ligand-binding, KIR-KIR interaction, and signaling function were all subject to natural selection

**Figure 5:**
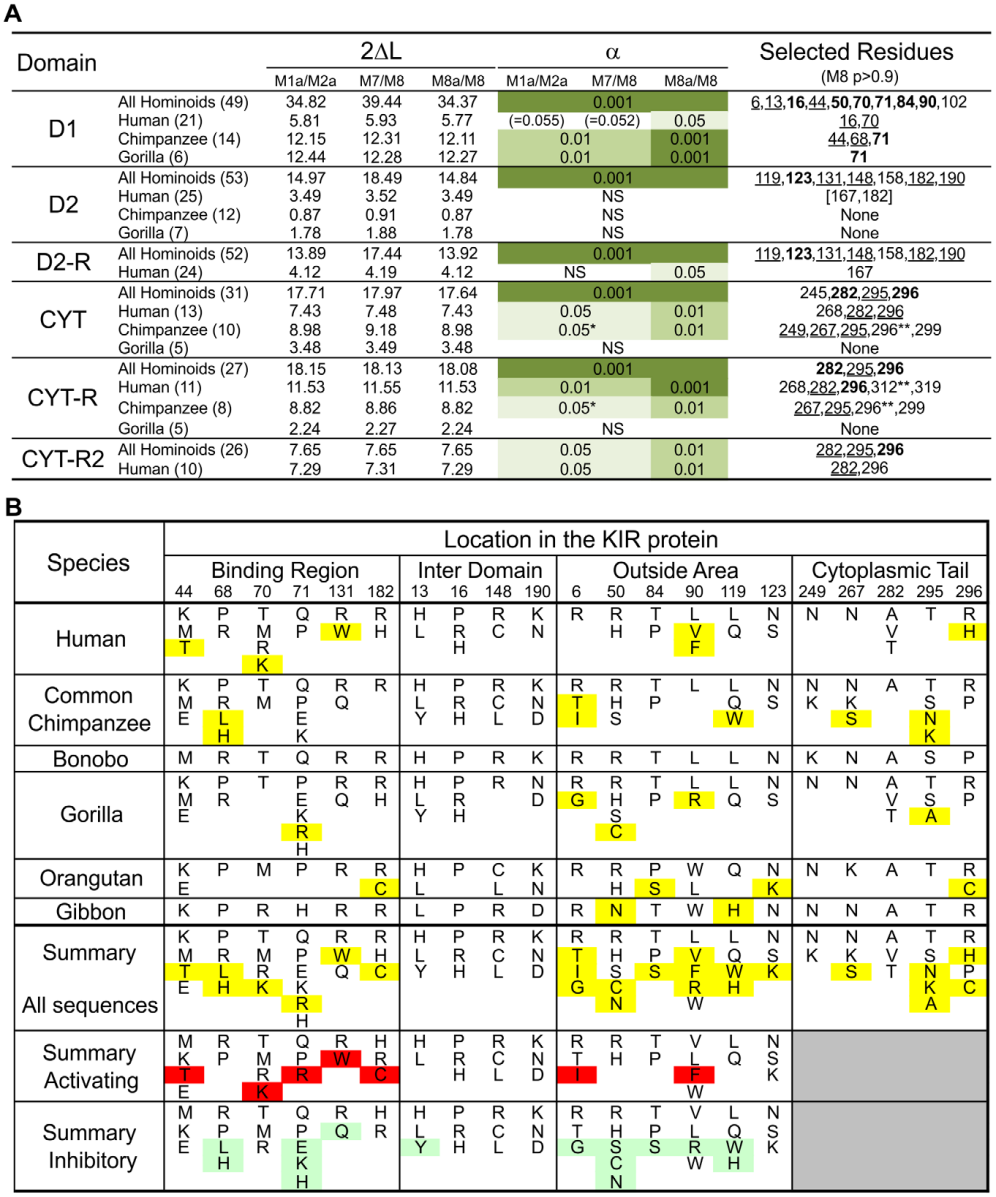
Analysis of positive selection in the lineage III KIR. (A) Likelihood ratio tests and detection of the D1, D2 and cytoplasmic tail (CYT) positions positively selected. Analysis was performed for all the hominoid sequences together and with just the human or chimpanzee or gorilla sequences; the number of sequences in each analysis is indicated in parentheses. For the cytoplasmic tail analyses all the sequences with an early termination (short tail KIR) were excluded. In addition, because of the risk of recombination between exon 7 and exon 8 (see Figure 3C) a reduced dataset was also created (CYT-R) where the nine amino acids encoded by exon 7 were discarded and the Pt-KIR2DL9 amino acids encoded by exon 8 were masked. Similarly, because the cytoplasmic tail of KIR2DL3*007 and the D2 domain of KIR2DL1*004 showed evidence of gene conversion, additional analyses were performed where these sequences were discarded: datasets CYT-R2 and D2-R, respectively. 2ΔL, two times the difference in likelihood between the models allowing for positive selection (M2a and M8) and the models that do not (null models: M1a, M7 and M8a). The significance level (α) is indicated when the null models were significantly rejected. NS, not significant. *: α∼0.01. When significant evidence of positive diversifying selection was obtained, the residues detected with model M8 were listed (underlined residues: p>0.95; boldened residues: p>0.99; **: p∼0.90). (B) Residues observed at the positively selected positions in the D1, D2 and CYT domains. Amino acids unique to one species are highlighted in yellow. In the summary section, amino acids only found on activating KIR are highlighted in red while those only found on inhibitory KIR are highlighted in green.

Evidence of positive diversifying selection was obtained for 16 positions in the ligand-binding domains of hominoid lineage III KIR (Figure 4C and Figure 5A). These positions cluster at four sites on the molecular surface: the MHC-C binding site, a site near the hinge where D1 and D2 interact (D1–D2 site), a site on D1 away from the interactions with D2 and MHC-C (D1 distal site), and a similarly distal site on D2 (D2 distal site).

Crystallography defined the MHC-C binding site [33], [34], and mutagenesis identified D1–D2 sites that modulate avidity for MHC-C (Figure 4D). In both sites there was species-specific selection. Residues 44, 68 and 71 were subject to selection in chimpanzee, compared to residues 16 and 70 in humans. At positions 44, 68, and 71, chimpanzee inhibitory receptors have residues absent from their human counterparts, while the human evolution of low-avidity activating KIR introduced unique human-specific residues at positions 44, 70 and 131 (Figure 5B). Thus the independent evolution of human and chimpanzee lineage III KIR involved fixation, under natural selection, of species-specific residues at sites affecting binding of MHC class I ligands.

Five of the 16 selected positions in D1 and D2 are implicated in the intermolecular KIR-KIR interaction observed in the KIR2DL2-HLA-Cw3 structure [34]: positions 119 and 90 are direct contact sites and residues 50, 84 and 123 are only 1–3 residues away from a contact site (Figure 4D). This distribution points to such KIR-KIR interactions being physiologically relevant, possibly contributing to the aggregation of receptors and ligands observed in the synapse between an NK cell and its target cell [35].

In the cytoplasmic tail, positive diversifying selection targeted three positions (282, 295, and 296) (Figure 4E and Figure 5A). Position 282 is in the first ITIM that initiates inhibitory KIR signals by recruiting the tyrosine phosphatase SHP-2 [36]. Favoring such recruitment is alanine 282 [37], fixed in chimpanzee but present in a minority of human lineage III KIR. Residues 295 and 296 are part of the protein kinase C (PKC) site, comprising residues 291–303. Phosphorylation of serine 298 attenuates inhibitory function [38] and is favored by arginine or lysine residues within the PKC site [39], [40]. In chimpanzee, but not human KIR, positions 295, 296 and 299 (also selected in chimpanzee KIR: p>0.9, Figure 5A) have residue combinations that variably involve arginine and lysine, indicating a modulation of affinity for PKC.

### Recurrent loss and gain of MHC-B carrying the C1 epitope recognized by lineage III KIR

**Figure 6:**
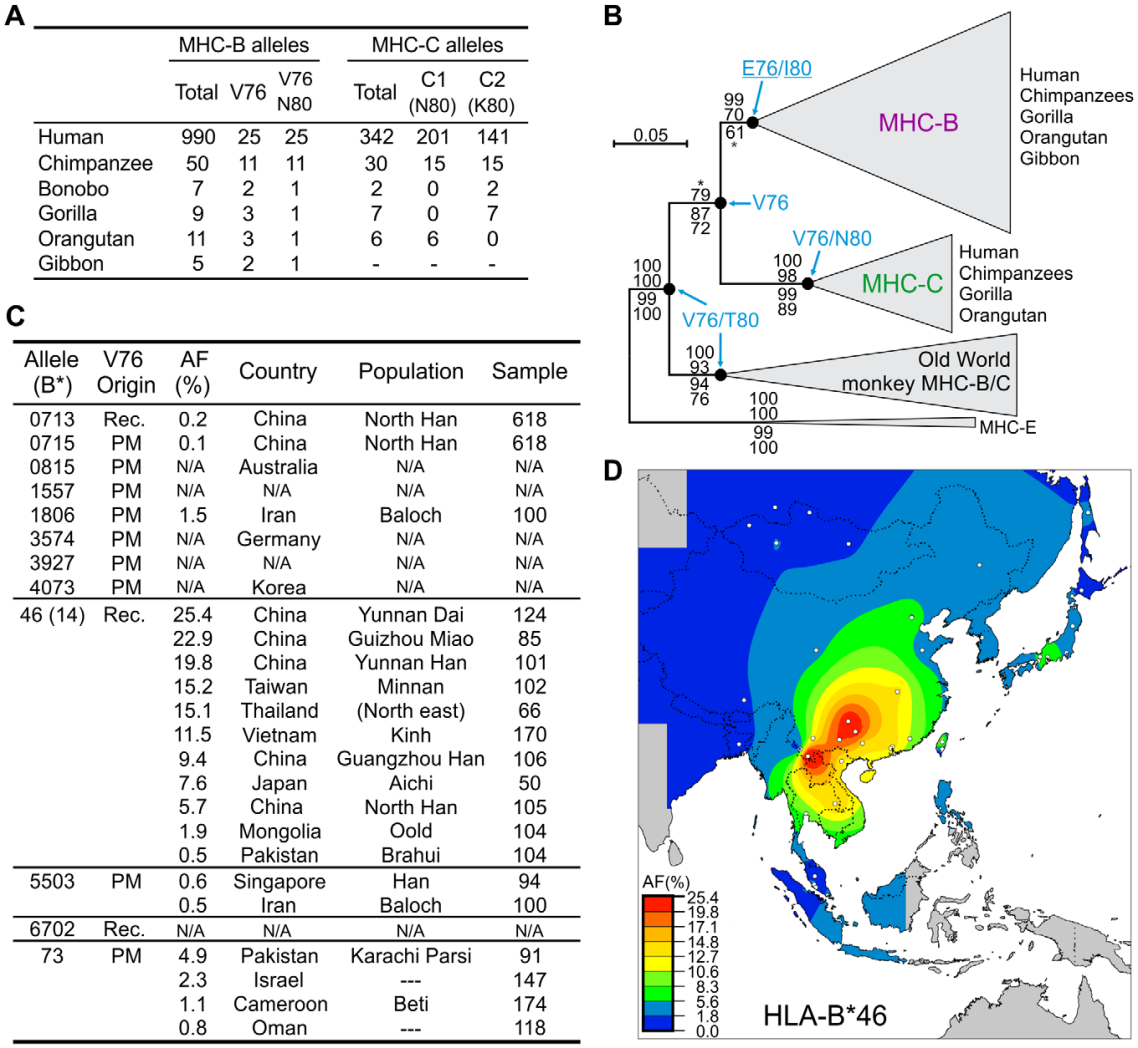
Emergence of MHC-C affected the functional interactions between MHC-B and lineage III KIR. (A) Distribution of the hominoid MHC-B and -C residues that affect interaction with lineage III KIR. (B) Phylogenetic analysis of the *MHC-B* and *-C* sequences (α1–α3 domains). The maximum-likelihood tree topology was used for the display with PAML M0 branch lengths. The tree was rooted with *MHC-E* and support is given at nodes: Bayesian, maximum-likelihood, neighbor-joining and parsimony (top to bottom). *, support <95 (Bayesian) or <50 (parsimony). Ancestral residues were reconstructed for positions 76 and 80 and are given for five nodes where model M0 p>0.95 (residues underlined were obtained with model M2a; see Materials and Methods). Groups of sequences were collapsed to simplify display; Old World monkey *MHC-B/C* have similarities to both hominoid *MHC-B* and *-C*. (C) HLA-B allotypes with V76. AF, allele frequency. Populations with HLA-B*73 AF≥0.8% are listed; for HLA-B*46, populations were selected to represent the range of AF. Rec., recombination with *HLA-C*. PM, point mutation. N/A, not available. (D) Distribution of HLA-B*46 AF in Southeast Asia. White dots represent sample points.

Lineage III KIR recognize the C1 and C2 epitopes of MHC class I. C2 depends upon valine 76 (V76) and lysine 80 (K80), a motif restricted to a subset of MHC-C allotypes. C1 depends upon V76 and asparagine 80 (N80), a motif present in subsets of MHC-C and -B allotypes; 22% of chimpanzee MHC-B allotypes have C1, compared to only 2.5% for HLA-B (Figure 6A). Ancestral sequence reconstruction indicates that the last common ancestor of MHC-B and -C had V76, which remained fixed during MHC-C evolution, but was replaced by glutamic acid (E76) during MHC-B evolution (Figure 6B). The V76 observed in modern MHC-B allotypes arose de novo, by reversion from E76, with at least fifteen such events having occurred in hominoids (Figure S3).

Of 12 distinct V76-containing HLA-B (Figure 6C), 11 emerged in modern human populations, either by point substitution (N = 8) or recombination (N = 3). Exceptional is the highly divergent HLA-B73, which combines features of both MHC-C and chimpanzee and gorilla MHC-B [41]. Eight point mutations independently introduced V76 and the C1 epitope into a range of HLA-B allotypes having N80. In contrast, V76 is never present in HLA-B allotypes having either isoleucine or threonine 80 (33% of the total), a distribution unlikely to occur by chance (α = 0.05). Likewise all 11 chimpanzee MHC-B allotypes with V76 have N80 (Figure 6A). Thus selection has favored MHC-B variants having C1 (V76-N80) and eliminated variants having V76-I80 or V76-T80. A possible mechanism for the latter effect is that HLA-B allotypes having I80 and T80 carry the Bw4 epitope recognized by KIR3DL1 [42], [43], and that V76 perturbs this interaction while failing to introduce either C1 or C2.

### HLA-B46, a potent KIR ligand, has undergone a selective sweep in Southeast Asia

Recombination events with HLA-C introduced V76 into three N80 HLA-B allotypes (Figure 6C). Of these HLA-B*46 rose to high frequency in Southeast Asia (Figure 6C), where it originated (Figure 6D) [44] following the arrival of modern humans ∼55–65,000 years ago [45]. During this selective sweep B*46 further diversified by point mutation to give 14 low-frequency subtypes (Figure 6C). The B*46 frequencies of 25.4% and 22.9% in the Yunnan Dai and Guizhou Miao, respectively, are the highest for any *HLA-B* allele in populations exceeding one million individuals, being of a magnitude typical for small, isolated or bottlenecked populations (Figure S4).

**Figure 7:**
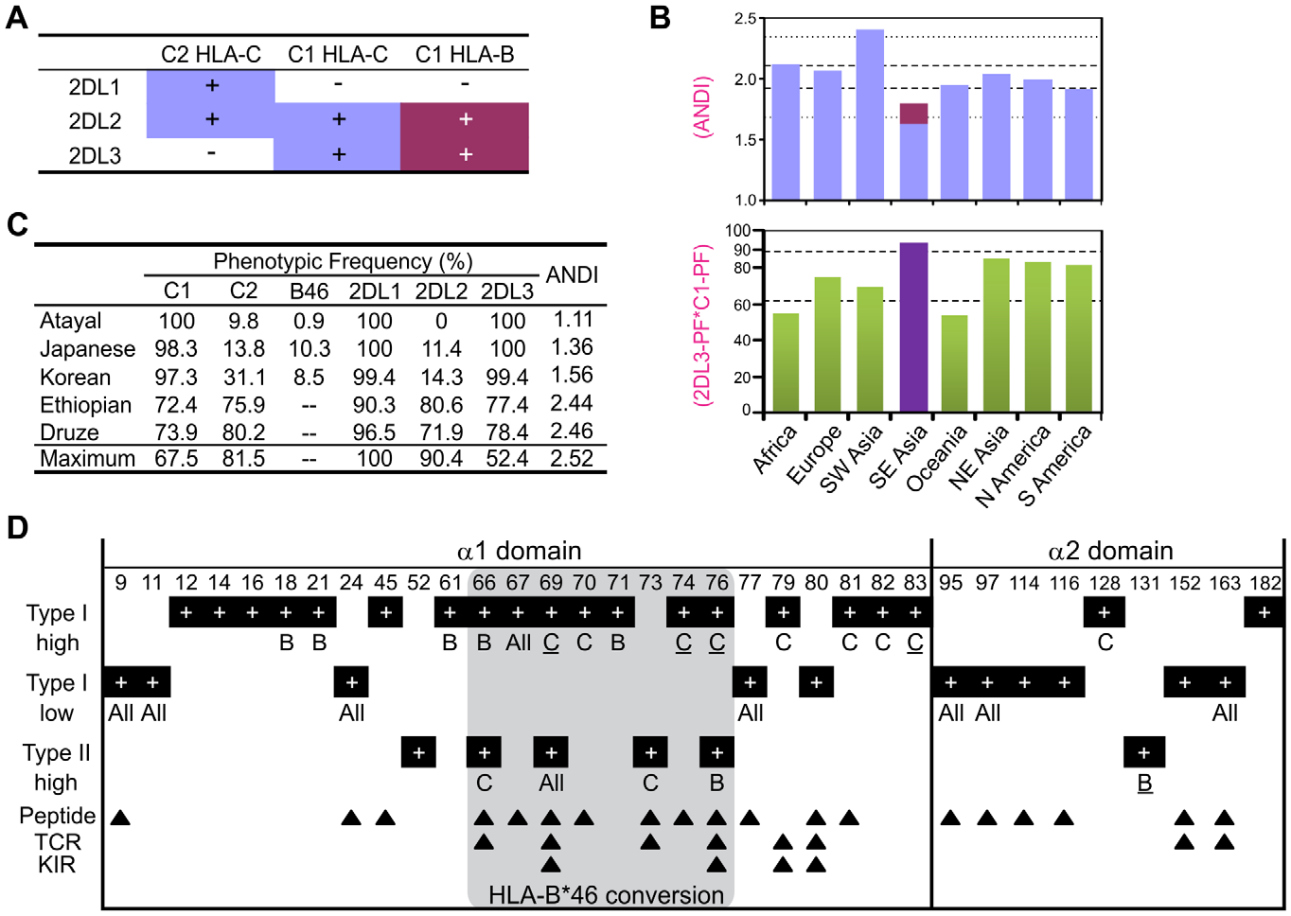
MHC-B allotypes that acquire lineage III KIR-binding increase NK cell effector capacity. (A) Summary of the KIR2DL/HLA-B (magenta) and KIR2DL/HLA-C (blue) interactions. (B) Average number of distinct KIR2DL-HLA interactions (ANDI) (top) and 2DL3_PF_*C1_PF_ quantity (bottom; PF, phenotype frequency) in eight human population groups (see Materials and Methods; individual populations are in Figure S5). Area between the gapped lines is the 25–75 percentile range; area between the dotted lines (top part) is the non-outlier range (Whisker plot with 1.5 coefficient). Colors in the top part are as defined in (A). Population group in purple (bottom part) contains populations with HLA-B*46 phenotype frequency of 8.7–27.5%. (C) KIR-HLA phenotypic frequencies for five individual populations. Maximum: maximum ANDI assuming Hardy-Weinberg equilibrium. (D) Type I and type II functional divergence between MHC-B and -C α1–α2 domains. Positions characterized in this analysis are listed at the top of the panel, and black boxes indicate in which analysis these positions were characterized. ‘high’ refers to functional-divergence analyses while ‘low’ refers to an analysis to detect low functional divergence. MHC-B specific sites (indicated by a ‘B’) are defined as divergent in the MHC-B vs. MHC-C and MHC-B vs. Old World monkey-B/C comparisons but not in the MHC-C vs. Old World monkey-B/C comparison. The same approach was used for the MHC-C specific positions (indicated by a ‘C’). ‘All’, functionally-divergent in all pairwise comparisons. Underlined residues have better support (Figure S7). The gray box indicates residues that are in the recombinant region of HLA-B*46. Arrows indicate Peptide [73], TCR [74] and KIR [33], [34] contact sites.

HLA-B*46 is a good ligand for KIR2DL2/3 [44], [46], [47] and a good educator of KIR2DL3-expressing NK cells [48]. It also gives individuals the flexibility to express up to four HLA class I isoforms bearing C1-C2 combinations (Figure 7A). From HLA and KIR frequencies, we determined the average number per individual of distinct interactions (ANDI) between KIR2DL receptors and HLA ligands. In all population groups ANDI ranged from 1.7–2.4, with a median of 2.0 (Figure 7B and Figure S5). Because of their low C2 and KIR2DL2 frequencies, Southeast Asians have the lowest ANDI worldwide, despite the significant contribution of B*46 (shown in red in Figure 7B and in Figure S5). When B*46 is excluded from the analysis, Southeast Asian ANDI values fall out of the normal range. Thus the rise of B*46 in these populations has compensated for their reduced frequency of functional ligand-receptor pairs (Figure 7C, Figure S5, and Figure S6).

The evolution of MHC-B and -C was marked by extensive type I functional divergence (site-specific rate shift; α = 9.9E-22) as well as more limited type II functional divergence (shift of cluster-specific amino acid property; α = 0.048) (Figure S7A). Of eighteen locus-specific sites detected, sixteen are in the α1 domain, nine of them (including three of the four with strongest support) within residues 66–76 of the α1 helix (Figure 7D) [49], the segment recombined in forming B*46 [50]. Type I functional divergence was greater in MHC-C (nine positions, including the four with the strongest confidence) than MHC-B (five positions). The nine C-specific positions are fixed in MHC-C but variable in MHC-B and Old World monkey MHC-B/C. Thus during evolution of MHC-C to become the major ligand for lineage III KIR, functionally favorable residues were fixed at positions throughout the α1 helix (Figure 7D). Conversely, eleven positions exhibiting low type I divergence distribute evenly between the α1 and α2 domains, all but one being highly variable peptide-binding residues.

Type II functional divergence was more limited and equally distributed between MHC-B and -C (two positions each). Notably, however, this divergence included the valine to glutamate change at position 76 in the α1 domain of MHC-B (Figure 6B and Figure 7D). Overall, functional divergence of MHC-B and -C focused on the α1 helix, while maintaining similarity in the peptide-binding site. Consequently, the localized recombination that introduced residues 66–76 from HLA-C, conferred several C-like functions and selective advantage to the recombinant B*46 allotype [44], [51].

## Discussion

Since their ancestors separated 6.5–10 million years ago, human and chimpanzee acquired major differences in *KIR* haplotype structure and gene content. These differences arose from specializations evolved on the human line. For chimpanzee *KIR* haplotypes, variable gene content is confined to the centromeric region, where ten *KIR* genes are variably found, leaving the telomeric region empty. During human evolution the telomeric region was ‘colonized’ to create a second region of gene content variability. As a consequence of this reorganization, symmetrical recombination between the centromeric and telomeric regions has evolved to be a major mechanism for diversifying *KIR* haplotypes in humans [26] but not in chimpanzees.

A second major human-specific specialization has been to fix mutations reducing the avidity of activating KIR for HLA class I, while retaining high-avidity inhibitory KIR. This process is most striking for the lineage III KIR that comprise receptors for MHC-C, the form of MHC class I molecule that became the major source of KIR ligands (the C1 and C2 epitopes) in the course of hominid evolution. Chimpanzees have eight lineage III *KIR* with no human equivalents, all of which encode high-avidity activating or inhibitory receptors for C1 or C2 [27], [28]. In contrast the six human lineage III *KIR* with no chimpanzee counterparts encode high-avidity inhibitory receptors for C1 and C2 and four activating receptors, which acquired mutations that caused three to lose avidity for HLA-C completely and one to have it reduced [29].

The major consequence of these two specializations is that humans evolved two distinctive *KIR* haplotype groups, *A* and *B,* that are under balancing selection, present in all populations, and differently associated with disease [17]. In contrast, chimpanzee *KIR* haplotypes are variations on a theme emphasizing multiple and variable high-avidity C1 and C2 receptors. The character of the *A* haplotypes is closer to that of the chimpanzee *KIR* haplotypes: they have genes encoding high-avidity inhibitory C1, C2 and Bw4 receptors and lack their attenuated activating counterparts. In contrast, *B* haplotypes carry genes for the attenuated activating receptors and distinctive variants of the inhibitory receptors. Disease associations suggest a basis for the balancing selection, in that *A* haplotypes favor successful anti-viral defense, whereas maternal *B* haplotypes favor successful reproduction [10]. Consistent with the evolutionary plasticity of viruses and other pathogens, the *A* haplotype *KIR* genes are more polymorphic than their *B* haplotype counterparts [52], as is also seen for chimpanzee *KIR* haplotypes. In this context, human-specific evolution of group *B KIR* haplotypes can be seen as an adaptation to life-threatening complications of pregnancy, such as pre-eclampsia and eclampsia, which have not affected the chimpanzee. For example, these hypertensive conditions could have arisen from imbalance between the supply and increased demands on maternal blood caused by selection to increase the size of the neonatal human brain, to double that of the chimpanzee [53].

A third human-specific specialization has been the decreasing use of MHC-B allotypes as ligands for lineage III KIR. C1 originated in a molecule resembling MHC-B, which with duplication and differentiation led to the modern MHC-B and -C [54]. Whereas 22% of chimpanzee MHC-B allotypes retain C1, only one rare HLA-B allotype, B*73, has retained C1 and the capacity to bind KIR2DL2/3 [46]. Thus the trend for much of human evolution has been for HLA-C to become the exclusive source of ligands for lineage III KIR, potentially reducing competition between NK-cell KIR and T-cell receptors, which have overlapping binding sites on HLA class I [33], [34]. In this scenario, HLA-C became more specialized in controlling NK cell functions leaving HLA-A and -B to dominate T cell responses. A remarkable reversal of this trend occurred in Southeast Asia during the last ∼55–65,000 years [45], where HLA-B*46, a recombinant allele that carries C1 and functions well as a ligand for KIR2DL2/3 [46], underwent a selective sweep to become the most frequent *HLA-B* allele. Resolution of human hepatitis C virus infection was associated with homozygosity for KIR2DL3 and its C1 ligand [55]. The potential benefit of HLA-B*46 is that it allows individuals to express three or four C1-bearing HLA-B and C allotypes. Thus the selective sweep of B*46 could have been driven by epidemic infection caused by a pathogen like the hepatitis C virus that is preferentially resisted by individuals having enhanced representation of C1 and its cognate inhibitory KIR. Interestingly, several reports describe B*46 as a risk factor for various current infectious diseases (Figure S8), illustrating the dynamic nature of these polymorphic genetic factors and the variable pressures placed on them by functions in both immune defense and reproduction.

## Materials and Methods

### Chimpanzee panel

A panel of 39 individuals was studied; 35 western chimpanzees, two eastern chimpanzees, one central chimpanzee, and one individual of unknown geographical origin. This study was approved by the Stanford University administrative panels on human subjects in medical research and laboratory animal care.

### Chimpanzee *KIR* haplotypes

Haplotypes H13 and H2 originate from the RPCI-43 BAC library (individual: Donald) while H8 belongs to the CHORI-251 BAC library (individual: Clint). The final sequence of the H13 haplotype (clone RP43-84K19) has ‘finished sequencing’ quality (see Text S1 for details). H8 is a previously undescribed haplotype (Genbank accession number: AC155174) sequenced by the Washington University Genome Sequencing Center as part of the chimpanzee genome project. H2 was reported previously [24].

### *KIR* nomenclature and lineages

*KIR* genes and alleles were named by the KIR Nomenclature Committee [56]. A curated database is available at http://www.ebi.ac.uk/ipd/kir/ [32].

*KIR* gene names reflect the structure of the molecules they encode: following ‘KIR’, the first two characters give the number of Ig-like domains in the molecule (KIR3D have three Ig-like domains for example), and the third character is either a ‘L’, ‘S’ or ‘P’ to indicate ‘Long’ (inhibitory) or ‘Short’ (activating) cytoplasmic tails, or a pseudogene, respectively. To simplify the text in this manuscript, the acronym *KIR* is sometimes omitted.

Based on phylogenetics, the Ig-like domains form three groups: D0 (most membrane distal of KIR3D), D1, and D2 (most membrane proximal). Based on domain structure and phylogenetic comparison, human KIR are seen to form four distinct lineages: KIR of the lineages III (3DL1-2) and V (3DL3) have three Ig-like domains, while KIR of the lineages I (2DL4-5) and III (2DL1-3, 2DS1-5) have two, with D0-D2 and D1-D2 configuration, respectively.

### KIR expression study and MHC-specificity

Assessment of the expression and domain structure of the KIR encoded by genes present on the three chimpanzee *KIR* haplotypes sequenced was performed using peripheral blood mononuclear cells from the two individuals whose genomic DNA was used to construct the BAC libraries RPCI-43 and CHORI-251 (results are summarized in Figure S9; see for details). Data on the MHC specificity of chimpanzee lineage III KIR are from references [27] and [28]. Changes to the nomenclature of chimpanzee *KIR* sequences are described in Text S1 and in Figure S10.

### *KIR* genomic analyses

Complete gene sequences were aligned and divided into 14 segments, as previously described [30]. Each segment was analyzed with four methods: Bayesian, maximum-likelihood, neighbor-joining and parsimony. For the lineage III *KIR* genes, a full gene analysis was performed on all fourteen segments. Additional details are in Text S1.

### *KIR* content and haplotype structures in chimpanzee

For five of the 14 *KIR* (*2DL4*, *2DL5*, *3DL1/2a* and *b*, and *3DL5*), *KIR* content was determined as previously described [23], and for the other nine *KIR* a new typing system was developed. *KIR* haplotype structures were predicted from genotype data using the HAPLO-IHP program [57], which was originally designed to reconstruct such haplotypes. Additional details are in Text S1.

### *KIR* genotype diversity and linkage disequilibrium analysis in human and chimpanzee

To compare genotype diversity in human and chimpanzee, data from human populations from Africa [58], Europe [59], and Japan [60] were used. Because the chimpanzee panel has 26 unrelated individuals, human population data were resampled to give population sizes of n = 26. The mean, standard deviation, and 95% confidence interval for the number of genotypes in human populations were obtained from 5,000 such resamplings. Mean and standard error for the pairwise difference between genotypes were estimated using a bootstrap approach, as implemented in MEGA4 [61]. Presence/absence of each of the 14 chimpanzee *KIR* of Figure 1B was considered a single-nucleotide polymorphism and the bootstrap procedure was used to shuffle the column content (10,000 bootstrap replicates) before pairwise comparisons were performed. Data from the chimpanzee panel were then compared to data obtained from human populations using the same approach [62].

For chimpanzee *KIR*, linkage disequilibrium (LD) was investigated from the haplotype data of Figure 1C using DNASP [63]; significance was assessed using a 2-tailed Fisher's exact test. For human and chimpanzee, we also investigated *KIR* haplotype structures using *KIR2DL5* as a reference. In these analyses, gene linkage to 2DL5^+/−^ was estimated for each *KIR* as a ratio: for example *KIR3DS1* is seven times more common on *2DL5A* ^+^ haplotypes than on *2DL5A* ^−^ haplotypes. All ratios were normalized to account for the difference in frequency between *2DL5^+^* and *2DL5^−^* haplotypes. In the human locus, *KIR2DS3*/5 can occupy two different genomic locations [64], [65] and linkage between *2DL5* (*A* or *B*) and *2DS3/5* was assumed to be absolute, as supported by currently available haplotype sequences. Gene linkage to *2DL5* in human was assessed from Caucasian individuals [66].

### Selection analysis

dN/dS (ω) ratios were estimated by maximum-likelihood using PAML4 [67]. Three sets of likelihood ratio tests were conducted to compare null models that do not allow ω>1 with models that do. Significance was assessed by comparing twice the difference in likelihood between the models (2ΔL) to a χ2 distribution with one or two degrees of freedom. Codons with ω>1 were identified using the Bayes Empirical Bayes approach; see Text S1 for details.

### MHC-B and -C phylogenetic analysis

A representative set of *MHC-B* and *-C* sequences was gathered and phylogenetic analyses conducted using the approach described for the *KIR* genomic analysis (see Text S1). Ancestral sequence were reconstructed with CODEML of the PAML4 software package [67], using the marginal reconstruction approach (see Text S1 for details).

### Frequency and distribution of MHC-B/C allotypes

Data for the distribution of the MHC-B and -C residues affecting interaction with lineage III KIR in hominoids were compiled using IMGT-HLA and IPD-MHC sequences [32], [68]. In addition, a gorilla MHC-B allotype bearing C1 was identified from analysis of the gorilla shotgun sequences available at the NCBI Trace Archive website (http://www.ncbi.nlm.nih.gov/Traces/home/), and generated by the Sanger Center as part of the gorilla genome project.

Allele frequencies for C1-bearing HLA-B allotypes are from the Allele Frequency Net Database [69]; when no population data were available, the country listed in the IMGT-HLA database [68] is given.

The B46 distribution map was generated using the GMT software package [70] and a previously developed script [71]; for this distribution, only anthropology studies were used, and data from recent migrant populations were discarded when the geographical location of the pre-migration population could not be precisely ascertained.

### Divergence analysis

Type I (site-specific shift of evolutionary rate) and type II (site-specific shift of amino acid property) functional divergence analyses were performed with DIVERGE2 [72]. For the type I functional divergence analysis a likelihood ratio test was used to test the null hypothesis that the coefficient of functional divergence equals zero: twice the difference in likelihood was compared to a χ2 distribution with one degree of freedom. For the type II functional divergence analysis significance was assessed by a two-tailed Z-test. Functional divergence-related residues were identified through the use of cutoffs (see Figure S7 for additional details).

### Average number of distinct interactions (ANDI)

Using *KIR* and *HLA* phenotypic frequencies (PF) in 33 human populations (see Figure S11 for details) we determined the average number of distinct interactions between HLA-C1/C2 and KIR2DL1-3 (sum of KIR and HLA receptor-ligand pairings) with the following formula: (2DL1_PF_*C2_PF_)+(2DL2_PF_*C2_PF_)+(2DL2_PF_*C1_PF_)+(2DL3_PF_*C1_PF_). For East Asian populations we also added the interaction between HLA-B*46 and KIR2DL2-3: (2DL2_PF_*B46_PF_)+(2DL3_PF_*B46_PF_). An alternative model where the interaction between KIR2DL2 and HLA-C2 was not taken into account was also considered but the results were similar (see Figure S12).

### Statistical testing

In addition to the statistical tests described in individual sections, a binomial distribution was used to assess the probability that the substitutions that recurrently created V76 in HLA-B allotypes always occurred on allotypes with N80 by chance, and the Pearson product-moment was used to study the correlation between *KIR* and *HLA* frequencies in human populations.

## Supporting Information

Figure S1 Phylogenetic comparison of human and chimpanzee *KIR* haplotypes. (A) Intron-exon structure of a typical *KIR* gene showing the 14 datasets used in phylogenetic analyses. (B–F) Pairwise comparison of haplotypes: *H13* (top) - *H2* (left) (B); *H8* (top) - *H2* (left) (C); *H13* (top) - *H8* (left) (D); *H13* (top) - human *A* (E) and *H13* (top) - human *B* (F). Phylogenetic analyses were performed individually for the 14 segments defined in (A) using Bayesian, maximum-likelihood (ML), neighbor-joining (NJ) and parsimony methods (Figure S2). Colored squares indicate segments equivalent in the two haplotypes and colors reflect the phylogenetic support (Bayesian: posterior probability (PP), other methods: bootstrap proportion, BP): dark green squares have PP>95 and BP ≥80; light green squares are supported by three of the four methods (PP ≥90, BP ≥50) and orange squares by two of the four methods (PP ≥90, BP ≥50). Red squares indicate a segment with no equivalent in the other haplotype. Yellow squares: lack of resolution or trans-species polymorphism (chimpanzee alleles are mixed with orthologs from different species) (panels B–D) or phylogenetic group contains at least three sequences (two from one species and one from the other species) and the relationships between these sequences are not resolved (panels E–F). Tan squares: unresolved relationships between three chimpanzee sequences. Blue lines indicate conserved segments between the two haplotypes. Colors around the *KIR* gene names indicate the lineages: I (green), II (orange), III (purple) and V (red). (E–F) Orange: human and chimpanzee sequences are mixed with orthologs from other species.
(0.05 MB PDF)

Figure S2 Phylogenetic analysis of the 14 genomic segments used to compare *KIR* haplotypes. The phylogenetic reconstruction was performed on each of the 14 segments described in Figure S1A using Bayesian, ML, NJ and parsimony approaches. The Bayesian tree topology was used for the display (with a midpoint rooting) and the support with the four methods indicated for all the nodes (from top to bottom: Bayesian, ML, NJ and parsimony). Black circles at nodes indicate a strong phylogenetic support: posterior probability (PP) >95 in the Bayesian analysis and bootstrap proportion (BP) ≥80 with the other three methods. The node support was omitted for the nodes not supported by at least two methods (PP ≥80 and BP ≥50). *: PP<80 or BP<50.
(0.11 MB PDF)

Figure S3 Emergence of MHC-B allotypes with V76. This phylogenetic tree represents the MHC-B subtree of the tree presented in Figure 6B with branch lengths estimated using the PAML codon model M2a. The names of allotypes with both V76 and N80 are blue while the names of allotypes with V76 but not N80 are green. Boxes along branches indicate amino acid changes at position 76 in the α 1 domain: from glutamic acid to valine (blue) or from valine to glutamic acid (red) (dark colors: p of change>0.95; light colors: p<0.95). At nodes, boxes indicate the phylogenetic support in the maximum likelihood analysis: yellow (BS ≥50) or green (BS ≥70). #, excluding B*3902. §, change from glycine to valine (blue box) or from glutamic acid to glycine (red box).
(0.03 MB PDF)

Figure S4 B*4601 is the only *HLA-B* allele with an allele frequency >25% in a large population. The two left-most columns list the 24 most common *HLA-B* alleles worldwide and their respective worldwide allele frequency (AF) in a study of 146 worldwide population samples [71]. Collectively these alleles represent >70% of worldwide *HLA-B* AF. Two alleles initially included (HLA-B*0704 and B*3705) were subsequently excluded as no data were available for them in the Allele Frequency Net Database [69]. For each allele we obtained the population with the highest allele frequency from the Allele Frequency Net Database [69]; only data from anthropologological studies involving at least 50 individuals were used: these populations and their AFs are listed in columns 3–4. In column 3, populations shaded in gray have a modern population size <150,000 individuals while populations shaded in dark gray have a modern population size <50,000 individuals. Columns 5–6 list for each of the 24 *HLA-B* alleles the name of the large population with the highest AF, and the AF in this population. While we set a minimum of ∼200,000 individuals for a population to be included in column 5, all the populations listed in this column have modern population sizes well in excess of 1,000,000 individuals. AF>25% are shaded in orange.
(0.02 MB PDF)

Figure S5 MHC-B allotypes that reacquire binding to lineage III KIR restore or increase NK cell effector capacity. (A) Summary of the KIR2DL/HLA-B (magenta) and KIR2DL/HLA-C (blue) interactions. (B) Average number of distinct KIR2DL-HLA interactions (ANDI) (top) and 2DL3_PF_*C1_PF_ quantity (bottom; PF, phenotype frequency) in 33 human populations. Area between the gapped lines is the 25–75 percentile range; area between the dotted lines (top part only) is the non-outlier range (Whisker plot with 1.5 coefficient). Colors in the top part are as defined in (A). Populations in purple (bottom part) have HLA-B*46_PF_ of 8.7–27.5%. SWA, Southwest Asia; OCE, Oceania; NEA, Northeast Asia; NAM, North America; SAM, South America.
(0.03 MB PDF)

Figure S6 Variability of KIR2DL2 and HLA-C2 frequencies in human populations. Amongst the components of the KIR2DL/HLA-C interactions HLA-C2 and KIR2DL2 display the widest range of phenotypic frequencies in populations (Figure 7C). HLA-C2 and KIR2DL2 frequencies also correlate with the average number of distinct KIR2DL/HLA-C interactions (A) and their combined frequency distribution (B) mimics that obtained with all KIR2DL-HLA-C interactions (Figure S5) indicating they represent the main source of HLA-C/KIR2DL variability in human populations. KIR2DL2 and HLA-C2 frequencies display a positive correlation (C–D) that amplifies the difference between populations. Indeed, five of the seven African populations are, for example, in the high range of the KIR2DL2-HLA-C2 frequencies (B) while six to eight of the ten Southeast Asian populations are in the low range of this distribution (B–D) and have KIR2DL3/HLA-C1 as their main KIR2DL-HLA-C interaction. The correlation between KIR2DL2 and HLA-C2 frequencies is the strongest amongst all KIR2DL-HLA-C1/C2 combinations (E), although the correlation between KIR2DS2 and HLA-C2 is almost equally as strong due to the strong linkage disequilibrium between KIR2DL2 and KIR2DS2. (A) Pearson product-moment correlations between the average number of distinct interactions (ANDI) and HLA-C1/C2, KIR2DL1-3 phenotypic frequencies. *, Average number of distinct interactions excluding the interactions involving HLA-B*46 and the Nasioi population (see panels C-E). (B) (2DL2_PF_*C2_PF_) quantity in 33 populations. Dark gray area represents the 25-75 percentile range. (C-D) Pearson product-moment correlations between KIR2DL2_PF_ and HLA-C2_PF_ in 33 (C) or 32 (D) populations. The red point in (C) is an outlier (Nasioi population) and was removed for the analysis in (D). (E) Pearson product-moment correlations between HLA-C1/C2 and KIR2DL1-3 phenotypic frequencies. The Nasioi population was consistently an outlier, indicating that the HLA-C1/C2 and KIR2DL1-3 frequencies in this population have evolved differently than in the other 32 populations.
(0.12 MB PDF)

Figure S7 Functional divergence between the α 1 and α 2 domains of MHC-B and MHC-C. (A) Summary of the type I and type II functional divergence analyses. θ _ML_: coefficient of type I functional divergence. θ _ΙΙ_: coefficient of type II functional divergence. Significance of the type I analysis was assessed using a likelihood ratio test (LRT): twice the difference in likelihood (‘LRT’) was compared to a χ 2 distribution with one degree of freedom. For the type II analysis a two-tailed Z-test was used to assess the significance. S.E., Standard Error. (B) Identification of the type I and type II functional divergence-related residues (defined as having a posterior probability to be functional divergence-related (p) >θ _ML/ΙΙ_+0.3 [Gu]). Group-specific residues are functional divergence-related in all three comparisons while MHC-B or -C specific positions are related to functional divergence in two of the three comparisons and display an average, or lower than average, functional divergence in the third comparison. For the type I divergence, MHC-B specific positions were defined as related to functional divergence in the MHC-B/MHC-C and MHC-B/OWM comparisons (G1: p>θ _ML_+0.3; G2: p>θ _ML_+0.5) but not in the MHC-C/OWM comparison (G1: p<θ _ML_+2S.E.; G2: p<θ _ML_-0.3). For the type II divergence, MHC-B specific positions were defined as related to functional divergence in the MHC-B/MHC-C and MHC-B/OWM comparisons (G1: p>θ _ΙΙ_+0.3; G2: p>θ _ΙΙ_+0.5) but not in the MHC-C/OWM comparison (G1: p<θ _ΙΙ_+2S.E.; G2: p = 0). The same approach was used for the MHC-C specific positions. OWM, Old World monkey. (C) Identification of residues with a low type I functional divergence (defined as having p<θ _ML_-0.3). (D-F) Results of the type I (D,F) and type II (E) functional divergence analyses. Functionally-divergent sites are listed at the bottom of each graph. MHC-B specific sites are colored blue, MHC-C specific sites are colored red. Sites that are functionally-divergent in all three comparisons are green, those with a low type-I divergence are orange. Underlined residues have a better support (G2 in panel B). Boxed residues are in the recombinant region of HLA-B*46. Y axis, posterior probability of a site to be functional divergence-related. [Gu X (2006) A simple statistical method for estimating type-II (cluster-specific) functional divergence of protein sequences. Mol Biol Evol 23: 1937-1945.]
(0.04 MB PDF)

Figure S8 Disease associations with HLA-B*46. The ‘Disease’ column on the left lists the name of the disease investigated, and for infectious diseases the name of the causal pathogen. The country where the study was conducted, the type of association (resistance ‘R’ or susceptibility ‘S’) and the reference to the study are given in the ‘Association’ column. The column ‘Significance’ gives the significance of the association between the disease and HLA-B*46 (‘c’ indicates that this probability was corrected for multiple comparisons while ‘nc’ indicates no correction for multiple comparisons). §, the significance is for a subgroup of HLA-B*46 haplotypes (listed in ‘Other HLA factors’). The column ‘Other HLA factors’ lists genetic factors also found to be associated with the disease in the same study, and genetic factors in bold displayed a more significant association than B*46. Brackets in the ‘Other HLA factors’ column designate haplotypes. #, only in males. References are: [Wang LM, Kimura A, Satoh M, Mineshita S (1999) HLA linked with leprosy in southern China: HLA-linked resistance alleles to leprosy. Int J Lepr Other Mycobact Dis 67: 403-408. Hananantachai H, Patarapotikul J, Ohashi J, Naka I, Looareesuwan S, et al. (2005) Polymorphisms of the HLA-B and HLA-DRB1 genes in Thai malaria patients. Jpn J Infect Dis 58: 25-28. Chandanayingyong D, Maranetra N, Bovornkitti S (1988) HLA antigen profiles in Thai tuberculosis patients. Asian Pac J Allergy Immunol 6: 77-80. Blackwell JM, Jamieson SE, Burgner D (2009) HLA and infectious diseases. Clin Microbiol Rev 22: 370-385. Huang X, Ling H, Mao W, Ding X, Zhou Q, et al. (2009) Association of HLA-A, B, DRB1 alleles and haplotypes with HIV-1 infection in Chongqing, China. BMC Infect Dis 9: 201. Lin M, Tseng HK, Trejaut JA, Lee HL, Loo JH, et al. (2003) Association of HLA class I with severe acute respiratory syndrome coronavirus infection. BMC Med Genet 4: 9. Yoon SK, Han JY, Pyo CW, Yang JM, Jang JW, et al. (2005) Association between human leukocytes antigen alleles and chronic hepatitis C virus infection in the Korean population. Liver Int 25: 1122-1127. Wang ML, Lai JH, Zhu Y, Zhang HB, Li C, et al. (2009) Genetic susceptibility to haemorrhagic fever with renal syndrome caused by Hantaan virus in Chinese Han population. Int J Immunogenet 36: 227-229. Hildesheim A, Apple RJ, Chen CJ, Wang SS, Cheng YJ, et al. (2002) Association of HLA class I and II alleles and extended haplotypes with nasopharyngeal carcinoma in Taiwan. J Natl Cancer Inst 94: 1780-1789. Tang M, Zeng Y, Poisson A, Marti D, Guan L, et al. (2010) Haplotype-dependent HLA susceptibility to nasopharyngeal carcinoma in a Southern Chinese population. Genes Immun 11: 334-342. Ando I, Chi HI, Nakagawa H, Otsuka F (1993) Difference in clinical features and HLA antigens between familial and non-familial vitiligo of non-segmental type. Br J Dermatol 129: 408-410. Chen WH, Chiu HC, Hseih RP (1993) Association of HLA-Bw46DR9 combination with juvenile myasthenia gravis in Chinese. J Neurol Neurosurg Psychiatry 56: 382-385. Naito S, Sasaki H, Arakawa K (1987) Japanese Graves' disease: association with HLA-Bw46. Endocrinol Jpn 34: 685-688. Onuma H, Ota M, Sugenoya A, Inoko H (1994) Association of HLA-DPB1*0501 with early-onset Graves' disease in Japanese. Hum Immunol 39: 195-201. Cavan DA, Penny MA, Jacobs KH, Kelly MA, Jenkins D, et al. (1994) The HLA association with Graves' disease is sex-specific in Hong Kong Chinese subjects. Clin Endocrinol (Oxf) 40: 63-66.]
(0.05 MB PDF)

Figure S9 Relationships between chimpanzee *KIR* cDNA and gene sequences. Results of the expression study in peripheral blood mononuclear cells are given for the ten *KIR* gene sequences with no prior cDNA equivalent. *, *Pt-KIR3DL3* is from the same lineage as *KIR3DL3*, a gene expressed at low or undetectable levels in peripheral blood NK cells.
(0.01 MB PDF)

Figure S10 Sequence and position of *Pt-KIR3DS6*. (A) Sequence of *Pt-KIR3DL6* and *3DS6* at the end of exon 7 and at the beginning of exon 8. *Pt-KIR3DL6* has a stretch of seven adenosines at the end of exon 7 that maintains the typical *KIR* reading frame for exons 8 and 9, resulting in a protein with two ITIM in the cytoplasmic tail. *Pt-KIR3DS6* has eight adenosines at the end of exon 7: this changes the typical *KIR* reading frame for exons 8 and 9, resulting in a protein with a short cytoplasmic tail with no ITIM. Nucleotides in blue represent splice sites. The adenosine in red represents the extra base pair of *Pt-KIR3DS6* comparing to *3DL6*. (B) Sequencing of exon 7 of *Pt-KIR3DL6/S6* in six individuals that typed positives for *Pt-KIR3DL6*, including Alex, an individual used in the study where *Pt-KIR3DL6* was characterized [23]. PCR1 and PCR2 represent two independent PCR amplifications. ‘7A’ and ‘8A’ refer to the number of adenosines at the end of exon 7, and represent *Pt-KIR3DL6* and *3DS6*, respectively. For each amplification and individual, several clones were sequenced, and the number of clones in each category is mentioned (for each amplification, the group with the largest number of clones is shaded in gray). (C) Schematic representation of the gene-to-gene PCR amplification used to characterize the position of *Pt-KIR3DS6*. F, forward primer; R, reverse primer. TM, exon encoding the transmembrane domain; CYT1-2, exons encoding the cytoplasmic tail. L1-2, exons encoding the leader peptide.
(0.41 MB PDF)

Figure S11 *KIR* and *HLA* frequencies used to establish the average number of distinct KIR2DL-HLA-B/C interactions (ANDI) in human populations. AFR, Africa; EUR, Europe; SWA, Southwest Asia; EAS, East Asia; OCE, Oceania; NEA, Northeast Asia; NAM, North America; SAM, South America. Numbers in parenthesis indicate the number of populations in each group. References are: [69] [Single RM, Martin MP, Gao X, Meyer D, Yeager M, et al. (2007) Global diversity and evidence for coevolution of KIR and HLA. Nat Genet 39: 1114-1119. Norman PJ, Stephens HA, Verity DH, Chandanayingyong D, Vaughan RW (2001) Distribution of natural killer cell immunoglobulin-like receptor sequences in three ethnic groups. Immunogenetics 52: 195-205. Toneva M, Lepage V, Lafay G, Dulphy N, Busson M, et al. (2001) Genomic diversity of natural killer cell receptor genes in three populations. Tissue Antigens 57: 358-362. Hoa BK, Hang NT, Kashiwase K, Ohashi J, Lien LT, et al. (2008) HLA-A, -B, -C, -DRB1 and -DQB1 alleles and haplotypes in the Kinh population in Vietnam. Tissue Antigens 71: 127-134. Whang DH, Park H, Yoon JA, Park MH (2005) Haplotype analysis of killer cell immunoglobulin-like receptor genes in 77 Korean families. Hum Immunol 66: 146-154. Lee KW, Oh DH, Lee C, Yang SY (2005) Allelic and haplotypic diversity of HLA-A, -B, -C, -DRB1, and -DQB1 genes in the Korean population. Tissue Antigens 65: 437-447. Yawata M, Yawata N, Draghi M, Little AM, Partheniou F, et al. (2006) Roles for HLA and KIR polymorphisms in natural killer cell repertoire selection and modulation of effector function. J Exp Med 203: 633-645. *, some phenotypic frequencies were estimated from allele frequencies assuming Hardy-Weinberg equilibrium.
(0.02 MB PDF)

Figure S12 Alternative model for the interactions between HLA-C and KIR2DL. Unlike the analyses presented in Figure 7, Figure S5, and Figure S6, the interaction between KIR2DL2 and HLA-C2 was not taken into account in this model. (A) Summary of the KIR2DL/HLA-B (magenta) and KIR2DL/HLA-C (blue) interactions. (B) Average number of distinct KIR2DL-HLA interactions (ANDI) in 33 human populations. Area between the gapped lines is the 25-75 percentile range; area between the dotted lines is the non-outlier range (Whisker plot with 1.5 coefficient). Colors in the top part are as defined in (A). SWA, Southwest Asia; OCE, Oceania; NEA, Northeast Asia; NAM, North America; SAM, South America. (C) Pearson product-moment correlations between the average number of distinct interactions (ANDI) and HLA-C1/C2, KIR2DL1-3 phenotypic frequencies. *, excluding the interactions involving HLA-B*46 and the Nasioi population (see Figure S6C-E). (D) KIR-HLA phenotypic frequencies for five individual populations. Maximum: maximum ANDI assuming Hardy-Weinberg equilibrium.
(0.03 MB PDF)

Figure S13 Domain-by-domain phylogenetic analysis of chimpanzee *KIR* sequences. The NJ method was used for the reconstructions and phylogenetic trees were rooted at the midpoint. For nodes, the bootstrap proportion support is given when >50. The eleven cDNA sequences described in the first study of chimpanzee KIR [23] are colored in blue. The five *KIR* lineages are delimited by boxes and are indicated with white roman letters. (A) D0 domain (or pseudoexon 3). (B) D1 domain. (C) D2 domain. (D) Stem, transmembrane and cytoplasmic domains (S/TM/CYT). (E) Full-length coding sequences (including the pseudoexon 3). White brackets indicate non-recombinant alleles. Allelic relationships were established based on the overall distance in the full-length sequence analysis and consistency in the domain-by-domain analysis (panels A-D).
(0.03 MB PDF)

Figure S14 Summary of *KIR* polymorphism in chimpanzee. Amino acid variation is listed for 12 of the 13 chimpanzee KIR for which such data are available (A-L). For each KIR, the positions of variability are displayed. The origin of each sequence is given in parenthesis: Hxx and Tx (*KIR* haplotypes), cDNA (first cDNA study [23]), cDNA2 (cDNA sequences characterized in the present study), and cDNA3 (unpublished sequences deposited in Genbank with the following accession numbers: AM279149, AM292657-63, AM396937, and AM400232-36). Position, amino acid residue in the mature protein. L, leader peptide. D0-D2, Ig-like domains. S, stem. TM, transmembrane domain. CYT, cytoplasmic tail. FS, frameshift. *, Stop codon.
(0.03 MB PDF)

Text S1 Supporting text.
(0.19 MB PDF)
