# Supplementary figures and images for: Human-Specific Evolution and Adaptation Led to Major Qualitative Differences in the Variable Receptors of Human and Chimpanzee Natural Killer Cells
Source: PLoS Genet. 2010 Nov 4;6(11):e1001192. doi: 10.1371/journal.pgen.1001192 (PMC2973822; doi:10.1371/journal.pgen.1001192)

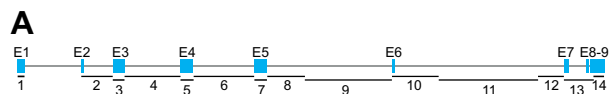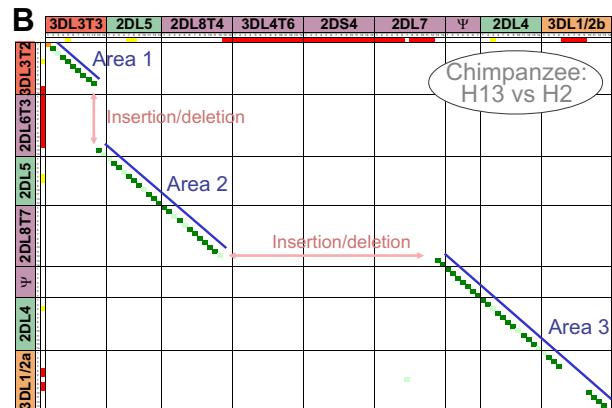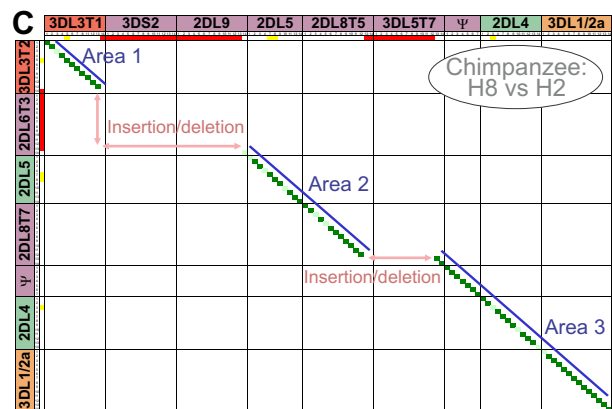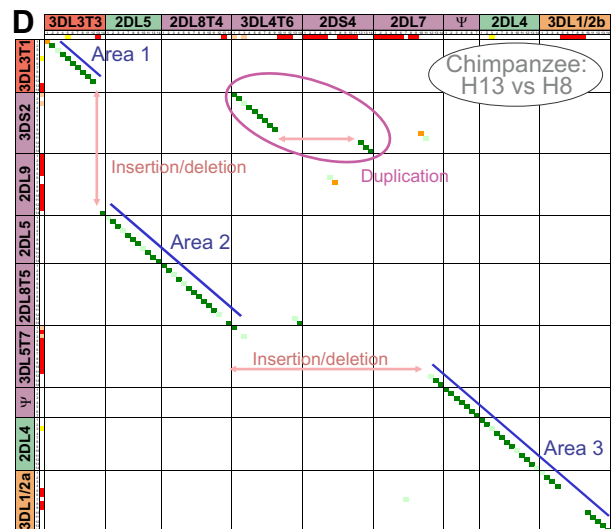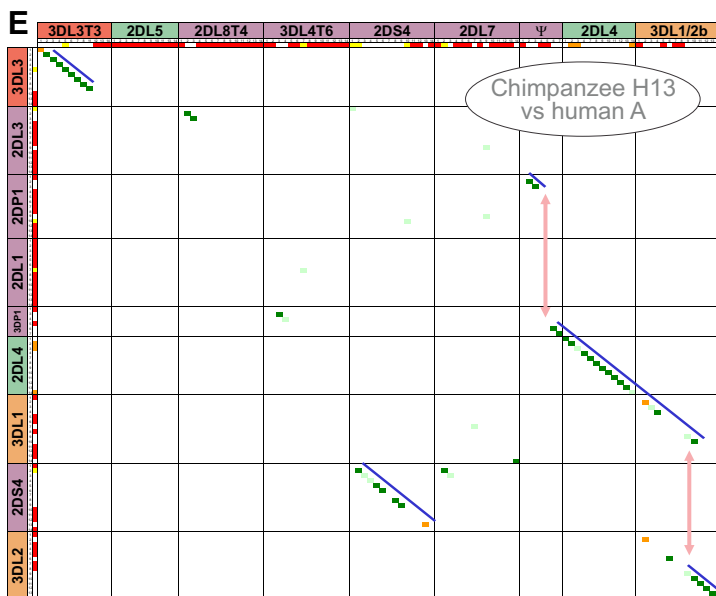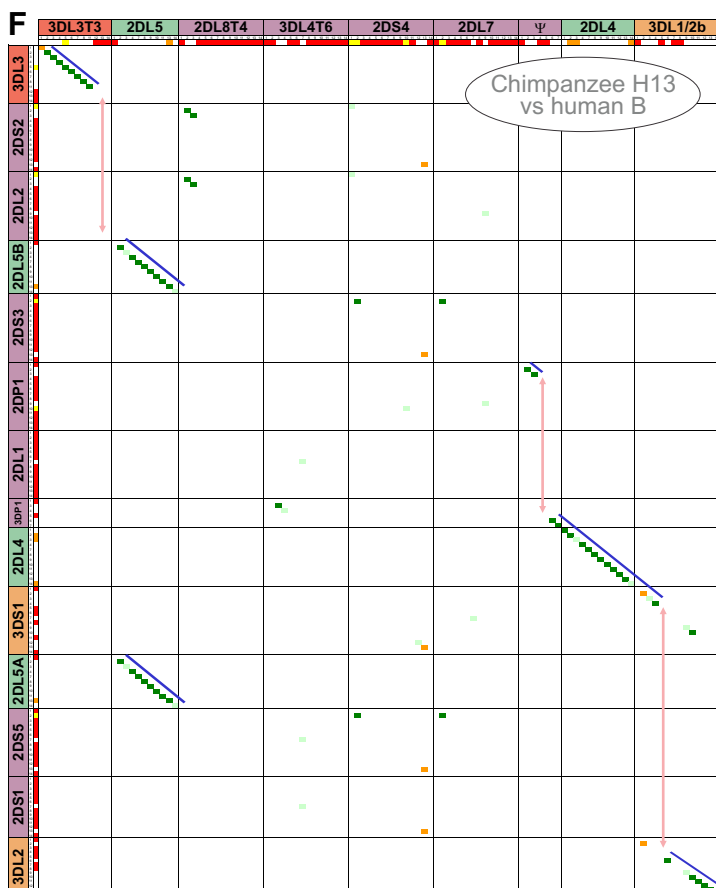

Supplement: Figure S1 — Phylogenetic comparison of human and chimpanzee KIR haplotypes. (A) Intron-exon structure of a typical KIR gene showing the 14 datasets used in phylogenetic analyses. (B–F) Pairwise comparison of haplotypes: H13 (top) - H2 (left) (B); H8 (top) - H2 (left) (C); H13 (top) - H8 (left) (D); H13 (top) - human A (E) and H13 (top) - human B (F). Phylogenetic analyses were performed individually for the 14 segments defined in (A) using Bayesian, maximum-likelihood (ML), neighbor-joining (NJ) and parsimony methods (Figure S2). Colored squares indicate segments equivalent in the two haplotypes and colors reflect the phylogenetic support (Bayesian: posterior probability (PP), other methods: bootstrap proportion, BP): dark green squares have PP>95 and BP ≥80; light green squares are supported by three of the four methods (PP ≥90, BP ≥50) and orange squares by two of the four methods (PP ≥90, BP ≥50). Red squares indicate a segment with no equivalent in the other haplotype. Yellow squares: lack of resolution or trans-species polymorphism (chimpanzee alleles are mixed with orthologs from different species) (panels B–D) or phylogenetic group contains at least three sequences (two from one species and one from the other species) and the relationships between these sequences are not resolved (panels E–F). Tan squares: unresolved relationships between three chimpanzee sequences. Blue lines indicate conserved segments between the two haplotypes. Colors around the KIR gene names indicate the lineages: I (green), II (orange), III (purple) and V (red). (E–F) Orange: human and chimpanzee sequences are mixed with orthologs from other species. (0.05 MB PDF) [file pgen.1001192.s001.pdf]

# 5'UTR (1)

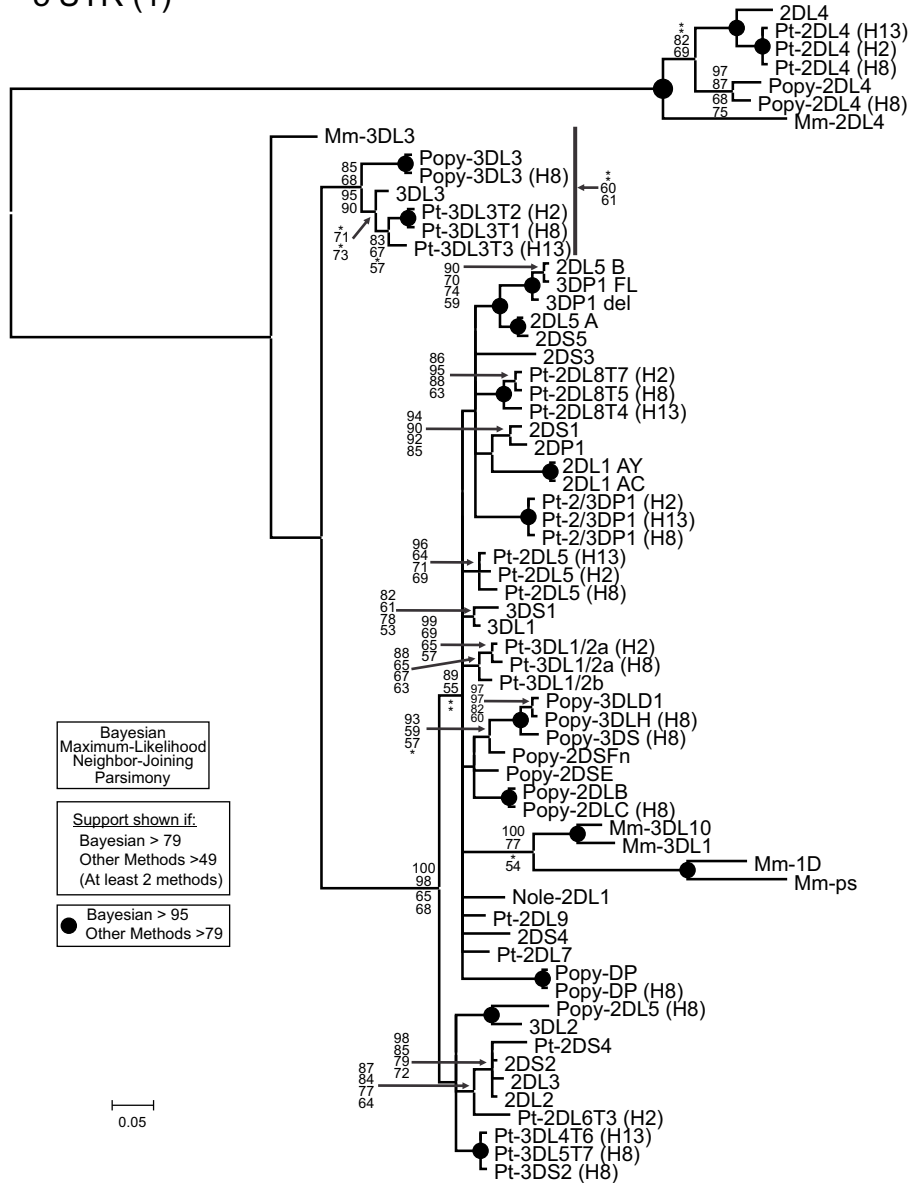

# E2-I2 (2)

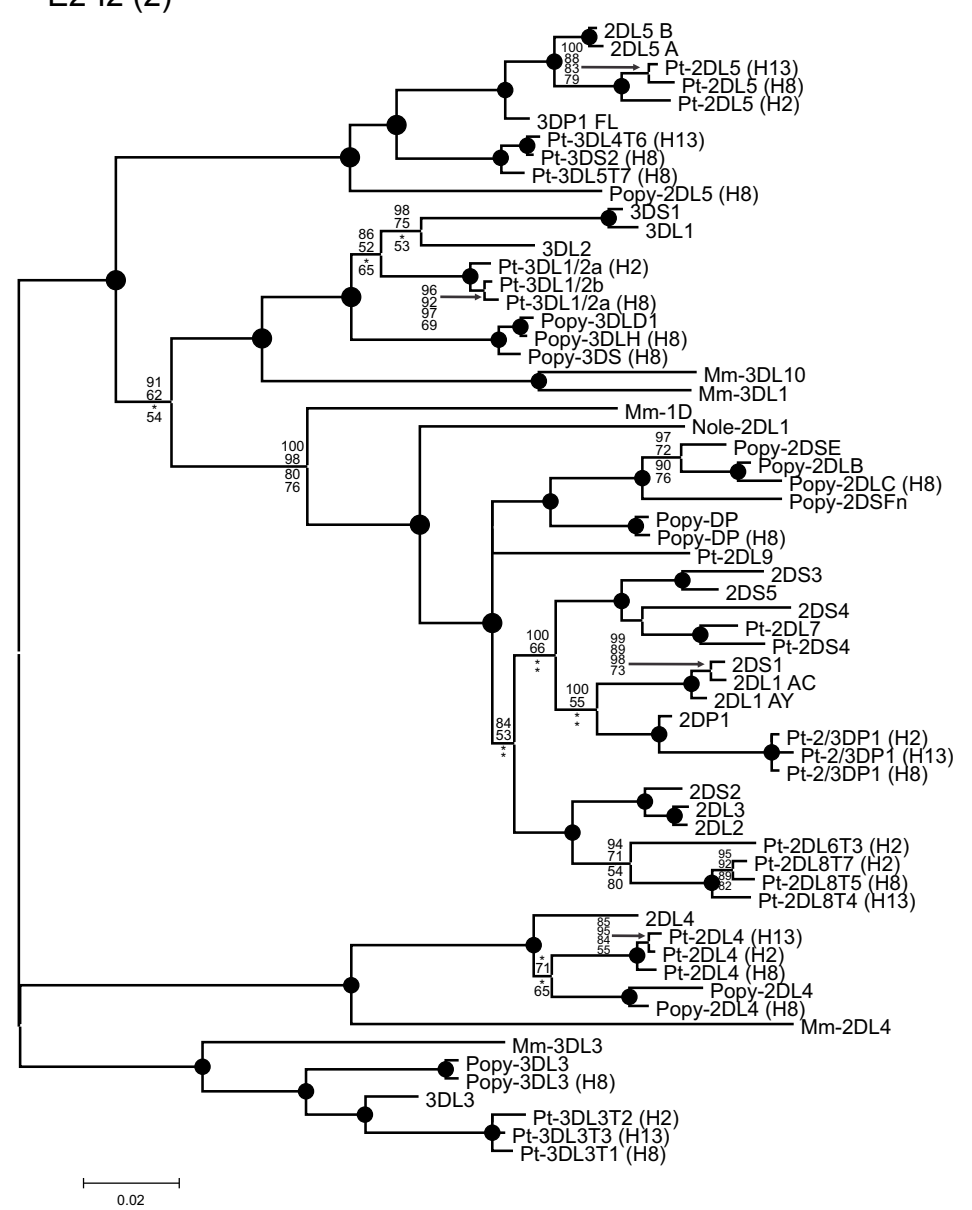

E3 (3)

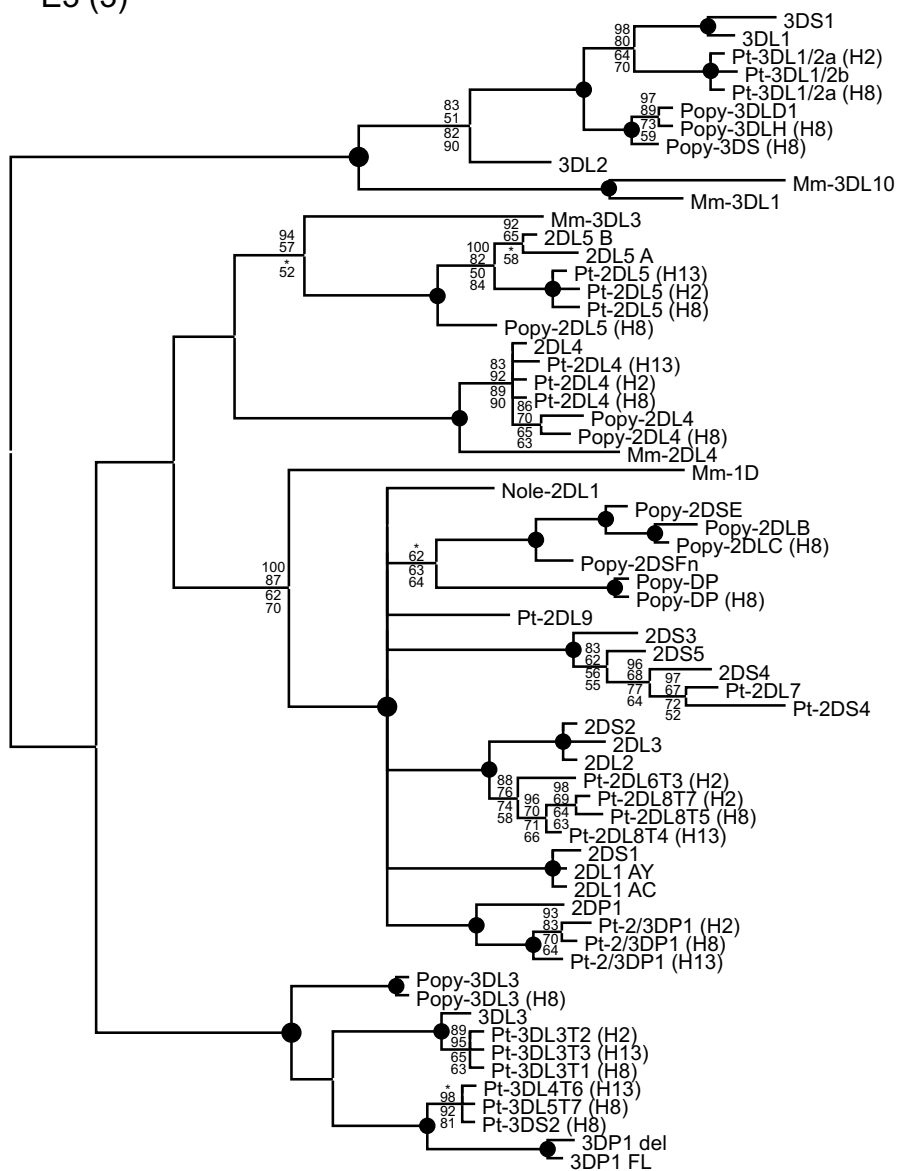

0.05

I3 (4)

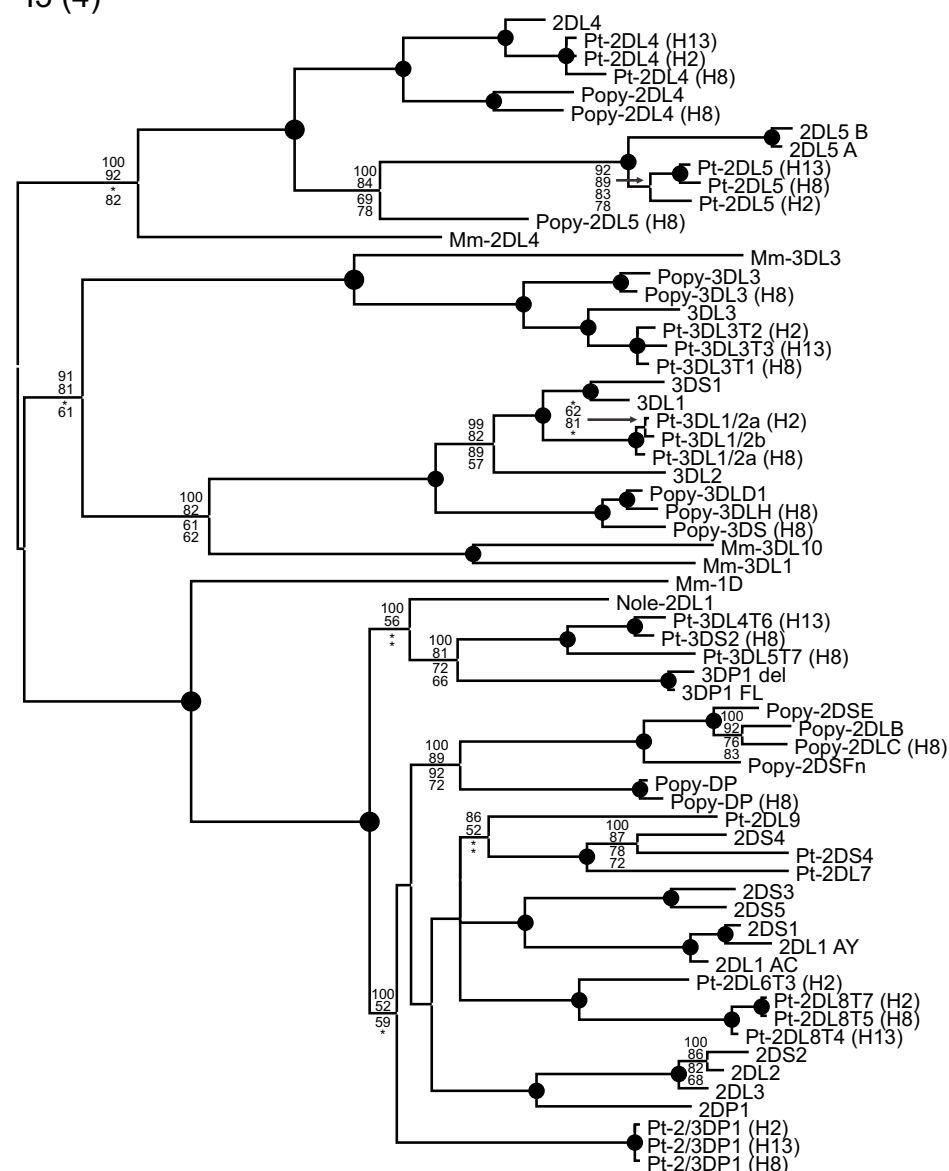

0.02

E4 (5)

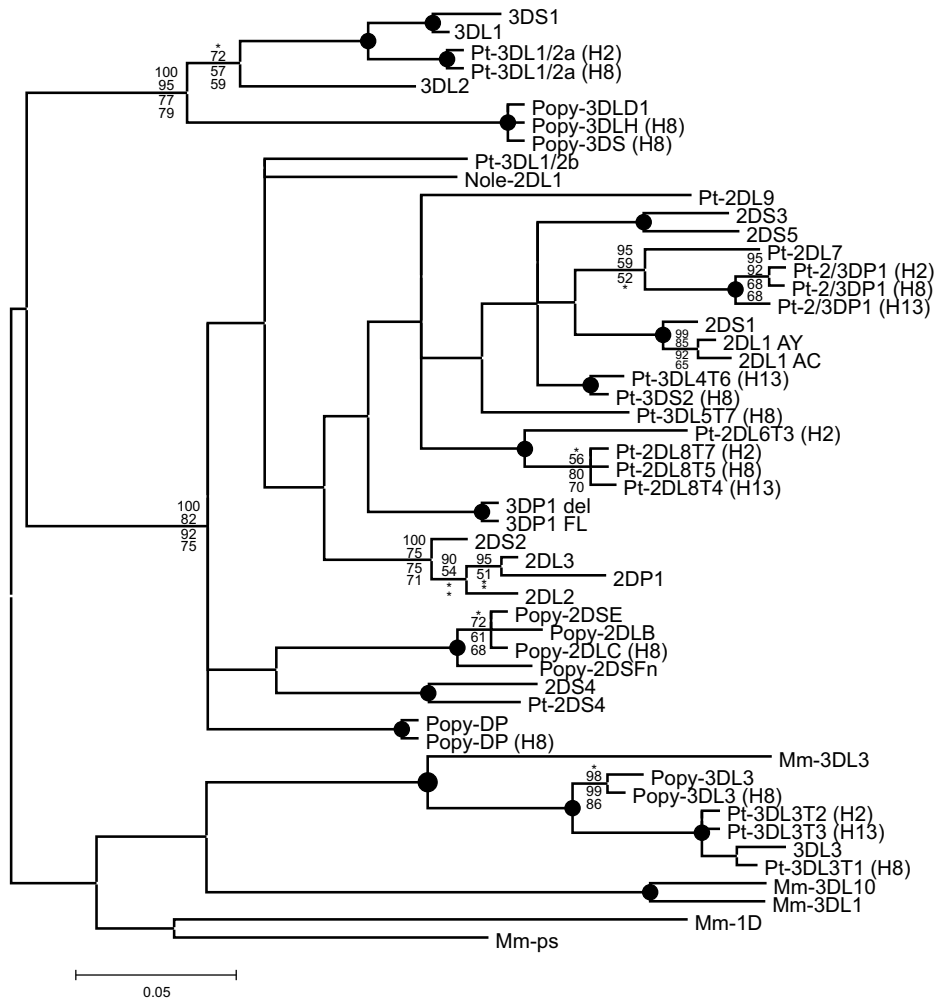

I4 (6)

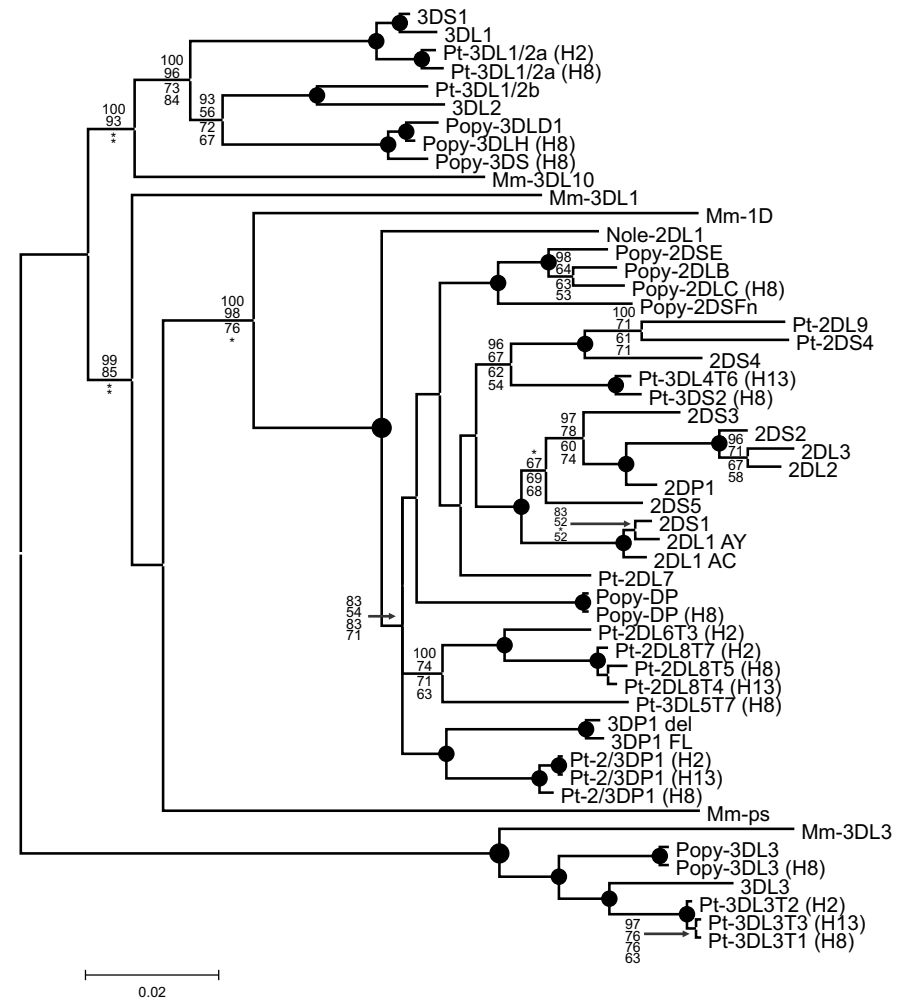

E5 (7)

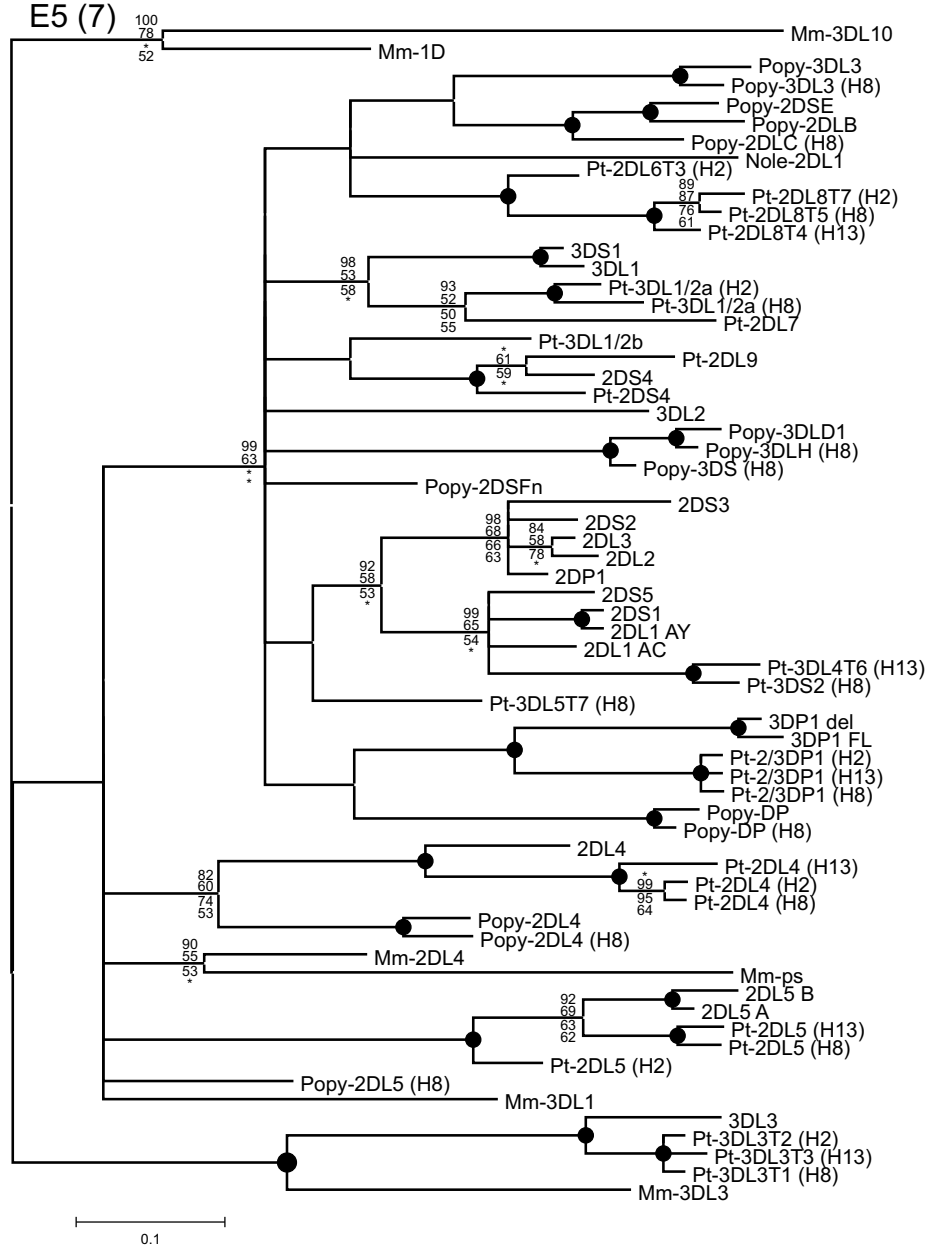

I5A (8)

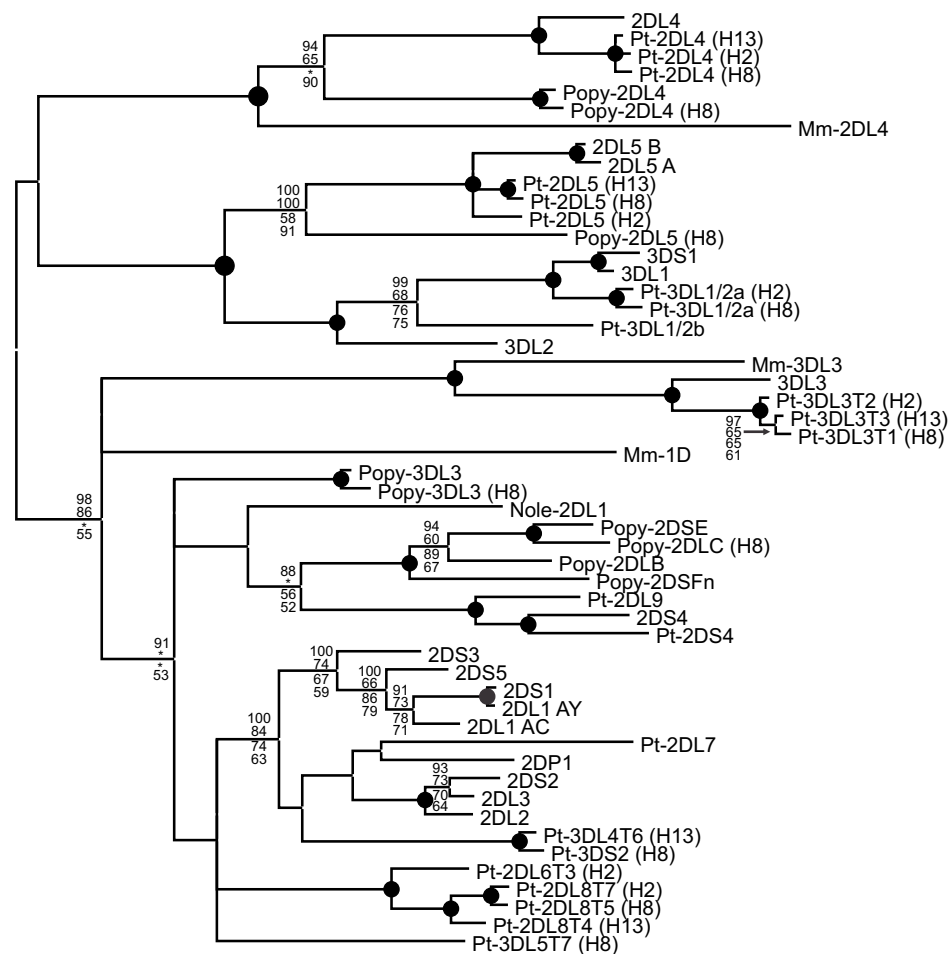

I5B (9)

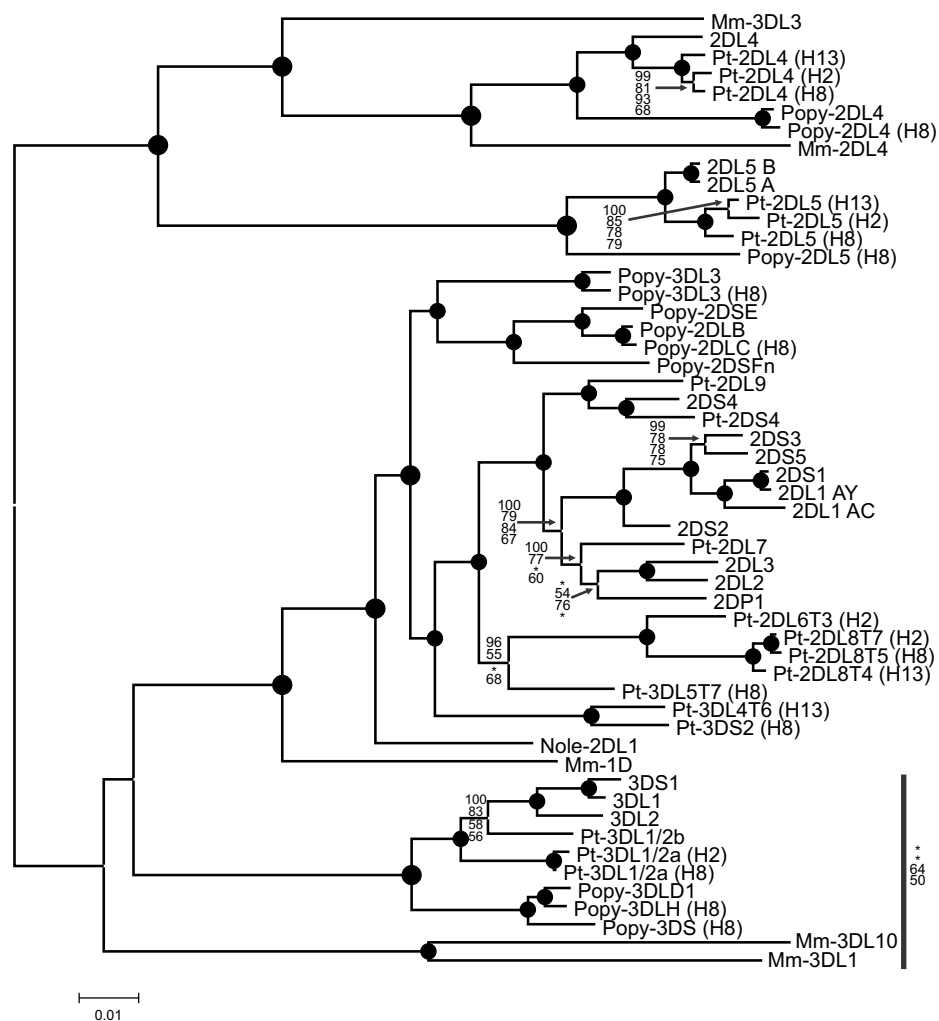

E6-I6A (10)

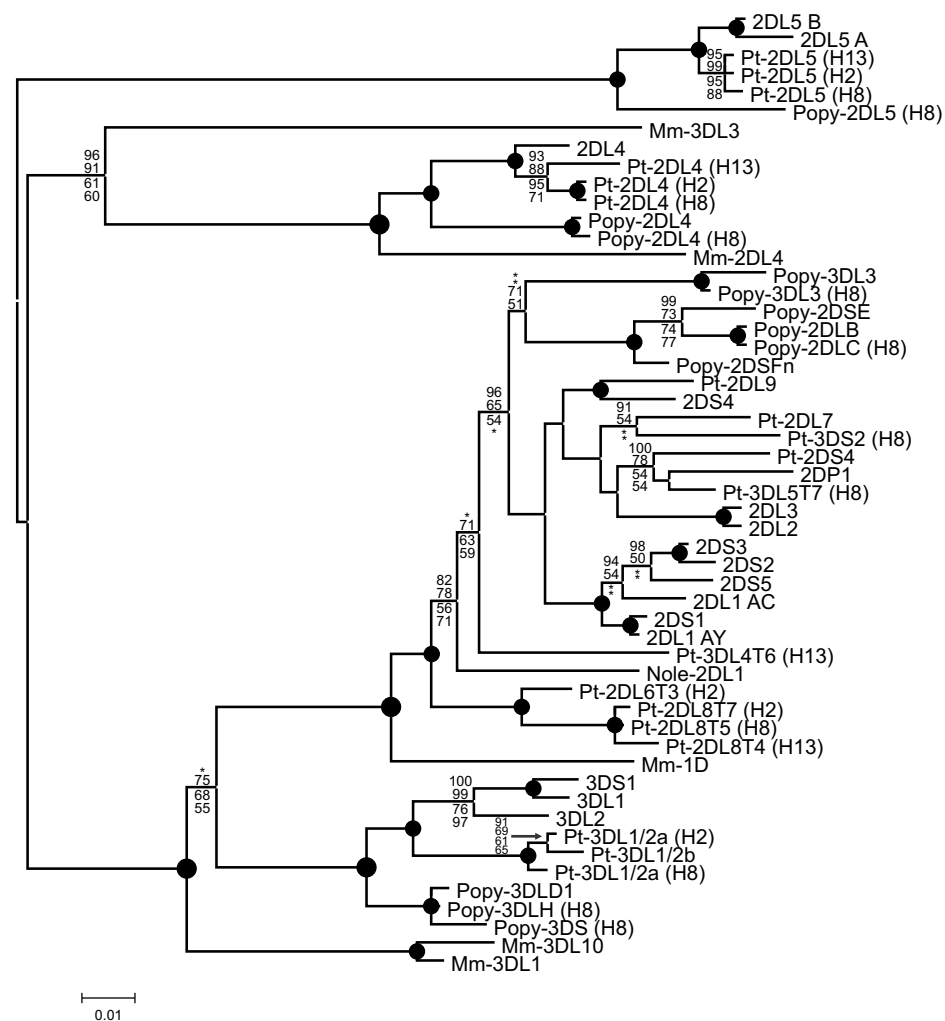

I6B (11)

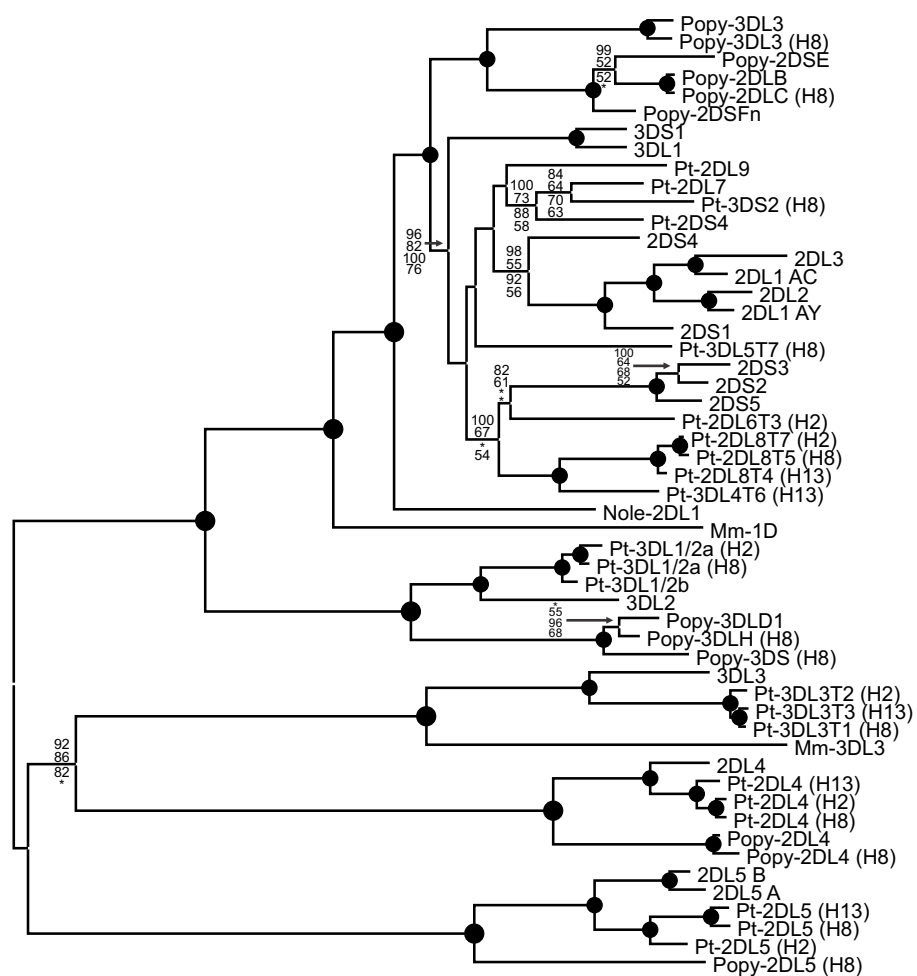

I6C (12)

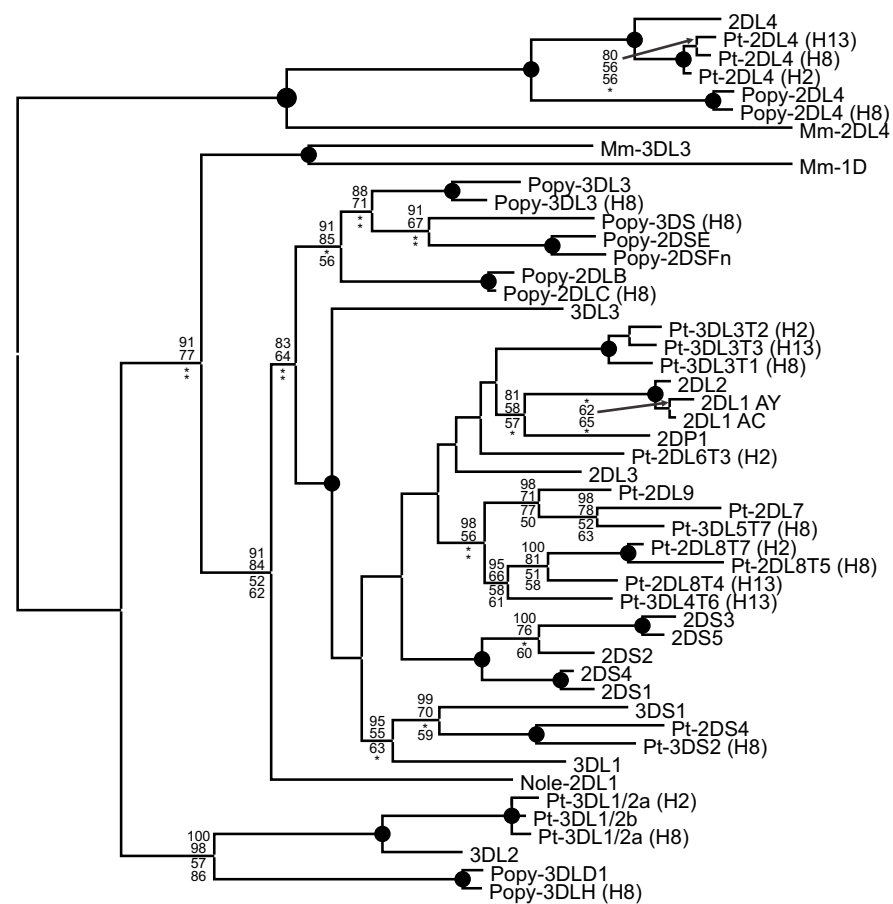

E7-I7-E8-I8-E9A (13)

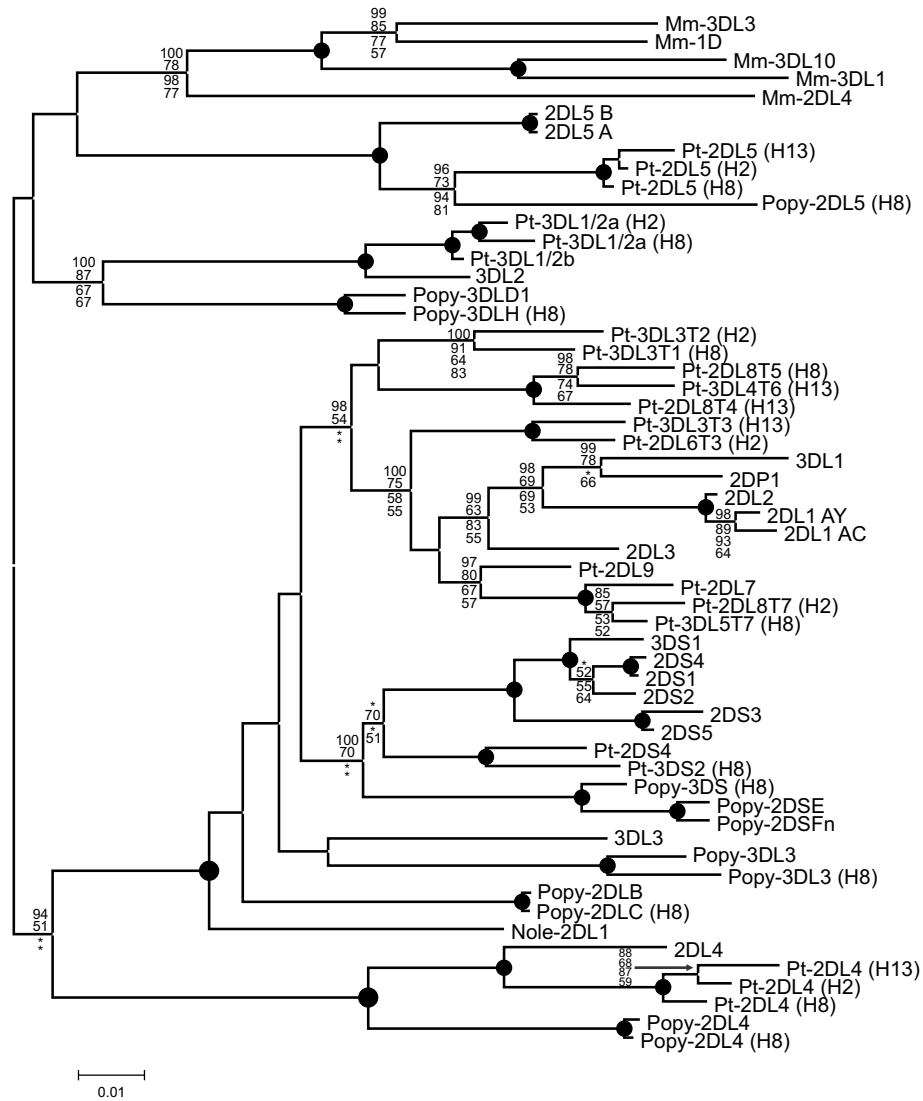

E9B (14)

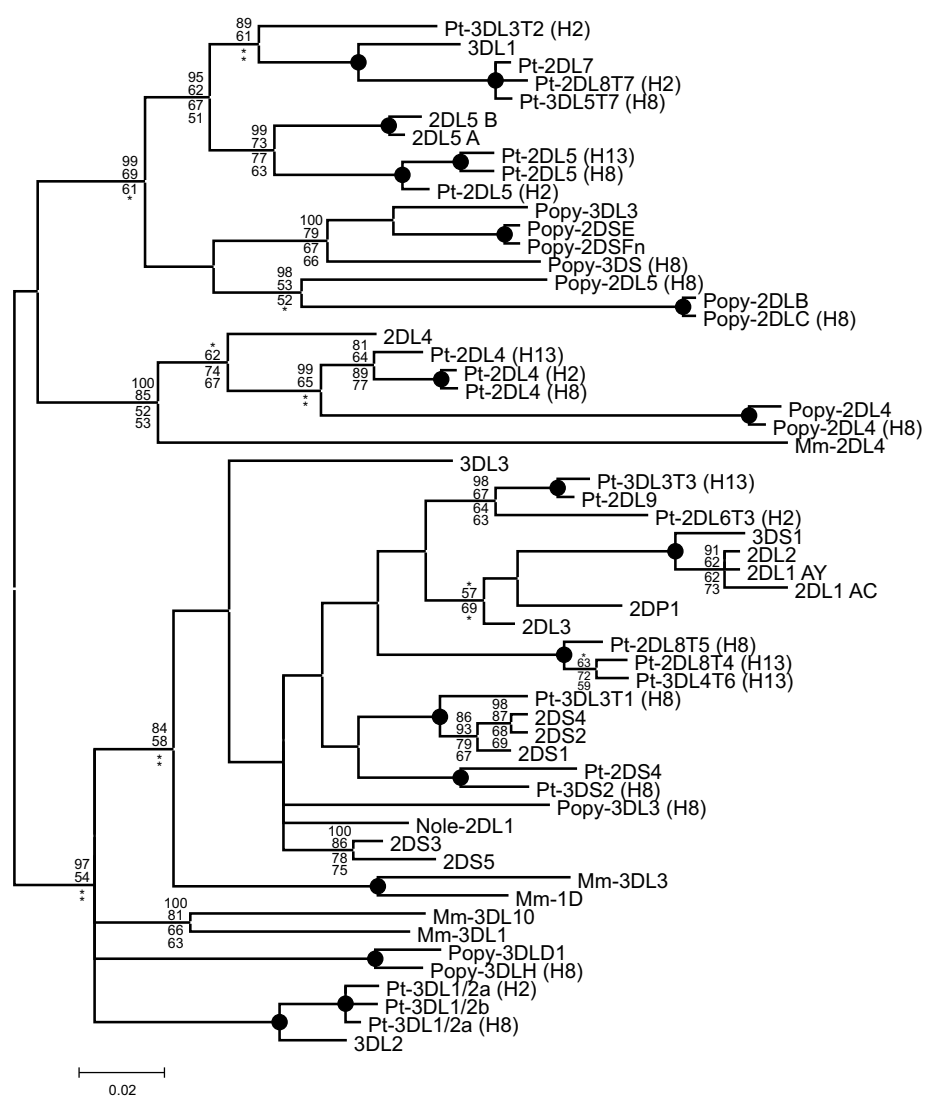

Supplement: Figure S2 — Phylogenetic analysis of the 14 genomic segments used to compare KIR haplotypes. The phylogenetic reconstruction was performed on each of the 14 segments described in Figure S1A using Bayesian, ML, NJ and parsimony approaches. The Bayesian tree topology was used for the display (with a midpoint rooting) and the support with the four methods indicated for all the nodes (from top to bottom: Bayesian, ML, NJ and parsimony). Black circles at nodes indicate a strong phylogenetic support: posterior probability (PP) >95 in the Bayesian analysis and bootstrap proportion (BP) ≥80 with the other three methods. The node support was omitted for the nodes not supported by at least two methods (PP ≥80 and BP ≥50). *: PP<80 or BP<50. (0.11 MB PDF) [file pgen.1001192.s002.pdf]

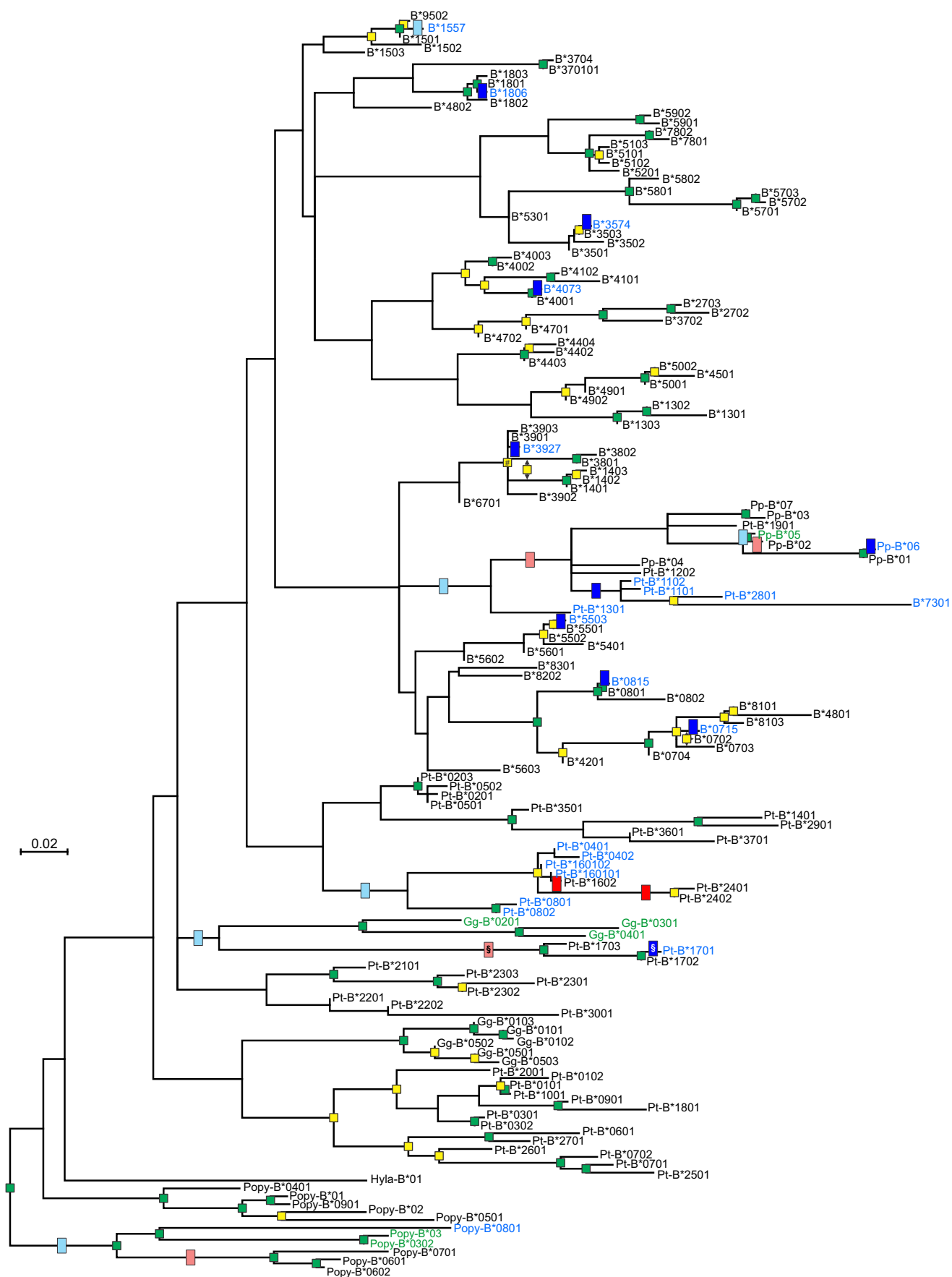

Supplement: Figure S3 — Emergence of MHC-B allotypes with V76. This phylogenetic tree represents the MHC-B subtree of the tree presented in Figure 6B with branch lengths estimated using the PAML codon model M2a. The names of allotypes with both V76 and N80 are blue while the names of allotypes with V76 but not N80 are green. Boxes along branches indicate amino acid changes at position 76 in the α 1 domain: from glutamic acid to valine (blue) or from valine to glutamic acid (red) (dark colors: p of change>0.95; light colors: p<0.95). At nodes, boxes indicate the phylogenetic support in the maximum likelihood analysis: yellow (BS ≥50) or green (BS ≥70). #, excluding B*3902. §, change from glycine to valine (blue box) or from glutamic acid to glycine (red box). (0.03 MB PDF) [file pgen.1001192.s003.pdf]

A

|      | C2 HLA-C | C1 HLA-C | C1 HLA-B |
|------|----------|----------|----------|
| 2DL1 | +        | -        | -        |
| 2DL2 | +        | +        | +        |
| 2DL3 | -        | +        | +        |

B

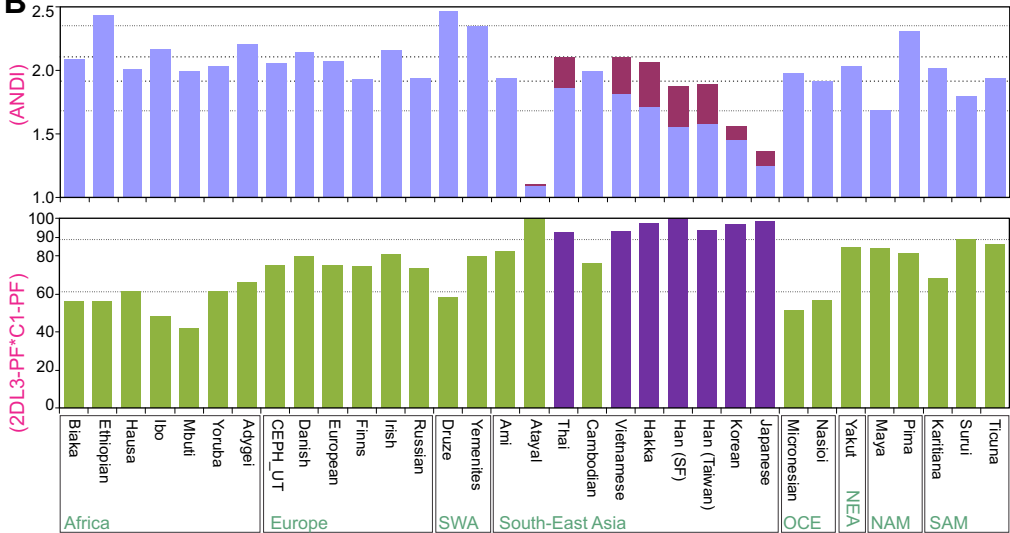

Supplement: Figure S5 — MHC-B allotypes that reacquire binding to lineage III KIR restore or increase NK cell effector capacity. (A) Summary of the KIR2DL/HLA-B (magenta) and KIR2DL/HLA-C (blue) interactions. (B) Average number of distinct KIR2DL-HLA interactions (ANDI) (top) and 2DL3PF*C1PF quantity (bottom; PF, phenotype frequency) in 33 human populations. Area between the gapped lines is the 25–75 percentile range; area between the dotted lines (top part only) is the non-outlier range (Whisker plot with 1.5 coefficient). Colors in the top part are as defined in (A). Populations in purple (bottom part) have HLA-B*46PF of 8.7–27.5%. SWA, Southwest Asia; OCE, Oceania; NEA, Northeast Asia; NAM, North America; SAM, South America. (0.03 MB PDF) [file pgen.1001192.s005.pdf]

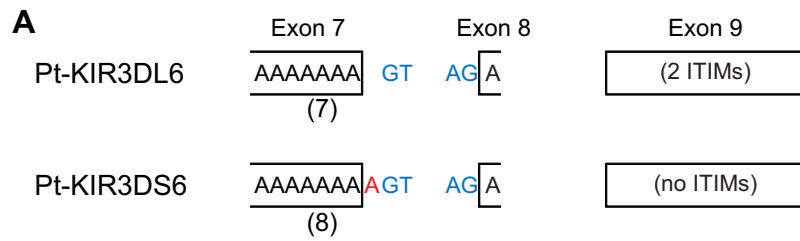

**B**

|           | 3DL6/S6 exon 7 |    |      |    |
|-----------|----------------|----|------|----|
|           | PCR 1          |    | PCR2 |    |
|           | 7A             | 8A | 7A   | 8A |
| Loulou    | 2              | 5  | 1    | 6  |
| Cheryl    | 2              | 2  | 0    | 4  |
| Kareem    | 0              | 1  | 1    | 1  |
| Nemo      | 0              | 4  | 1    | 3  |
| Buckwheat | 0              | 2  | 0    | 3  |
| Alex      | 2              | 9  | 2    | 10 |

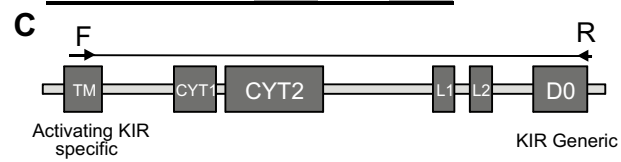

Supplement: Figure S10 — Sequence and position of Pt-KIR3DS6. (A) Sequence of Pt-KIR3DL6 and 3DS6 at the end of exon 7 and at the beginning of exon 8. Pt-KIR3DL6 has a stretch of seven adenosines at the end of exon 7 that maintains the typical KIR reading frame for exons 8 and 9, resulting in a protein with two ITIM in the cytoplasmic tail. Pt-KIR3DS6 has eight adenosines at the end of exon 7: this changes the typical KIR reading frame for exons 8 and 9, resulting in a protein with a short cytoplasmic tail with no ITIM. Nucleotides in blue represent splice sites. The adenosine in red represents the extra base pair of Pt-KIR3DS6 comparing to 3DL6. (B) Sequencing of exon 7 of Pt-KIR3DL6/S6 in six individuals that typed positives for Pt-KIR3DL6, including Alex, an individual used in the study where Pt-KIR3DL6 was characterized [23]. PCR1 and PCR2 represent two independent PCR amplifications. ‘7A’ and ‘8A’ refer to the number of adenosines at the end of exon 7, and represent Pt-KIR3DL6 and 3DS6, respectively. For each amplification and individual, several clones were sequenced, and the number of clones in each category is mentioned (for each amplification, the group with the largest number of clones is shaded in gray). (C) Schematic representation of the gene-to-gene PCR amplification used to characterize the position of Pt-KIR3DS6. F, forward primer; R, reverse primer. TM, exon encoding the transmembrane domain; CYT1-2, exons encoding the cytoplasmic tail. L1-2, exons encoding the leader peptide. (0.41 MB PDF) [file pgen.1001192.s010.pdf]

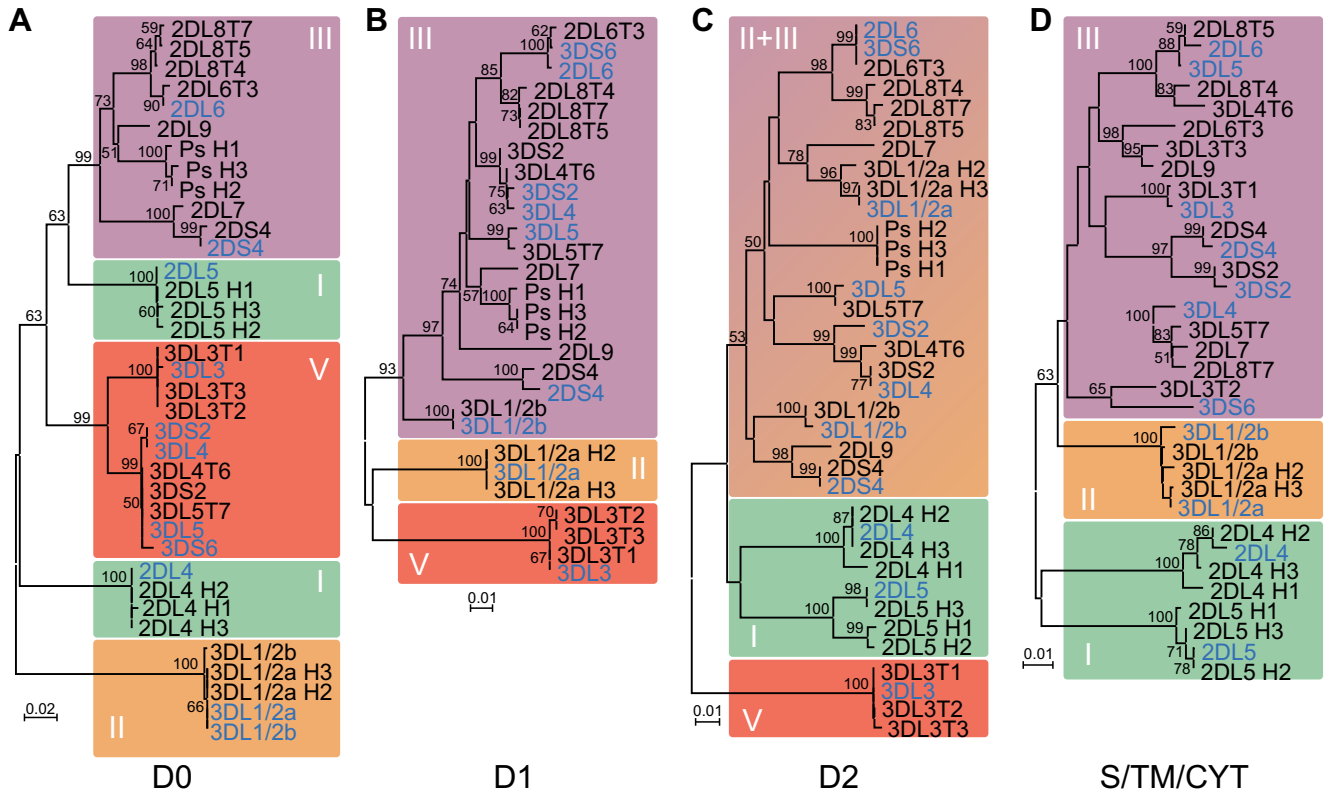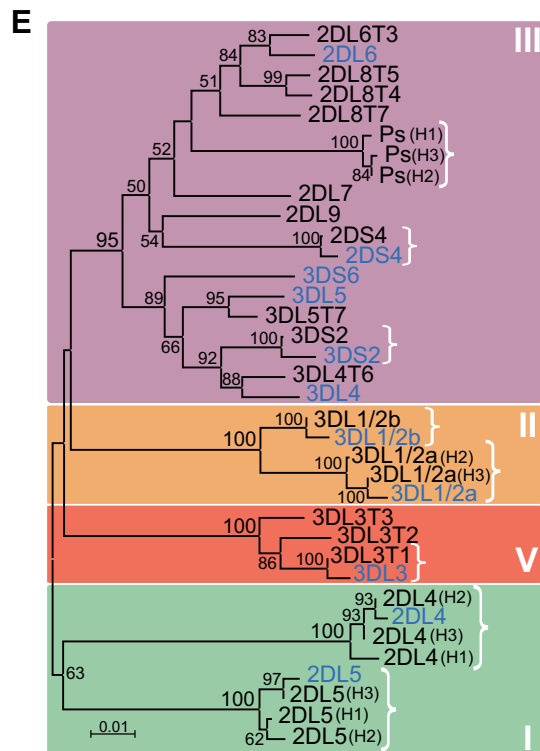

Supplement: Figure S13 — Domain-by-domain phylogenetic analysis of chimpanzee KIR sequences. The NJ method was used for the reconstructions and phylogenetic trees were rooted at the midpoint. For nodes, the bootstrap proportion support is given when >50. The eleven cDNA sequences described in the first study of chimpanzee KIR [23] are colored in blue. The five KIR lineages are delimited by boxes and are indicated with white roman letters. (A) D0 domain (or pseudoexon 3). (B) D1 domain. (C) D2 domain. (D) Stem, transmembrane and cytoplasmic domains (S/TM/CYT). (E) Full-length coding sequences (including the pseudoexon 3). White brackets indicate non-recombinant alleles. Allelic relationships were established based on the overall distance in the full-length sequence analysis and consistency in the domain-by-domain analysis (panels A-D). (0.03 MB PDF) [file pgen.1001192.s013.pdf]
