# Supplementary material for: Human-Specific Evolution and Adaptation Led to Major Qualitative Differences in the Variable Receptors of Human and Chimpanzee Natural Killer Cells
Source: PLoS Genet. 2010 Nov 4;6(11):e1001192. doi: 10.1371/journal.pgen.1001192 (PMC2973822; doi:10.1371/journal.pgen.1001192)
Supplement: Figure S4 — B*4601 is the only HLA-B allele with an allele frequency >25% in a large population. The two left-most columns list the 24 most common HLA-B alleles worldwide and their respective worldwide allele frequency (AF) in a study of 146 worldwide population samples [71]. Collectively these alleles represent >70% of worldwide HLA-B AF. Two alleles initially included (HLA-B*0704 and B*3705) were subsequently excluded as no data were available for them in the Allele Frequency Net Database [69]. For each allele we obtained the population with the highest allele frequency from the Allele Frequency Net Database [69]; only data from anthropologological studies involving at least 50 individuals were used: these populations and their AFs are listed in columns 3–4. In column 3, populations shaded in gray have a modern population size <150,000 individuals while populations shaded in dark gray have a modern population size <50,000 individuals. Columns 5–6 list for each of the 24 HLA-B alleles the name of the large population with the highest AF, and the AF in this population. While we set a minimum of ∼200,000 individuals for a population to be included in column 5, all the populations listed in this column have modern population sizes well in excess of 1,000,000 individuals. AF>25% are shaded in orange. (0.02 MB PDF) [file pgen.1001192.s004.pdf]

Allele	Worldwide AF	Population with highest Allele Frequency		Large population with highest Allele Frequency	
		Population	AF	Population	AF
B*3501	5.5%	USA South Dakota Lakota Sioux	25.2%	India Tamil Nadu Nadar	14.8%
B*5101	5.2%	Japan Ainu Hokkaido	19.0%	China Tibet Autonomous Region Tibetans	16.8%
B*4001	5.1%	Taiwan Taroko	35.5%	Taiwan Hakka	21.8%
B*4403	4.5%	Japan pop5	12.2%	Japan pop5	12.2%
B*4002	4.2%	Venezuela Perja Mountain Bari	39.8%	Japan Central	8.6%
B*0702	4.1%	Sweden Northern Sami	19.0%	Australia New South Wales	12.0%
B*1501	3.4%	Japan Ainu Hokkaido	29.0%	China Tibet Autonomous Region Tibetans	12.3%
B*0801	3.0%	Australia New South Wales	17.3%	Australia New South Wales	17.3%
B*5801	2.9%	China Guangdong Meizhou Han	17.0%	China Guangdong Meizhou Han	17.0%
B*1301	2.6%	Papua New Guinea Wosera	28.3%	China Guizhou Miao	21.1%
B*3701	2.5%	Cameroon Bakola Pygmy	10.6%	India Tamil Nadu Nadar	9.0%
B*4601	2.4%	China Dai Southwest	25.4%	China Dai Southwest	25.4%
B*5201	2.3%	Argentina Gran Chaco Eastern Toba	11.6%	Japan pop3	10.9%
B*1801	2.3%	Romanian	11.3%	Romanian	11.3%
B*4402	2.2%	Scotland Orkney	13.2%	Australia New South Wales	10.9%
B*3901	2.1%	Taiwan Saisiat	54.9%	Japan Central	4.4%
B*4006	1.8%	India West Bhils	19.0%	India West Bhils	19.0%
B*4801	1.8%	Taiwan Ami	26.0%	China North Han	5.7%
B*5301	1.6%	Burkina Faso Mossi	20.8%	Burkina Faso Mossi	20.8%
B*1302	1.4%	India West Coast Parsis	16.0%	China Beijing	7.6%
B*5601	1.4%	Papua New Guinea Madang	34.6%	China Yunnan Lisu	9.4%
B*5401	1.4%	USA Hawaii Okinawa	10.6%	Japan pop3	8.8%
B*1502	1.3%	China Yunnan Province Jinuo	23.8%	China Yunnan Province Wa	21.0%
B*2705	1.2%	Sweden Northern Sami	20.0%	Finland	6.1%
