# Supplementary material for: Human-Specific Evolution and Adaptation Led to Major Qualitative Differences in the Variable Receptors of Human and Chimpanzee Natural Killer Cells
Source: PLoS Genet. 2010 Nov 4;6(11):e1001192. doi: 10.1371/journal.pgen.1001192 (PMC2973822; doi:10.1371/journal.pgen.1001192)
Supplement: Figure S6 — Variability of KIR2DL2 and HLA-C2 frequencies in human populations. Amongst the components of the KIR2DL/HLA-C interactions HLA-C2 and KIR2DL2 display the widest range of phenotypic frequencies in populations (Figure 7C). HLA-C2 and KIR2DL2 frequencies also correlate with the average number of distinct KIR2DL/HLA-C interactions (A) and their combined frequency distribution (B) mimics that obtained with all KIR2DL-HLA-C interactions (Figure S5) indicating they represent the main source of HLA-C/KIR2DL variability in human populations. KIR2DL2 and HLA-C2 frequencies display a positive correlation (C–D) that amplifies the difference between populations. Indeed, five of the seven African populations are, for example, in the high range of the KIR2DL2-HLA-C2 frequencies (B) while six to eight of the ten Southeast Asian populations are in the low range of this distribution (B–D) and have KIR2DL3/HLA-C1 as their main KIR2DL-HLA-C interaction. The correlation between KIR2DL2 and HLA-C2 frequencies is the strongest amongst all KIR2DL-HLA-C1/C2 combinations (E), although the correlation between KIR2DS2 and HLA-C2 is almost equally as strong due to the strong linkage disequilibrium between KIR2DL2 and KIR2DS2. (A) Pearson product-moment correlations between the average number of distinct interactions (ANDI) and HLA-C1/C2, KIR2DL1-3 phenotypic frequencies. *, Average number of distinct interactions excluding the interactions involving HLA-B*46 and the Nasioi population (see panels C-E). (B) (2DL2PF*C2PF) quantity in 33 populations. Dark gray area represents the 25-75 percentile range. (C-D) Pearson product-moment correlations between KIR2DL2PF and HLA-C2PF in 33 (C) or 32 (D) populations. The red point in (C) is an outlier (Nasioi population) and was removed for the analysis in (D). (E) Pearson product-moment correlations between HLA-C1/C2 and KIR2DL1-3 phenotypic frequencies. The Nasioi population was consistently an outlier, indicating that the HLA-C1/C2 and KIR2DL1-3 frequenc [file pgen.1001192.s006.pdf]

A

Correlation		r	p	
ANDI*	C1	-0.578	<0.001	(0.0005)
	C2	0.823	<0.001	($7.1*10^{-9}$)
	2DL1	-0.376	<0.05	(0.0338)
	2DL2	0.861	<0.001	($2.6*10^{-10}$)
	2DL3	-0.532	<0.01	(0.0017)

B

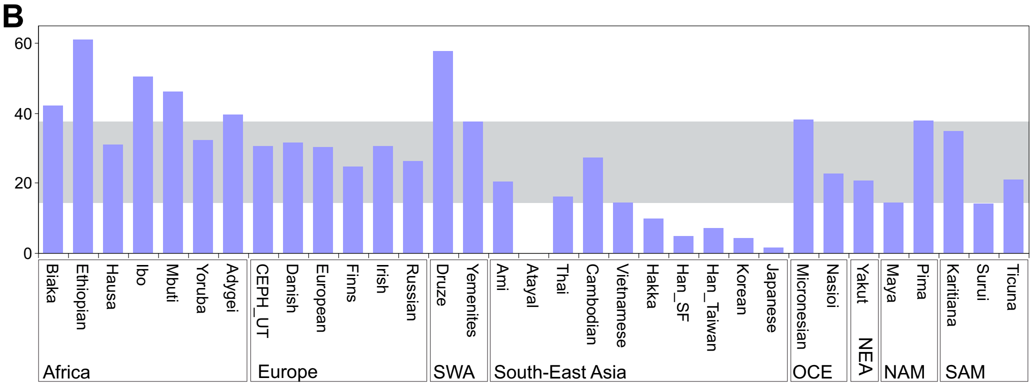

C

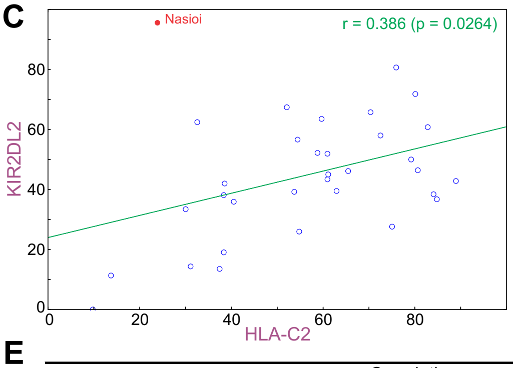

D

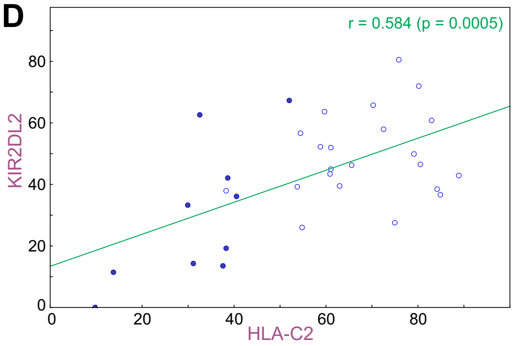

E

Combinations		Correlation					
		All populations (n=33)			All populations but Nasioi (n=32)		
KIR	HLA	r	p		r	p	
2DL1	C1	0.0072	NS	(0.9684)	0.0468	NS	(0.7992)
2DL1	C2	-0.0196	NS	(0.9139)	-0.0939	NS	(0.6092)
2DL2	C1	-0.4106	<0.05	(0.0176)	-0.5366	<0.01	(0.0015)
2DL2	C2	0.3862	<0.05	(0.0264)	0.5837	<0.001	(0.0005)
2DL3	C1	0.3061	NS	(0.0832)	0.4660	<0.01	(0.0072)
2DL3	C2	-0.2066	NS	(0.2486)	-0.4359	<0.05	(0.0126)
2DS1	C1	0.3997	<0.05	(0.0212)	0.3780	<0.05	(0.0329)
2DS1	C2	-0.3893	<0.05	(0.0251)	-0.3089	NS	(0.0854)
2DS2	C1	-0.3837	<0.05	(0.0275)	-0.4963	<0.01	(0.0039)
2DS2	C2	0.3948	<0.05	(0.0230)	0.5795	<0.001	(0.0005)
