# Supplementary material for: Human-Specific Evolution and Adaptation Led to Major Qualitative Differences in the Variable Receptors of Human and Chimpanzee Natural Killer Cells
Source: PLoS Genet. 2010 Nov 4;6(11):e1001192. doi: 10.1371/journal.pgen.1001192 (PMC2973822; doi:10.1371/journal.pgen.1001192)

A

			Comparisons		
			B vs C	B vs OWM	C vs OWM
Functional Divergence	Type I	θ_{ML}	0.430	0.442	0.433
		S.E.	0.045	0.041	0.055
		LRT	91.74	117.88	61.81
		p	9.9E-22	1.8E-27	3.8E-15
	Type II	θ_{II}	0.143	0.226	0.210
		S.E.	0.072	0.089	0.089
		z0	1.98	2.52	2.36
		p	0.048	0.012	0.018

C

	Comparisons		
Low Type I Divergence	B vs C	B vs OWM	C vs OWM
Cutoff (p)	<0.130	<0.142	<0.133

B

			Comparisons								
			MHC-B-specific			MHC-C-specific			Group-specific		
			B vs C	B vs OWM	C vs OWM	C vs B	C vs OWM	B vs OWM	B vs C	B vs OWM	C vs OWM
Functional divergence	Type I	Cutoffs G1	>0.730	>0.742	<0.543	>0.730	>0.733	<0.523	>0.730	>0.742	>0.733
		Cutoffs G2	>0.930	>0.942	<0.133	>0.930	>0.933	<0.142	>0.930	>0.942	>0.933
	Type II	Cutoffs G1	>0.443	>0.526	<0.388	>0.443	>0.510	<0.405	>0.443	>0.526	>0.510
		Cutoffs G2	>0.643	>0.726	=0.00	>0.643	>0.710	=0.00	>0.643	>0.726	>0.710

D

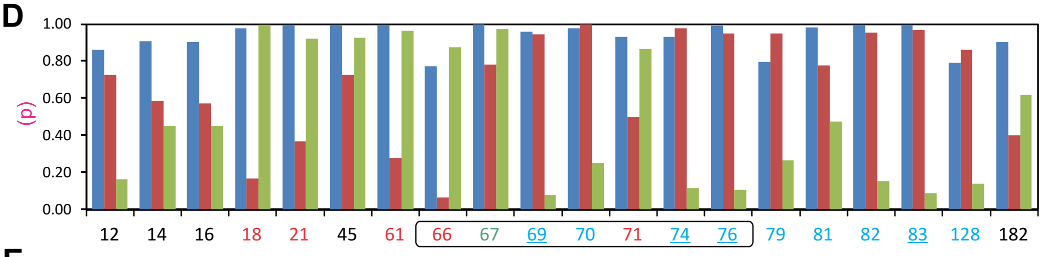

E

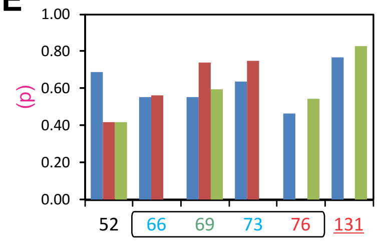

F

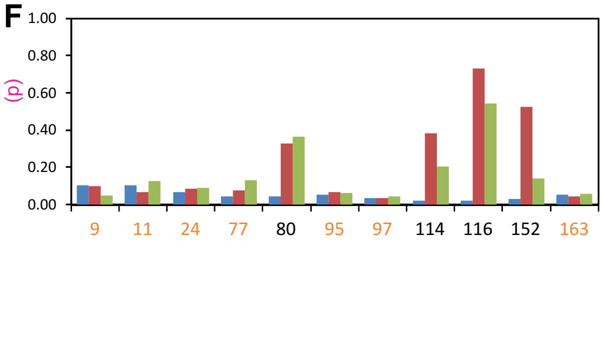

Supplement: Figure S7 — Functional divergence between the α 1 and α 2 domains of MHC-B and MHC-C. (A) Summary of the type I and type II functional divergence analyses. θ ML: coefficient of type I functional divergence. θ ΙΙ: coefficient of type II functional divergence. Significance of the type I analysis was assessed using a likelihood ratio test (LRT): twice the difference in likelihood (‘LRT’) was compared to a χ 2 distribution with one degree of freedom. For the type II analysis a two-tailed Z-test was used to assess the significance. S.E., Standard Error. (B) Identification of the type I and type II functional divergence-related residues (defined as having a posterior probability to be functional divergence-related (p) >θ ML/ΙΙ+0.3 [Gu]). Group-specific residues are functional divergence-related in all three comparisons while MHC-B or -C specific positions are related to functional divergence in two of the three comparisons and display an average, or lower than average, functional divergence in the third comparison. For the type I divergence, MHC-B specific positions were defined as related to functional divergence in the MHC-B/MHC-C and MHC-B/OWM comparisons (G1: p>θ ML+0.3; G2: p>θ ML+0.5) but not in the MHC-C/OWM comparison (G1: p<θ ML+2S.E.; G2: p<θ ML-0.3). For the type II divergence, MHC-B specific positions were defined as related to functional divergence in the MHC-B/MHC-C and MHC-B/OWM comparisons (G1: p>θ ΙΙ+0.3; G2: p>θ ΙΙ+0.5) but not in the MHC-C/OWM comparison (G1: p<θ ΙΙ+2S.E.; G2: p = 0). The same approach was used for the MHC-C specific positions. OWM, Old World monkey. (C) Identification of residues with a low type I functional divergence (defined as having p<θ ML-0.3). (D-F) Results of the type I (D,F) and type II (E) functional divergence analyses. Functionally-divergent sites are listed at the bottom of each graph. MHC-B specific sites are colored blue, MHC-C specific sites are colored red. Sites that are functionally-divergent in all three comparisons are green, [file pgen.1001192.s007.pdf]
