# Supplementary material for: Human-Specific Evolution and Adaptation Led to Major Qualitative Differences in the Variable Receptors of Human and Chimpanzee Natural Killer Cells
Source: PLoS Genet. 2010 Nov 4;6(11):e1001192. doi: 10.1371/journal.pgen.1001192 (PMC2973822; doi:10.1371/journal.pgen.1001192)
Supplement: Figure S8 — Disease associations with HLA-B*46. The ‘Disease’ column on the left lists the name of the disease investigated, and for infectious diseases the name of the causal pathogen. The country where the study was conducted, the type of association (resistance ‘R’ or susceptibility ‘S’) and the reference to the study are given in the ‘Association’ column. The column ‘Significance’ gives the significance of the association between the disease and HLA-B*46 (‘c’ indicates that this probability was corrected for multiple comparisons while ‘nc’ indicates no correction for multiple comparisons). §, the significance is for a subgroup of HLA-B*46 haplotypes (listed in ‘Other HLA factors’). The column ‘Other HLA factors’ lists genetic factors also found to be associated with the disease in the same study, and genetic factors in bold displayed a more significant association than B*46. Brackets in the ‘Other HLA factors’ column designate haplotypes. #, only in males. References are: [Wang LM, Kimura A, Satoh M, Mineshita S (1999) HLA linked with leprosy in southern China: HLA-linked resistance alleles to leprosy. Int J Lepr Other Mycobact Dis 67: 403-408. Hananantachai H, Patarapotikul J, Ohashi J, Naka I, Looareesuwan S, et al. (2005) Polymorphisms of the HLA-B and HLA-DRB1 genes in Thai malaria patients. Jpn J Infect Dis 58: 25-28. Chandanayingyong D, Maranetra N, Bovornkitti S (1988) HLA antigen profiles in Thai tuberculosis patients. Asian Pac J Allergy Immunol 6: 77-80. Blackwell JM, Jamieson SE, Burgner D (2009) HLA and infectious diseases. Clin Microbiol Rev 22: 370-385. Huang X, Ling H, Mao W, Ding X, Zhou Q, et al. (2009) Association of HLA-A, B, DRB1 alleles and haplotypes with HIV-1 infection in Chongqing, China. BMC Infect Dis 9: 201. Lin M, Tseng HK, Trejaut JA, Lee HL, Loo JH, et al. (2003) Association of HLA class I with severe acute respiratory syndrome coronavirus infection. BMC Med Genet 4: 9. Yoon SK, Han JY, Pyo CW, Yang JM, Jang JW, et al. (2005) Association betwe [file pgen.1001192.s008.pdf]

Disease		Association				
Name	Pathogen	Country	Type	Significance	Other HLA factors	Reference
Leprosy (Lepromatous leprosy)	*M. leprae*	China	P	<0.01 nc[§]	[B46-MICA-5A5]	Wang et al, 1999
Malaria (Severe vs Cerebral Malaria)	*P. falciparum*	Thailand	S	=0.005 nc	---	Hananantachai al, 2005
Tuberculosis (Pulmonary tuberculosis)	*M. tuberculosis*	Thailand	S	---	DR4	Chandanayingyong et al, 1988
HIV/AIDS (Infection)	HIV type 1	China	S	<0.05 c	---	Huang et al, 2009
SARS (Severe cases vs high risk workers)	SARS coronavirus	Taiwan	S	=0.0008 nc; =0.0279 c	---	Lin et al, 2003
Hepatitis C (Chronic infection)	Hepatitis C virus	Korea	S	<0.02 nc	A3, B35, **DRB1*0803**, DQB1(*0601,*0604)	Yoon et al, 2005
Haemorrhagic fever with renal syndrome	Hantaan virus	China	S	=0.034 nc	**DRB1*09**	Wang et al, 2009
Nasopharyngeal carcinoma	---	Taiwan	S	=0.04 nc	**A*0207**	Hildesheim et al, 2002
		China	S	<0.01 nc[§]	[A*0207+ B*4601+]	Tang et al, 2010
			P	=0.0325 nc[§]	[A*0207- B*4601+]	
Familial vitiligo	---	Japan	S	<1x10E-8 c	---	Ando et al, 1993
Myasthenia gravis	---	Taiwan	S	<0.01 c	**DR9**	Chen et al, 1993
Graves' disease	---	Japan	S	<0.003 c	---	Naito et al, 1987
		Japan	S	<0.015 c	**Cw1**	Onuma et al, 1994
		China	S	<6x10E-4 c[#]	DR9[#], **DQB1*0303**[#]	Cavan et al, 1994
