# Supplementary material for: Human-Specific Evolution and Adaptation Led to Major Qualitative Differences in the Variable Receptors of Human and Chimpanzee Natural Killer Cells
Source: PLoS Genet. 2010 Nov 4;6(11):e1001192. doi: 10.1371/journal.pgen.1001192 (PMC2973822; doi:10.1371/journal.pgen.1001192)
Supplement: Figure S9 — Relationships between chimpanzee KIR cDNA and gene sequences. Results of the expression study in peripheral blood mononuclear cells are given for the ten KIR gene sequences with no prior cDNA equivalent. *, Pt-KIR3DL3 is from the same lineage as KIR3DL3, a gene expressed at low or undetectable levels in peripheral blood NK cells. (0.01 MB PDF) [file pgen.1001192.s009.pdf]

Gene	cDNA
2DL4 (H2,H8,H13)	2DL4
2DL5 (H2,H8,H13)	2DL5
3DL1/2a (H2,H8)	3DL1/2a
3DL1/2b (H13)	3DL1/2b
2DS4 (H13)	2DS4
3DS2 (H8)	3DS2
3DL3T1 (H8)	3DL3
-----	2DL6
-----	3DL4
-----	3DL5
-----	3DS6
3DL4T6 (H13)	Expressed
2DL8T4 (H13)	Expressed
2DL7 (H13)	Expressed
3DL3T3 (H13)	Expressed
2DL6T3 (H2)	Expressed
2DL8T7 (H2)	Expressed
3DL3T2 (H2)	Untested*
2DL8T5 (H8)	Expressed
2DL9 (H8)	Expressed
3DL5T7 (H8)	Expressed
