# Supplementary material for: Human-Specific Evolution and Adaptation Led to Major Qualitative Differences in the Variable Receptors of Human and Chimpanzee Natural Killer Cells
Source: PLoS Genet. 2010 Nov 4;6(11):e1001192. doi: 10.1371/journal.pgen.1001192 (PMC2973822; doi:10.1371/journal.pgen.1001192)
Supplement: Figure S11 — KIR and HLA frequencies used to establish the average number of distinct KIR2DL-HLA-B/C interactions (ANDI) in human populations. AFR, Africa; EUR, Europe; SWA, Southwest Asia; EAS, East Asia; OCE, Oceania; NEA, Northeast Asia; NAM, North America; SAM, South America. Numbers in parenthesis indicate the number of populations in each group. References are: [69] [Single RM, Martin MP, Gao X, Meyer D, Yeager M, et al. (2007) Global diversity and evidence for coevolution of KIR and HLA. Nat Genet 39: 1114-1119. Norman PJ, Stephens HA, Verity DH, Chandanayingyong D, Vaughan RW (2001) Distribution of natural killer cell immunoglobulin-like receptor sequences in three ethnic groups. Immunogenetics 52: 195-205. Toneva M, Lepage V, Lafay G, Dulphy N, Busson M, et al. (2001) Genomic diversity of natural killer cell receptor genes in three populations. Tissue Antigens 57: 358-362. Hoa BK, Hang NT, Kashiwase K, Ohashi J, Lien LT, et al. (2008) HLA-A, -B, -C, -DRB1 and -DQB1 alleles and haplotypes in the Kinh population in Vietnam. Tissue Antigens 71: 127-134. Whang DH, Park H, Yoon JA, Park MH (2005) Haplotype analysis of killer cell immunoglobulin-like receptor genes in 77 Korean families. Hum Immunol 66: 146-154. Lee KW, Oh DH, Lee C, Yang SY (2005) Allelic and haplotypic diversity of HLA-A, -B, -C, -DRB1, and -DQB1 genes in the Korean population. Tissue Antigens 65: 437-447. Yawata M, Yawata N, Draghi M, Little AM, Partheniou F, et al. (2006) Roles for HLA and KIR polymorphisms in natural killer cell repertoire selection and modulation of effector function. J Exp Med 203: 633-645. *, some phenotypic frequencies were estimated from allele frequencies assuming Hardy-Weinberg equilibrium. (0.02 MB PDF) [file pgen.1001192.s011.pdf]

Populations		KIR2DL1-3	HLA-C1/C2	HLA-B46
AFR(7), EUR(6), SWA(2) OCE(2), NEA(1), NAM(2), SAM(3)		Reference 1		
SEA	Ami			Reference 2
	Atayal			
	Thailand	Reference 3	Reference 2*	
	Cambodian	Reference 1		
	Vietnam	Reference 4	Reference 5*	
	Hakka	Reference 1		Reference 2
	Han_SF			
	Han_Taiwan			
	Korea	Reference 6	Reference 7*	
	Japan	Reference 8		
