# Supplementary material for: Human-Specific Evolution and Adaptation Led to Major Qualitative Differences in the Variable Receptors of Human and Chimpanzee Natural Killer Cells
Source: PLoS Genet. 2010 Nov 4;6(11):e1001192. doi: 10.1371/journal.pgen.1001192 (PMC2973822; doi:10.1371/journal.pgen.1001192)
Supplement: Figure S12 — Alternative model for the interactions between HLA-C and KIR2DL. Unlike the analyses presented in Figure 7, Figure S5, and Figure S6, the interaction between KIR2DL2 and HLA-C2 was not taken into account in this model. (A) Summary of the KIR2DL/HLA-B (magenta) and KIR2DL/HLA-C (blue) interactions. (B) Average number of distinct KIR2DL-HLA interactions (ANDI) in 33 human populations. Area between the gapped lines is the 25-75 percentile range; area between the dotted lines is the non-outlier range (Whisker plot with 1.5 coefficient). Colors in the top part are as defined in (A). SWA, Southwest Asia; OCE, Oceania; NEA, Northeast Asia; NAM, North America; SAM, South America. (C) Pearson product-moment correlations between the average number of distinct interactions (ANDI) and HLA-C1/C2, KIR2DL1-3 phenotypic frequencies. *, excluding the interactions involving HLA-B*46 and the Nasioi population (see Figure S6C-E). (D) KIR-HLA phenotypic frequencies for five individual populations. Maximum: maximum ANDI assuming Hardy-Weinberg equilibrium. (0.03 MB PDF) [file pgen.1001192.s012.pdf]

A

	C2 HLA-C	C1 HLA-C	C1 HLA-B
2DL1	+	-	-
2DL2	-	+	+
2DL3	-	+	+

B

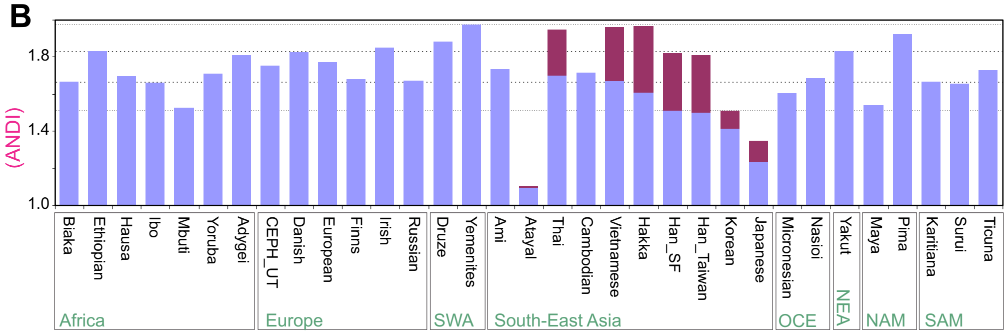

C

Correlation		r		p
ANDI*	C1	-0.298	NS	(0.0924)
	C2	0.649	<0.001	($4.3*10^{-5}$)
	2DL1	-0.291	NS	(0.1003)
	2DL2	0.630	<0.001	($8.6*10^{-5}$)
	2DL3	-0.234	NS	(0.1900)

D

	Phenotypic Frequency (%)						ANDI
	**C1**	**C2**	**B46**	**2DL1**	**2DL2**	**2DL3**	
Atayal	100	9.8	0.9	100	0	100	1.11
Japanese	98.3	13.8	10.3	100	11.4	100	1.35
Korean	97.3	31.1	8.5	99.4	14.3	99.4	1.51
Druze	73.9	80.2	--	96.5	71.9	78.4	1.88
Pima	92.6	59.6	--	87.9	63.6	87.6	1.92
Yemenites	83.9	80.6	--	97.7	46.5	95.2	1.98
Maximum	84.0	64.0	--	100	75.0	75.0	1.90
