# Supplementary material for: Human-Specific Evolution and Adaptation Led to Major Qualitative Differences in the Variable Receptors of Human and Chimpanzee Natural Killer Cells
Source: PLoS Genet. 2010 Nov 4;6(11):e1001192. doi: 10.1371/journal.pgen.1001192 (PMC2973822; doi:10.1371/journal.pgen.1001192)
Supplement: Figure S14 — Summary of KIR polymorphism in chimpanzee. Amino acid variation is listed for 12 of the 13 chimpanzee KIR for which such data are available (A-L). For each KIR, the positions of variability are displayed. The origin of each sequence is given in parenthesis: Hxx and Tx (KIR haplotypes), cDNA (first cDNA study [23]), cDNA2 (cDNA sequences characterized in the present study), and cDNA3 (unpublished sequences deposited in Genbank with the following accession numbers: AM279149, AM292657-63, AM396937, and AM400232-36). Position, amino acid residue in the mature protein. L, leader peptide. D0-D2, Ig-like domains. S, stem. TM, transmembrane domain. CYT, cytoplasmic tail. FS, frameshift. *, Stop codon. (0.03 MB PDF) [file pgen.1001192.s014.pdf]

A

Position	126	227	231	284	316
Domain	D2	TM		CYT	
2DL4*001 (H13)	W	A	L	I	R
2DL4*002 (H8/cDNA)	R	.	F	V	.
2DL4*003 (H2)	R	V	.	V	K

B

Position	-13	15	75	104	114	151	171	176	188	191	227
Domain	L	D0		D2							TM
2DL5*001 (H13)	A	F	R	L	R	I	T	F	H	L	V
2DL5*002 (v2 CDNA)	-	.	.	S	.	.	.	.	.	.	A
2DL5*003 (H8)	V	Y	.	S	.	.	.	.	.	.	A
2DL5*004 (H2)	.	.	Q	S	H	V	R	L	D	P	A
2DL5*005 (v1 cDNA)	-	.	.	S	H	V	R	L	D	P	A

C

Position	5	6	61	229	253
Domain	D1			TM	CYT
2DS4*001 (cDNA)	H	T	T	M	N
2DS4*002 (H13)	Q	I	I	T	(FS)

D

Position	5	131	137	138	139	141	142	145	147	148	153	163	164	165	177	179	180	193	194	212	216	225	233	241	242	243	246	250	253	255	267	277	285	286	315	320	345	415
Domain	D0	D1																		D2															S	TM	CYT	
3DL1/2a*00101 (H8)	H	F	I	S	Q	P	S	V	Q	I	S	M	L	A	T	T	P	M	V	Q	N	R	F	C			R	K	R	F	R	R	D	P	R	I	E	P
3DL1/2a*00102 (v2 cDNA)	.	.	.	.	.	.	.	.	.	.	.	.	.	.	.	.	.	.	.	.	.	.	.	.			.	.	.	.	.	.	.	.	.	.	.	.
3DL1/2a*002 (H2/v1 cDNA)	.	.	.	.	.	.	.	.	.	.	.	.	.	.	.	.	.	.	.	.	S	.	.	.			P	.	.	.	.	.	.	.	.	V	G	.
3DL1/2a*003 (v3 cDNA)	Q	.	.	.	.	.	.	.	.	.	.	.	.	.	.	.	.	.	.	.	.	.	.	.			.	.	.	.	.	.	.	.	L	.	.	.
3DL1/2b*001 (cDNA)	.	L	K	V	E	T	L	I	E	L	F	T	H	V	P	F	R	V	I	L	.	S	S	R	E	L	P	S	G	L	G	P	N	S	.	V	.	L
3DL1/2b*002 (H13)	.	L	K	V	E	T	L	I	E	L	F	T	H	V	P	F	R	V	I	L	.	S	S	R	E	L	P	S	G	L	G	P	N	S	.	V	.	.

E

Position	54	68	83	220	225	231	232	236	238	241	250	253	260	266	268	284	286	296	297	300	309
Domain	D1			S	TM						CYT										
2DL6*001 (T3/v2 cDNA3)	E	H	L	P	L	V	A	F	P	L	N	N	E	T	S	Q	N	T	R	Q	I
2DL6*002 (v1 cDNA3)	G	.	.	.	.	.	.	.	.	.	.	(FS)									
2DL6*003 (cDNA)	.	R	V	S	V	A	V	L	L	F	K	.	Q	I	K	H	D	N	P	R	T

F

Position	99	170
Domain	D1	D2
2DL9*001 (H8)	I	T
2DL9*002 (v1 cDNA3)	.	A
2DL9*003 (v2 cDNA3)	T	.

G

Position	144	145	190	218	219	220	236	241	266	268	284	286	296	300	310
Domain	D2			S			TM		CYT						
2DL7*001 (H13)	E	A	N	G	S	P	L	L	T	N	Q	N	S	Q	S
2DL7*002 (v3 cDNA2)	D	V	D	.	N	S	.	.	.	.	.	.	.	E	.
2DL7*003 (v1 cDNA2)	.	.	.	S	N	S	F	F	.	.	.	.	.	E	.
2DL7*004 (v2 cDNA2)	.	.	.	.	N	S	F	F	I	K	H	D	N	R	I

H

Position	56	146	203	212	314	315	326	336	341	379	381	391	395	405	420
Domain	D0	D1	D2		S		TM		CYT						
3DL4*00101 (cDNA)	Q	H	P	T	I	R	A	L	R	Q	N	S	E	S	V
3DL4*00102 (v2 cDNA3)	.	.	.	.	.	.	.	.	.	.	.	.	.	.	.
3DL4*002 (T6)	R	.	S	.	N	P	V	F	C	H	D	K	Q	I	F
3DL4*003 (v1 cDNA2)	R	L	.	.	N	S	.	F	.	H	D	.	R	I	.
3DL4*004 (v1 cDNA3)	-	.	.	M	N	S	.	F	.	H	D	.	R	I	.

I

Position	158	219	220	231	232	236	241	250	266	268	284	286	296	300	310
Domain	D2	S		TM				CYT							
2DL8*001 (T4)	G	I	R	V	A	F	L	N	I	K	H	D	N	R	I
2DL8*002 (T5/v1 cDNA3)	.	N	S	A	V	L	F	K	.	.	.	.	.	.	.
2DL8*003 (T7)	R	N	S	A	V	L	F	K	T	N	Q	N	S	E	S

J

Position	56	146	149	218	233	240	243	316	342	348
Domain	D0	D1		D2				S	CYT	
3DS2*001 (cDNA)	Q	H	G	S	H	A	L	K	C	N
3DS2*002 (H8)	R	L	E	N	R	S	R	.	.	(FS)
3DS2*003 (v1 cDNA2)	R	.	E	.	.	.	.	R	W	(FS)

K

Position	-4	147	297	298	310	319	329	337	342	346	364	366	371
Domain	L	D1	S		TM		CYT						
3DL3*001 (T3)	G	V	I	R	V	L	K	Q	N	S	N	C	R
3DL3*002 (T2)	R	.	.	.	.	.	.	.	K	K	D	R	(FS)
3DL3*003 (T1)	.	I	N	P	A	F	.	*					
3DL3*004 (cDNA)	.	I	N	P	A	F	E	*					

L

Position	105	165	236	314	315	331	345	361	363	379	381	386	391	395	405	406
Domain	D1		D2	S		TM	CYT									
3DL5*001 (cDNA)	F	T	G	N	S	L	K	I	K	H	D	T	N	R	I	V
3DL5*00201 (v1 cDNA2)	L	.	R	.	.	.	.	.	.	.	.	.	.	.	.	.
3DL5*00202 (v2 cDNA2)	L	.	R	.	.	.	.	.	.	.	.	.	.	.	.	.
3DL5*003 (T7/v1 cDNA3)	L	M	R	I	R	F	N	T	N	Q	N	.	S	E	S	.
3DL5*004 (v2 cDNA3)	L	M	R	I	R	F	N	T	N	Q	N	.	S	E	S	E
3DL5*005 (v3 cDNA3)	L	M	R	I	R	F	N	T	N	Q	N	A	S	E	S	.
