# Supplementary material for: Human-Specific Evolution and Adaptation Led to Major Qualitative Differences in the Variable Receptors of Human and Chimpanzee Natural Killer Cells
Source: PLoS Genet. 2010 Nov 4;6(11):e1001192. doi: 10.1371/journal.pgen.1001192 (PMC2973822; doi:10.1371/journal.pgen.1001192)
Supplement: Text S1 — Supporting text. (0.19 MB PDF) [file pgen.1001192.s015.pdf]

Human-specific Evolution and Adaptation led to Major Qualitative Differences in the Variable Receptors of Human and Chimpanzee Natural Killer Cells

Laurent Abi-Rached[1], Achim K Moesta[1], Raja Rajalingam[2], Lisbeth A Guethlein[1] and Peter Parham[1]

[1]Department of Structural Biology, Stanford University School of Medicine, Stanford, California 94305, USA.

[2]UCLA Immunogenetics Center, Department of Pathology and Laboratory Medicine, David Geffen School of Medicine at UCLA, University of California at Los Angeles, Los Angeles, CA 90095, USA.

Text S1

Chimpanzee *KIR* haplotypes.
KIR expression study.
KIR genomic analyses.
KIR nomenclature changes in chimpanzees.
Pt-KIR3DS6 sequence and position.
Pt-KIR3DS2 allelic variation.
Chimpanzee *KIR* genotyping.
Haplotype predictions in chimpanzee.
MHC-B and *-C* phylogenetic analysis and ancestral sequences.
Selection analysis.

Text S1

Chimpanzee *KIR* haplotypes

To obtain the chimpanzee *KIR* haplotype H13, the CHORI RPCI-43 BAC library was screened with a ^{32}P-labeled probe containing a mix of Pt-KIR2DL4, Pt-KIR3DL4 and Pt-KIR3DL1/2 cDNA. The *KIR* gene content of the clones was determined by typing [1] and the clone with the largest complete *KIR* haplotype selected for sequencing (RP43-84K19). To facilitate sequencing, the insert was first digested with *NotI* and the four *NotI* fragments subcloned before shotgun libraries were prepared (TOPO Shotgun Subcloning Kit, Invitrogen). The sequences of the four *NotI* fragments were joined by direct sequencing on the original insert. Sequencing was performed on an ABI377 DNA sequencer (Applied Biosystems) and sequences were assembled with the STADEN package [2]. Each base pair of the final assembly was covered in both directions, by at least three sequences from at least two different templates and had a quality >40 (error rate <1/10,000). The final sequence was deposited in Genbank (accession number HM068617).

KIR expression study

RNA was extracted from peripheral blood mononuclear cells of chimpanzees Donald and Clint using Trizol reagent (Invitrogen). cDNA was prepared using the SuperScript First Strand Synthesis kit (Invitrogen). Generic primers Pt-KIR-All-LIII-F (5'CCTGTCTGCACCGGCAGC3') and Pt-KIR-All-LIII-R (5'GGAGAGGTGGGCAGGGGTC3') were used for PCR amplifications and the 2D and 3D KIR were isolated by gel electrophoresis, cloned into the TOPO vector (Invitrogen) and ~100 clones for Clint and ~200 clones for Donald sequenced. For Pt-KIR3DL5T7, the following primers were used: 5'GCTGTGGTGTCTGAAGGAGAAT3' (forward) and 5'CTCAGTGTGATCGCAGTCTCAA3' (reverse).

KIR genomic analyses

Gene sequences were aligned with MAFFT [3] and corrected manually. The alignment was then divided into fourteen segments, as previously described [4]. Each segment was analyzed with four methods: Bayesian, maximum-likelihood (ML), neighbor-joining (NJ) and parsimony. NJ phylogenetic analysis was performed with MEGA4 [5] using the Maximum Composite Likelihood method with 500 replicates. PAUP*4.0b10 [6] and the tree bisection-reconnection branch swapping algorithm were used for parsimony analyses with 500 replicates and a heuristic search. ML analyses were performed with RAXML7 [7] under the GTR+CAT model with 500 replicates (rapid bootstrapping). For the Bayesian analysis we selected the model of DNA substitution using MODELTEST3.7 [8] and the Akaike information criterion. Bayesian phylogenetic analyses used MRBAYES3.1.2 [9]; sampling was performed with one cold chain and three heated chains, which were run for 2,000,000 generations. Trees were sampled every 250 generations and the first 4,000 trees were discarded before a consensus tree was generated. In all analyses the average standard deviation of split frequencies for the three runs was <0.01.

Recombination breakpoints in chimpanzees were identified using RDP [10] and confirmed by phylogenetic analyses and alignment inspection.

For the lineage III *KIR* genes, a full gene analysis was performed on all fourteen segments; the *Pt-KIR3DS6* genomic sequence used in this analysis was obtained from the assembly of the chimpanzee genome available from the UCSC genome browser (March 2006 build; http://genome.ucsc.edu). This sequence lacks only ~1.5kb at the end of the gene (approximately the region from intron 7 to the 3'UTR).

KIR nomenclature changes in chimpanzees

Pt-KIR3DL1/2 and *Pt-KIR3DL3* were renamed *Pt-KIR3DL1/2a* and *Pt-KIR3DL1/2b,* respectively, an allelic relationship supported both by segregation analyses [1] and by sequencing (Figure 1A). This

change also eliminates confusion between this telomeric framework gene of the chimpanzee *KIR* locus (now named *Pt-KIR3DL1/2*) and *KIR3DL3* the centromeric framework gene of the human *KIR* locus. Consistent with this rationalization, *Pt-KIRC1*, the centromeric framework gene of the chimpanzee *KIR* locus and orthologous to human *KIR3DL3* in the five exons encoding leader sequence and Ig domains was renamed *Pt-KIR3DL3*. The sequence characterized as *Pt-KIR3DL6* in an analysis of *KIR* cDNA sequences in chimpanzees [1] was renamed *Pt-KIR3DS6* following re-analysis of the cytoplasmic tail sequence of this *KIR*, as described in the following section.

***Pt-KIR3DS6* sequence and position**

While investigating *KIR* expression in chimpanzee Clint (see paragraph 'KIR expression study'), a *KIR* was identified that had sequence identical to that of the *Pt-KIR3DL6* cDNA [1] except for insertion of one base pair at the end of exon 7. This inserted adenosine, part of a run of eight adenosines, changed the reading frame, leading to an early stop codon and loss of the two ITIM in the cytoplasmic tail of Pt-KIR3DL6. Because this sequence encodes an activating receptor it was named Pt-KIR3DS6 (Figure S10A). An independent observation of *Pt-KIR3DS6* has been made (Genbank accession numbers AM292663 and AM396937).

To see if *Pt-KIR3DL6* and *Pt-KIR3DS6* represent distinct alleles, we characterized the genomic sequences of *Pt-KIR3DL6/S6* from exon 7 to exon 9 in six individuals typing positively for *Pt-KIR3DL6*, including Alex, an individual from which *Pt-KIR3DL6* was first characterized [1]. PCR amplification was performed in duplicate with the following primers: 5'GGTCAAAATCCCTTTCACCA3' (STK_F, forward) and 5'GCTCAGCATTTGGAGGTTCTA3' (Pt-KIR3DS6_R1, reverse). PCR products were cloned and several clones sequenced for each individual and from each amplification (Figure S10B). For all individuals, clones with 8 adenosines in exon 7 (*Pt-KIR3DS6*) represented the majority and clones with 7 adenosines (*Pt-KIR3DL6*) the minority, consistent with the latter likely being artifacts of PCR slippage [11].

Because *Pt-KIR3DS6* is the only *KIR* not represented in the three sequenced haplotypes of Figure 1A, we investigated its position using long-range PCR (Figure S10C). Primers, 5'GGTCAAAATCCCTTTCACCA3' (3DS6_POS_F1; forward) and 5'GATCACCAGGGGGTTGCT3' (3DS6_POS_R1; reverse), were used to amplify the genomic segments between exon 7 of an activating KIR and exon 3 of the 5' adjacent KIR in four individuals with *Pt-KIR3DS6* (Buckwheat, Mahoney, Mouse and Reggie). Amplification with the BD Advantage DNA polymerase (BD Biosciences, Clontech) produced good yield for chimpanzees Mahoney, Mouse and Reggie. End-sequencing cloned PCR products showed that the position of *Pt-KIR3DS6* was 5' of *Pt-KIR2DL6* in these individuals.

Pt-KIR3DS2 allelic variation

In the course of improving *Pt-KIR3DS2* genotyping, the old 'F0/R0' and the new typing primers (named 'F1/R1) were found to give some inconsistencies. Five individuals (Eve, Miss Eve, Buckwheat, Lucas and Duff) typed positively with 'F0/R0' and negatively with 'F1/R1'. Sequencing of the 'F0/R0' PCR products (that target the genomic region around the exons encoding the Ig domains) of Eve and Miss Eve revealed a sequence related to *Pt-KIR3DL4* and *3DS2* but these sequences could not be unambiguously assigned to one particular group without the sequence of exons encoding the transmembrane and cytoplasmic tail. For Lucas and Duff, for whom peripheral blood mononuclear cells were available, *KIR* cDNA sequences were amplified as described in the 'KIR expression study' section above. This analysis showed that both individuals have *3DS2*, but the alleles were highly related to *Pt-KIR3DL4* for the 5' exons (encoding extracellular domains) and to *Pt-KIR2DS4* for the 3' exons (encoding transmembrane and cytoplasmic domains). However, these novel *3DS2* sequences shared unique substitutions with previously characterized *3DS2* sequences in exon 6, encoding the Stem. Because these single-nucleotide polymorphisms appeared unique to *Pt-KIR3DS2*, this property was used to design a 'Nested' primer set.

Use of the ‘Nested’ primer set revealed positive results for four of the five individuals found to be positive with ‘F0/R0’ but not with ‘F1/R1: Miss Eve, Buckwheat, Lucas and Duff. For Eve, generic amplification for the presence of an activating tail failed, as well as a gene-to-gene amplification designed to characterize activating tails (not shown); consequently, chimpanzee Eve was considered *Pt-KIR3DS2* negative. As a consequence of these analyses, typing for *Pt-KIR3DS2* required three amplifications: most alleles type positively with ‘F1/R1’, but those that do not type positively with both ‘F0/R0’ and ‘Nested’ primers (see details in ‘KIR typing in chimpanzee’ section below).

Chimpanzee *KIR* genotyping

The sequence-specific primer (SSP) method was used. *Pt-KIR2DL4*, *2DL5*, *3DL1/2a* and *b*, and *3DL5* were typed using the primers previously described [1], new primers were developed for the other nine *KIR*. Presence of each *KIR* was assessed at least twice for each individual, either by repeat amplification with the same primer set or with a different primer set; when positive reactions were observed with one primer set but not with a second, the products were cloned and sequenced to confirm the specificity. When possible, primer sets were combined to produce a single set that amplifies all alleles of the targeted *KIR*. Unless otherwise mentioned, all the new PCR amplifications used the following cycling parameters: initial denaturation at 95°C for 3min, then 32 cycles with a denaturation at 95°C for 20sec, an annealing at 63°C for 45sec, and an extension at 72°C for 2min.

Pt-KIR2DL6. Amplification was performed with primers 5’ GCCTCTAGGACATGTCATTCTTT3’ (Pt-KIR2DL6-F1; forward) and 5’TCACTGGGAGCTGACAACA3’ (Pt-KIR2DL6-R1; reverse).

Pt-KIR2DL7. Amplification was performed with primers 5’CCATCGGTCTCATGACGAA3’ (Pt-KIR2DL7-F1; forward) and 5’GGAAAGTCTGCCTGGAATGTC3’ (Pt-KIR2DL7-R2; reverse).

Pt-KIR2DL8. Two primer sets were used, a positive result with either set indicating presence of *Pt-KIR2DL8*. The first set uses primers 5'CATCCTGCAATGTTGGTCG3' (Pt-KIR2DL8-F1; forward) and 5'AAGCAGTGGGTCACTCGGA3' (Pt-KIR2DL8-R1; reverse). The second set uses primers 5'TCTCCCCAAGGTGGTCAGA3' (Pt-KIR2DL8-F2; forward) and 5' CATGGAGCTCTCCAGTGAGG3' (Pt-KIR2DL8-R2b; reverse); annealing is performed at 65°C.

Pt-KIR2DL9. Amplification was performed with primers 5'TGTAACGACACTTTGCACGC3' (Pt-KIR2DL9-F1; forward) and 5' CTGGAATGTTCCATTGATGGT3' (Pt-KIR2DL9-R1; reverse).

Pt-KIR2DS4. Amplification was performed with primers 5'TGTGACCTTGTCCTGCAGT3' (Pt-KIR2DS4-D2-F1; forward) and 5'TGACCACTCGTAGGGAGC3' (Pt-KIR2DS4-D2-R1; reverse).

Pt-KIR3DL4. Amplification was performed with primers 5'CCCACTGAACCAAGCACT3' (Pt-KIR3DL4-All-NF2; forward) and 5'TTGCTGTCACCTTGATTTTAGC3' (Pt-KIR3DL4-All-NR1; reverse).

Pt-KIR3DS6. Amplification was performed with primers 5'TGCTCGGGACATATGGAT3' (Pt-KIR3DS6-I6/E7-F0; forward) and 5'GGATGGTGAAAGGGATTTTG3' (Pt-KIR3DS6-I6/E7-R1; reverse).

Pt-KIR3DL3. Amplification was performed with primers 5'GGTGAAATCGGGAGAGACG3' (Pt-KIR3DL3-D1-F1; forward) and 5'CACTGGGAGCCGACAACTC3' (Pt-KIR3DL3-D1-R1; reverse).

Pt-KIR3DS2. The primer set 'F1/R1' characterizes most *Pt-KIR3DS2* alleles but additional alleles are detected as being positives with both 'F0/R0' and 'nested' primers (see also the '*Pt-KIR3DS2* allelic variation' section above).

Amplification 'F1/R1' was performed with primers 5'CTTTCTCCTTCATCGCTGC3' (Pt-KIR3DS2-F1; forward) and 5' ACCTCCTGAGAGTCTTGTTCAA 3' (Pt-KIR3DS2-R1; reverse).

Amplification 'F0/R0' is performed with the primers and conditions described in the study of chimpanzee *KIR* cDNA sequences [1].

The 'nested' amplification was performed with an annealing temperature of 62°C and consisted of a first amplification (15 cycles) with primers 5'TACGTGTTTCATAGGTTCAG3' (Pt-KIR3DS2_All_NF1; forward) and 5'TAAGAGGGTTCTGTACTCACT3' (Pt-KIR3DS2_All_NR1; reverse). After a 1/10 dilution, a second amplification (25 cycles) was conducted with primers 5'GCCAGGAGTTCAAGATCAGC3' (Pt-KIR3DS2_All_NF2; forward) and 5'TCACTGGTTTTGGAGCTTGA3' (Pt-KIR3DS2_All_NR2; reverse).

Haplotype predictions in chimpanzee

Reconstruction of haplotype structures from genotype data was performed with HAPLO-IHP [12]. For this analysis, genotypic data were used: from the 39 individuals of Figure 1B**,** as well as the three reference haplotypes of Figure 1A. *Pt-KIR3DL3*, *2DL4* and *3DL1/2* were set as framework genes. The two *KIR* haplotypes of Donald (a member of the panel) were fully sequenced so his *KIR* genotype was entered with inclusion of the copy number for each *KIR*. Sequences of two *Pt-KIR3DL4* alleles defined by cDNA clones obtained from chimpanzee Lucas were also included.

Since none of the three sequenced chimpanzee haplotypes have all 13 *KIR* genes, the relative position of some *KIR* genes is ambiguous: first, the position of *Pt-KIR3DL5* is not known when it belongs to a haplotype with *3DL4* and/or with *2DS4-2DL7*; second, the position of *3DS6-2DL6* is not known when they belong to haplotypes with *3DS2-2DL9*. The gene order used in Figures 1 and 2 is thus arbitrary for these *KIR*.

***MHC-B* and *-C* phylogenetic analysis and ancestral sequences**

For the MHC sequence analysis, we gathered all non-human hominoid *MHC-B* and *-C* and non-macaque Old World monkey *MHC-B* sequences from the IPD-MHC database [13]. Human sequences were

obtained from the IMGT/HLA database [14] and included one to three sequences for each *HLA-B* and -*C* sublineage as well as all *HLA-B* alleles encoding allotypes with V76. Macaque sequences were obtained from studies in *Macaca mulatta* [15] and *Macaca fascicularis* [16]. *MHC-E* sequences were used as the outgroup. Sequences with recombinant or missing segments in the α1 or α2 were excluded, recombinant α3 domains were masked. Alignment of the α1, α2 and α3 domains and phylogenetic analyses were conducted as described for *KIR* genomic analyses. For the Bayesian analysis three independent long runs were performed (10,000,000 generations) and the final average standard deviation of split frequencies was <0.04.

Ancestral sequences were reconstructed with CODEML of the PAML4 software package [17], using the marginal reconstruction approach. Two codon models were used for this analysis: M0, the simplest model (implements a single dN/dS rate), and M2a, a model allowing for positive selection (three dN/dS categories). Analysis was first performed with the simplest model (M0), and when nodes were not fully resolved by M0 (p>0.95), reconstruction was also performed with model M2a.

Selection analysis

For selection analyses, dN/dS (ω) ratios were estimated by maximum likelihood using PAML4 [17] with the F3X4 model of codon frequencies. ML tree topologies (generated using the approach described in the 'KIR Genomic Analyses') were used for these analyses and three sets of likelihood ratio tests were conducted to compare null models that do not allow ω>1 (M1a, M7 and M8a) with models that do (M2a and M8). Significance was assessed by comparing twice the difference in likelihood between the models (2ΔL) to a χ2 distribution with one (M8a/M8) or two (M1a/M2 and M7/M8) degrees of freedom. Codons with ω>1 were identified using the Bayes Empirical Bayes approach [18]. Positively selected positions in D1 and D2 were marked in the KIR2DL2-HLA-Cw3 three-dimensional structure (PDB file 1EFX [19]) using the software PyMOL [20].

References

1. Khakoo SI, Rajalingam R, Shum BP, Weidenbach K, Flodin L, et al. (2000) Rapid evolution of NK cell receptor systems demonstrated by comparison of chimpanzees and humans. Immunity 12: 687-698.
2. Staden R, Beal KF, Bonfield JK (2000) The Staden package, 1998. Methods Mol Biol 132: 115-130.
3. Katoh K, Misawa K, Kuma K, Miyata T (2002) MAFFT: a novel method for rapid multiple sequence alignment based on fast Fourier transform. Nucleic Acids Res 30: 3059-3066.
4. Guethlein LA, Older Aguilar AM, Abi-Rached L, Parham P (2007) Evolution of killer cell Ig-like receptor (KIR) genes: definition of an orangutan KIR haplotype reveals expansion of lineage III KIR associated with the emergence of MHC-C. J Immunol 179: 491-504.
5. Kumar S, Tamura K, Nei M (2004) MEGA3: Integrated software for Molecular Evolutionary Genetics Analysis and sequence alignment. Brief Bioinform 5: 150-163.
6. Swofford DL (2001) PAUP*: Phylogenetic analysis using parsimony (*and other methods), version 4.0.: Sinauer, Sunderland, Massachusetts.
7. Stamatakis A (2006) RAxML-VI-HPC: maximum likelihood-based phylogenetic analyses with thousands of taxa and mixed models. Bioinformatics 22: 2688-2690.
8. Posada D, Crandall KA (1998) MODELTEST: testing the model of DNA substitution. Bioinformatics 14: 817-818.
9. Ronquist F, Huelsenbeck JP (2003) MrBayes 3: Bayesian phylogenetic inference under mixed models. Bioinformatics 19: 1572-1574.
10. Martin DP, Williamson C, Posada D (2005) RDP2: recombination detection and analysis from sequence alignments. Bioinformatics 21: 260-262.
11. Shinde D, Lai Y, Sun F, Arnheim N (2003) Taq DNA polymerase slippage mutation rates measured by PCR and quasi-likelihood analysis: (CA/GT)n and (A/T)n microsatellites. Nucleic Acids Res 31: 974-980.
12. Yoo YJ, Tang J, Kaslow RA, Zhang K (2007) Haplotype inference for present-absent genotype data using previously identified haplotypes and haplotype patterns. Bioinformatics 23: 2399-2406.
13. Robinson J, Waller MJ, Stoehr P, Marsh SG (2005) IPD--the Immuno Polymorphism Database. Nucleic Acids Res 33: D523-526.
14. Robinson J, Waller MJ, Parham P, de Groot N, Bontrop R, et al. (2003) IMGT/HLA and IMGT/MHC: sequence databases for the study of the major histocompatibility complex. Nucleic Acids Res 31: 311-314.

15. Karl JA, Wiseman RW, Campbell KJ, Blasky AJ, Hughes AL, et al. (2008) Identification of MHC class I sequences in Chinese-origin rhesus macaques. Immunogenetics 60: 37-46.

16. Krebs KC, Jin Z, Rudersdorf R, Hughes AL, O'Connor DH (2005) Unusually high frequency MHC class I alleles in Mauritian origin cynomolgus macaques. J Immunol 175: 5230-5239.

17. Yang Z (1997) PAML: a program package for phylogenetic analysis by maximum likelihood. Comput Appl Biosci 13: 555-556.

18. Yang Z, Wong WS, Nielsen R (2005) Bayes empirical bayes inference of amino acid sites under positive selection. Mol Biol Evol 22: 1107-1118.

19. Boyington JC, Motyka SA, Schuck P, Brooks AG, Sun PD (2000) Crystal structure of an NK cell immunoglobulin-like receptor in complex with its class I MHC ligand. Nature 405: 537-543.

20. DeLano WL (2002) The PyMOL Molecular Graphics System. DeLano Scientific, San Carlos, CA, USA.
